# Potential drug targets for systemic lupus erythematosus identified through Mendelian randomization analysis

**DOI:** 10.1097/MD.0000000000041439

**Published:** 2025-02-14

**Authors:** Shiwen Fan, Kaixin Wang, Shuai Wang, Xiangdong Chen

**Affiliations:** a Department of Anesthesiology, Union Hospital, Tongji Medical College, Huazhong University of Science and Technology, Wuhan, China; b Institute of Anesthesia and Critical Care Medicine, Union Hospital, Tongji Medical College, Huazhong University of Science and Technology, Wuhan, China; c Key Laboratory of Anesthesiology and Resuscitation (Huazhong University of Science and Technology), Ministry of Education, Wuhan, China; d Department of Anesthesiology, First Affiliated Hospital of Shihezi University, Shihezi, China; e Department of Gastric and Colorectal Surgery, General Surgery Center, The First Hospital of Jilin University, Changchun, Jilin, China.

**Keywords:** drug target, Mendelian randomization, protein quantitative trait loci, systemic lupus erythematosus

## Abstract

So far, there is no clear pathogenesis and no cure for systemic lupus erythematosus (SLE). The therapeutic benefits of existing drug therapies are far from ideal. The proteome is a major source of therapeutic targets. Therefore, new drug targets for SLE need to be discovered. Based on the STROBE-Mendelian randomization (MR) checklist, we performed MR to explore potential drug targets for SLE, using genome-wide association study summary statistics of plasma and cerebrospinal fluid (CSF) and further replicated in the external validation. Bidirectional MR, reverse causality testing by Steiger filtering, Bayesian co-localization were used. In addition, protein–protein interaction networks (PPI) were performed to reveal potential associations between proteins and current SLE drugs. At false discovery rate (FDR) significance (*P*_*FDR*_ < .05), MR analysis revealed 8 proteins. Five proteins decreased the SLE risks, whereas the other 3 proteins increased the SLE risks. None of the 8 proteins had reverse causality except sICAM-1. Bayesian co-localization suggested that 5 proteins shared the same variant with SLE. PPI network suggested that intercellular adhesion molecular 1 (ICAM-1), Fc-gamma-RIIb (FCG2B) and N-terminal pro-B-type natriuretic peptide (N-terminal pro-BNP) interacted with targets of current SLE medications. Our integrative analysis revealed that SLE risk is causally associated with ICAM-1, FCG2B, and N-terminal pro-BNP. These 3 proteins have the potential to become drug targets of SLE, especially for ICAM-1 and FCG2B. More further studies are also warranted to support this finding.

## 1. Introduction

Systemic lupus erythematosus (SLE), as a chronic autoimmune disease with diverse clinical manifestations and easy relapse and remission, is 1 of the most challenging diseases.^[1]^ SLE is characterized by systemic and organ-targeted damage that can involve the central nervous system, cardiovascular system, skin, joints, kidneys, and more.^[2–4]^

There is currently no cure for the disease, but early diagnosis and drug control are possible. The main goals of SLE treatment are to reduce disease activity, prevent irreversible organ damage, and maintain quality of life. At present, the drugs applied to treat SLE mainly include antimalarial drugs, glucocorticoids, nonsteroidal antiinflammatory drugs and immunosuppressants. However, these drugs have obvious side effects, such as cataracts, osteoporotic fractures, serious infections, malignant tumors, teratogenesis and more, and the treatment effect is not ideal.^[5–9]^

Mendelian randomization (MR) used genetic variants as an instrumental variable for the exposure to strength causal inference.^[10]^ MR is less susceptible to confounding because genetic variants are randomly assigned at conception and are therefore independent of environmental and self-administered factors.^[11]^ Drug target MR is a recently developed extension of the MR Paradigm that has been applied to predict the efficacy and potential adverse effects of therapeutic interventions in clinical trials.^[12]^ By using genetic variation as a proxy for the mechanistic effects of drug targets, the method allows analysis of protein function and drug target perturbations.

**Figure 1.**
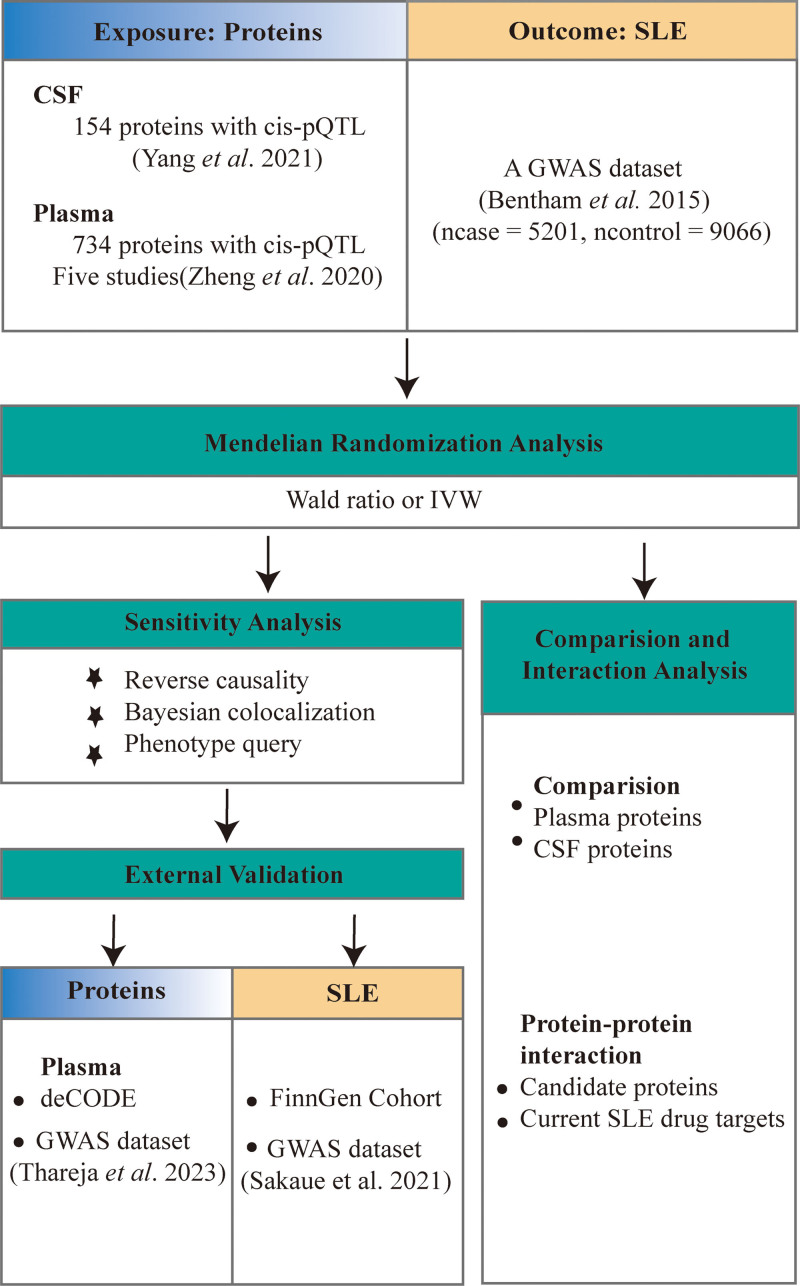
Study design for identification of plasma and CSF proteins causally associated with SLE. CSF = cerebrospinal fluid, deCODE = a database including 4907 plasma proteins reported by Ferkingstad et al, GWAS = genome-wide association study, IVW = inverse variance weighted, pQTL = protein quantitative trait loci, SLE = systemic lupus erythematosus.

To date, very few MR Studies have integrated SLE’s genome-wide association studies (GWAS) and protein quantitative trait locus (pQTL) data. In this study, we aimed to identify potential plasma and cerebrospinal fluid (CSF) proteins for the development of SLE. Figure 1 shows the study design (Fig. 1). First, MR analysis was used to identify potentially pathogenic plasma and CSF proteins in SLE by using SLE GWAS data,^[13]^ Plasma pQTL data^[14]^, and CSF pQTL data.^[15]^ Then, the main findings were further confirmed by reverse causality test, Bayesian co-localization analysis and phenotypic scanning. Third, protein-protein interaction (PPI) networks between plasma proteins and CSF proteins (primary *P* < .05), and between 8 candidate proteins and current SLE drug targets were mapped. Finally, external validation, using different databases, was performed to strengthen our conclusions.

## 2. Materials and methods

### 2.1. CSF and plasma protein quantitative trait loci

CSF pQTL data were obtained from a study,^[15]^ who reported 274 pQTLs of 184 CSF proteins. Only pQTLs satisfying the following criteria were included: showed genome-wide significant association (*P* < 5 × 10^−8^); were located outside the major histocompatibility complex region (chromosome6, 26–34 Mb); showed independent association (linkage disequilibrium (LD) clumping r^2^ < 0.001, kb = 10,000); and was a cis-acting pQTL. Finally, 154 cis-pQTLs were identified for 154 proteins.

For the primary analysis, the plasma pQTL data were retrieved from a study,^[14]^ which integrated 5 previously published GWAS.^[16–20]^ Based on the screening criteria used above in the CSF pQTL dataset, 738 cis-acting SNPs for 734 proteins were included. Data were checked using the original documents as a reference to ensure reliability. In addition, the plasma pQTL data for external validation were retrieved from 2 recently published studies.^[21,22]^

For any missing information in the QTL GWAS summary statistics, such as effect allele frequency, we used the matched human genome build as a reference to complete the data (Table S1, Supplementary Digital Content, http://links.lww.com/MD/O357).

### 2.2. GWAS summary statistics of SLE

For the primary analysis, summary statistics were retrieved from a GWAS dataset including 14,267 individuals (ncase = 5201, ncontrol = 9066) of European ancestry.^[13]^ For external validation, summary statistics were obtained from another GWAS dataset (ncase = 647, ncontrol = 482,264) of European ancestry^[23]^ and the FinnGen cohort (ncase = 1083, ncontrol = 306,504, R10 release) (https://finngen.gitbook.io/documentation/data-download).

### 2.3. Statistical analysis

In this study, we used the plasma and CSF proteins as the exposure and SLE as the outcome to perform MR with “TwoSampleMR” (https://github.com/MRCIEU/TwoSampleMR) in R 4.2.1 software. The Wald ratio was used if only 1 pQTL was available for a given protein. When 2 or more genetic instruments were available, inverse variance weighted (IVW) was used as the main analysis method. MR-Egger, weighted median, simple mode, and weighted mode were used as supplementary analysis methods. If the assumption that all included SNPs can be used as effective IVs is met, the IVW method provides an accurate estimate.^[24]^ MR-Egger regression can examine and adjust the pleiotropy, but the estimation accuracy conducted by this method is very low.^[25]^ Weighted median could provide an accurate estimate based on the assumption that at least 50% of IVs are valid.^[26]^ Although simple mode is not as powerful as IVW, but it provides robustness for pleiotropy.^[27]^ Weighted mode is sensitive to the difficult bandwidth selection for mode estimation.^[28]^ OR for increased risk of SLE were expressed as per standard deviation increase in plasma protein levels and per 10-fold increase in CSF protein levels. What is more, I^2^ index and Cochran *Q* statistic were adopted for IVW analysis and MR-egger analysis to detect the heterogeneity, and *P* > .05 indicates no heterogeneity.^[29]^

For the primary analysis, false discovery rate (FDR) correction was used to adjust for multiple testing, and a threshold *P*-value of .05 (*P*_*FDR*_ < .05) was used for CSF proteins and plasma proteins to prioritize the results for further analysis. MR was performed only on the preliminarily identified proteins for external validation and was set at a *P*-value threshold of .05.

Reverse causality detection following the same screening criteria for pQTLs, 44 genetic instruments for SLE were selected from the GWAS summary statistics^[13]^ for bidirectional MR analysis to detect potential reverse causality (Table S2, Supplemental Digital Content, http://links.lww.com/MD/O357).^[30]^ The effect was estimated using MR-IVW, MR-Egger, weighted median, simple mode and weighted mode. We also conducted steiger filtering^[31]^ to ensure the directionality of the association between proteins and SLE.^[32]^ The results were considered statistically significant at *P *< .05.

### 2.4. Bayesian co-localization analysis

Bayesian co-localization analyses were used to assess the probability that 2 traits share the same causal variant using the “coloc” package (https://github.com/chr1swallace/coloc) with default arguments. As described previously,^[20]^ Bayesian co-localization provides the posterior probability for 5 hypotheses on whether a single variant is shared between 2 traits. In this study, we tested the posterior probability of hypothesis 3, in which both the protein and SLE were associated with the region by different variants, and hypothesis 4 (PPH4), in which both the protein and SLE were associated with the region by shared variants. the coloc.abf algorithms were used, and we defined a gene as having evidence of co-localization based on gene-based PPH4 > 80%.^[33]^

### 2.5. Comparison analysis and protein–protein interaction network

We hypothesized that there would be little correlation between pQTLs identified in plasma and CSF due to the blood-brain barrier (BBB). Therefore, the correlation between the common pQTLs identified in CSF and plasma using the effect estimates from the MR analysis was examined by Spearman correlation analysis, and the different *P*-value thresholds were set to examine whether the correlation changed with increasing significance level. The PPI network of proteins suggestively associated with SLE risk (primary MR analysis *P *< .05) in CSF or plasma analysis was explored. We aimed to investigate the interactions among the prioritized proteins and whether the proteins identified using plasma data could interact with those identified using CSF data. In addition, to explore the interactions between those SLE-associated genes and the targets for medications already on the market, we obtained 18 disease-modifying drugs for SLE from a recent review^[34]^ and corresponding drug targets based on the Drugbank database (https://www.drugbank.ca).^[35]^ We also searched current medications targeting the identified potential causal proteins. All PPI analyses were conducted using the search tool for the retrieval of interacting genes (STRING) database version 11.5 (https://string-db.org/), with the minimum required interaction score at 0.4.^[36]^

### 2.6. Data availability

Genome-wide summary-level statistics for cis-pQTL were available from the original studies.^[16–20]^ GWAS summary statistics^[13]^ and external validation summary statistics^[23]^ could both be obtained from the free database website (https://www.ebi.ac.uk/gwas/). Access to FinnGen (R10 release) can be obtained from the website https://finngen.gitbook.io/documentation/data-download.

### 2.7. Ethical statement

This MR research utilized only published or publicly available GWAS data. Each participant received ethical approval and informed consent for the respective study, as detailed in the original publication and consortium.

## 3. Results

### 3.1. Screening the proteome for SLE causal proteins

**Figure 2.**
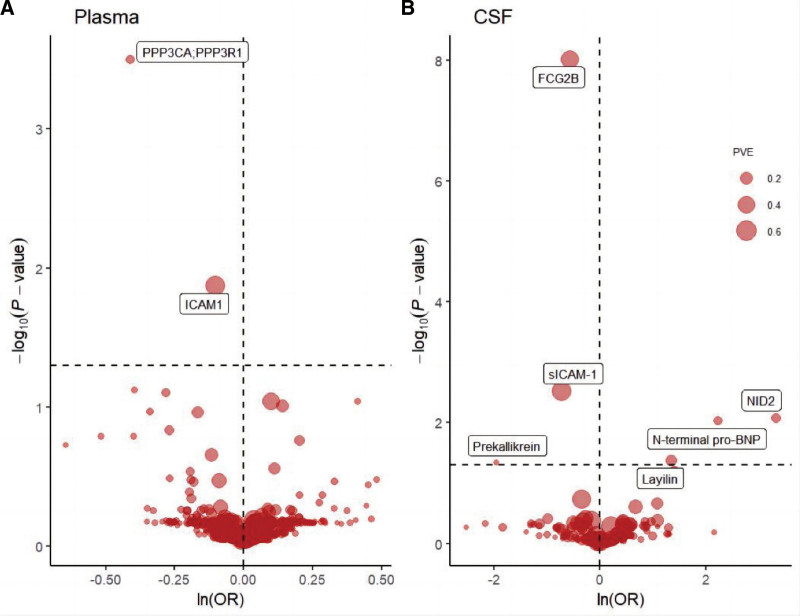
MR results for plasma and CSF proteins and the risk of SLE. Volcano plots of the MR results for (A) 734 plasma and (B) 154 CSF proteins on the risk of SLE. (A and B) show MR analysis with Wald ratio or inverse variance weighted method on plasma and CSF proteins on the risk of SLE, respectively. OR for increased risk of SLE were expressed as per SD increase in plasma protein levels and per 10-fold increase in CSF protein levels. Dashed horizontal black line corresponded to *P*_*FDR*_ = .05. ln = natural logarithm; PVE = proportion of variance explained. OR = odds ratio, PVE = proportion of variance explained, PPP3CA = calcineurin A, ICAM-1 = intercellular adhesion molecular 1, FCG2B, Fc-gamma-RIIb; sICAM-1, soluble intercellular adhesion molecule 1, NID2 = Nidogen 2, N-terminal pro-BNP = N-terminal pro-B-type natriuretic peptide.

**Figure 3.**
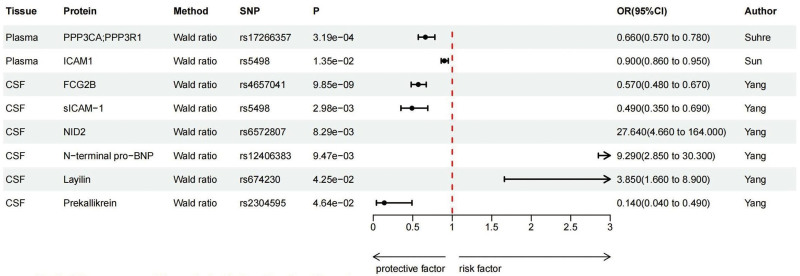
Forest map on the causal relationship of 8 potential causal proteins on SLE by MR analysis. CI = confidence interval, CSF = cerebrospinal fluid, FCG2B = Fc-gamma-RIIb, ICAM-1 = intercellular adhesion molecular 1, NID2 = Nidogen 2, N-terminal pro-BNP = N-terminal pro-B-type natriuretic peptide, OR = odds ratio, PPP3CA = calcineurin A, sICAM-1 = soluble intercellular adhesion molecule 1, SNP = single nucleotide polymorphism.

At FDR significance (*P*_*FDR*_ < .05), MR analysis revealed 8 protein–SLE pairs (Table 1, Figs. 2A and B and 3), including calcineurin A (PPP3CA;PPP3R1), intercellular adhesion molecular 1 (ICAM-1) in the plasma; Fc-gamma-RIIb (FCG2B), soluble intercellular adhesion molecule 1 (sICAM-1), Nidogen 2 (NID2), N-terminal pro-B-type natriuretic peptide (N-terminal pro-BNP), Layilin and Prekallikrein in the CSF. Specifically, increased PPP3CA/PPP3R1 (OR = 0.660; 95% CI = 0.570–0.780; *PFDR *= 3.19 × 10^−4^), ICAM-1 (OR = 0.900; 95% CI = 0.860–0.950; *PFDR* = 1.35 × 10^−2^), FCG2B (OR = 0.570; 95% CI = 0.480–0.670; *PFDR *= 9.85 × 10^−9^), sICAM-1 (OR = 0.490; 95% CI = 0.350–0.690; *PFDR *= 2.98 × 10^−3^) and Prekallikrein (OR = 0.140; 95% CI = 0.040–0.490; *P*_*FDR*_ = 4.64 × 10^−2^) decreased the risk of SLE, whereas elevated NID2 (OR = 27.640; 95% CI = 4.660–164.000; *P*_*FDR*_* *= 8.29 × 10^−3^), N-terminal pro-BNP (OR = 9.290; 95%CI = 2.850–30.300; *P*_*FDR*_* *= 9.47 × 10^−3^) and Layilin (OR = 3.850; 95% CI = 1.660–8.900; *P*_*FDR*_* *= 4.25 × 10^−2^)increased the risk of SLE. No heterogeneity was detected for the proteins analyzed in the primary analysis (Table S3, Supplemental Digital Content, http://links.lww.com/MD/O357).

**Table 1:** MR results for plasma and CSF proteins significantly associated with SLE after FDR correction.

Tissue	Protein	UniProt ID	SNP	Effect allele	OR (95% CI)	*P*_*FDR*_-value	PVE	*F*-statistics	Author
Plasma	PPP3CA; PPP3R1	A0A0S2Z4C6; Q08209; A0A0S2Z4B5; P63098	rs17266357	C	0.66 (0.57–0.78)	3.19 × 10^−4^	6.29%	66.9	Suhre
Plasma	ICAM-1	P05362	rs5498	G	0.90 (0.86–0.95)	1.35 × 10^−2^	70.18%	7769.9	Sun
CSF	FCG2B	P31994	rs4657041	T	0.57 (0.48–0.67)	9.85 × 10^−9^	47.57%	757.73	Yang
CSF	sICAM-1	P05362	rs5498	G	0.49 (0.35–0.69)	2.98 × 10^−3^	57.39%	1124.84	Yang
CSF	NID2	Q14112	rs6572807	G	27.64 (4.66–163.87)	8.29 × 10^−3^	9.62%	88.89	Yang
CSF	N-terminal pro-BNP	P16860	rs12406383	C	9.29 (2.85–30.29)	9.47 × 10^−3^	8.69%	79.46	Yang
CSF	Layilin	Q6UX15	rs674230	G	3.85 (1.66–8.90)	4.25 × 10^−2^	14.18%	137.92	Yang
CSF	Prekallikrein	P03952	rs2304595	G	0.14 (0.04–0.49)	4.64 × 10^−2^	4.11%	35.76	Yang

CI = confidence interval, CSF = cerebrospinal fluid, FCG2B = Fc-gamma-RIIb, FDR = false discovery rate, ICAM-1 = intercellular adhesion molecular 1, IVW = inverse variance weighted, MR = mendelian randomization, NID2 = Nidogen 2, N-terminal pro-BNP = N-terminal pro-B-type natriuretic peptide, OR = odds ratio, PPP3CA = calcineurin A, PVE = proportion of variance explained, sICAM-1 = soluble intercellular adhesion molecule 1, SLE = systemic lupus erythematosus, SNP = single nucleotide polymorphism.

### 3.2. Sensitivity analysis for causal proteins of SLE

**Figure 4.**
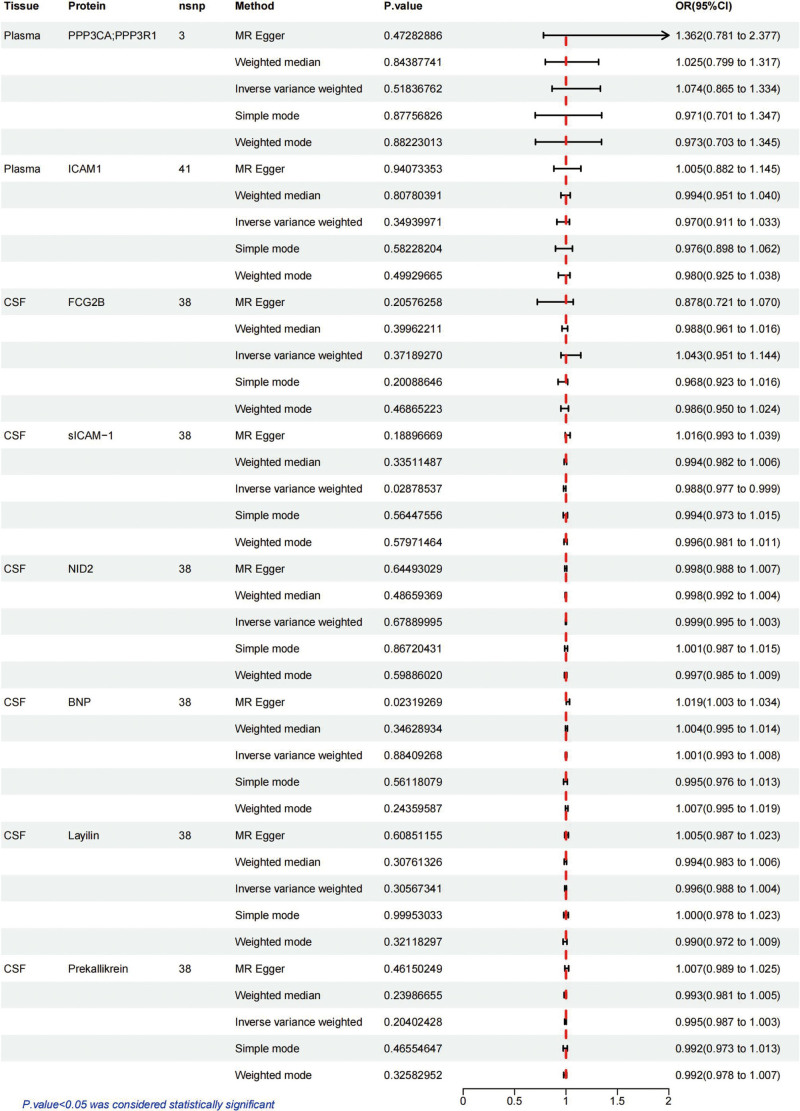
Bidirectional MR analysis for SLE on levels of 8 potential causal proteins. OR stood for the odds ratios for per standard deviation (SD) increase in plasma protein levels and per 10-fold increase in CSF protein levels as SLE risk increased. CI = confidence interval, CSF = cerebrospinal fluid, FCG2B = Fc-gamma-RIIb, ICAM-1 = intercellular adhesion molecular 1, NID2 = Nidogen 2, N-terminal pro-BNP = N-terminal pro-B-type natriuretic peptide, OR = odds ratio, PPP3CA = calcineurin A, sICAM-1 = soluble intercellular adhesion molecule 1, SNP = single nucleotide polymorphism.

Four of the 8 proteins revealed by the MR analysis were identified as potential drug targets for SLE, including ICAM-1, FCG2B, N-terminal pro-BNP and Layilin. First, bidirectional MR analysis was used to demonstrate any causal effect between SLE and the 8 major identified proteins, with Steiger filtering further assuring directivity (Table 2 and Fig. 4). We only found a bidirectional causal association between SLE and CSF sICAM-1. Second, Bayesian co-localization strongly suggested that ICAM-1 (coloc.abf-PPH4 = 1.000), FCG2B (coloc.abf-PPH4 = 0.996), sICAM-1 (coloc.abf-PPH4 = 1.000), N-terminal pro-BNP (coloc.abf-PPH4 = 0.844) and Layilin (coloc.abf-PPH4 = 0.942) shared the same variant with SLE (Table 2 and Fig. S1, Supplemental Digital Content, http://links.lww.com/MD/O356). Finally, we queried the possible phenotypes for these SNPs by LD trait (https://ldlink.nci.nih.gov/?tab=ldtrait).^[37]^ No confounding or outcome correlation was found among these SNPs through LD trait, apart from NID2 (rs6572807). rs6572807 was found to be associated with triglyceride measurement, body mass index (BMI)-adjusted waist circumference and triglyceride cholesterol ratio.

**Table 2:** Summary of reverse causality detection, Bayesian co-localization analysis on 8 potential causal proteins.

Tissue	Protein	UniProt ID	SNP	*Pval* (Bidirectional MR; MR-IVW)	Steiger filtering	Co-localization PPH4 (coloc.abf)
Plasma	PPP3CA; PPP3R1	P63098	rs17266357	.518367616	Passed (6.145065 × 10^−11^)	3.21 × 10^−5^
Plasma	ICAM-1	P05362	rs5498	.349399708	Passed (0.000000)	1.000
CSF	FCG2B	P31994	rs4657041	.371892701	Passed (4.455123 × 10^−7^)	0.996
CSF	sICAM-1	P05362	rs5498	.028785371	Passed (1.038749 × 10^−158^)	1.000
CSF	NID2	Q14112	rs6572807	.67889995	Passed (1.854260 × 10^−14^)	0.713
CSF	N-terminal pro-BNP	P16860	rs12406383	.884092683	Passed (9.526563 × 10^−110^)	0.844
CSF	Layilin	Q6UX15	rs674230	.305673405	Passed (3.775806 × 10^−16^)	0.942
CSF	Prekallikrein	P03952	rs2304595	.20402428	Passed (3.164819 × 10^−25^)	4.56 × 10^−5^

CSF = cerebrospinal fluid, FCG2B = Fc-gamma-RIIb, ICAM-1 = intercellular adhesion molecular 1, IVW = inverse variance weighted, MR = Mendelian randomization, NID2 = Nidogen 2, N-terminal pro-BNP = N-terminal pro-B-type natriuretic peptide, PPH4 = probability of hypothesis 4, PPP3CA = calcineurin A, sICAM-1 = soluble intercellular adhesion molecule 1, SNP = single nucleotide polymorphism.

### 3.3. Comparison of analyzed proteins in plasma and CSF

**Figure 5.**
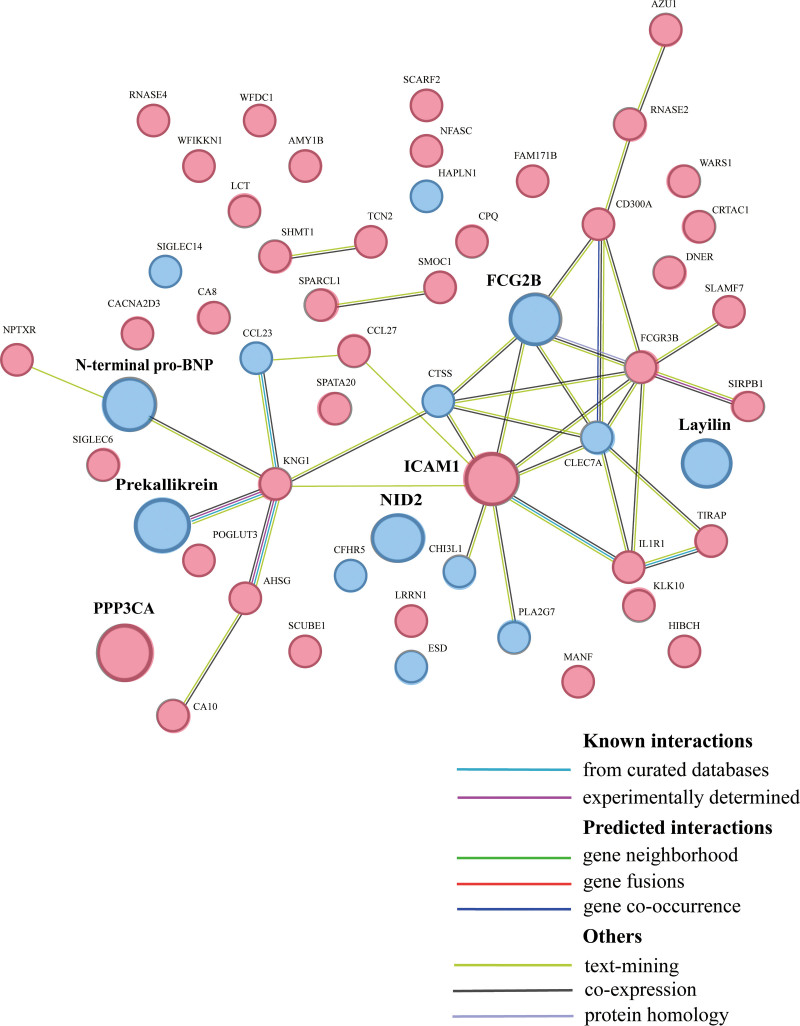
Protein–protein interaction network among the suggestive plasma and CSF causal proteins (primary *P* < .05). Pink solid circles represented plasma proteins; blue solid circles represented CSF proteins. The 7 potential causal proteins were enlarged, including PPP3CA, ICAM-1, FCG2B, N-terminal pro-BNP, NID2, Layilin and Prekallikrein. FCG2B = Fc-gamma-RIIb, ICAM-1 = intercellular adhesion molecular 1, N-terminal pro-BNP = N-terminal pro-B-type natriuretic peptide, NID2 = Nidogen 2, PPP3CA = calcineurin A.

At the protein level, CSF and plasma MR results showed a nonsignificant negative correlation (Spearman correlation coefficient = −0.037, protein number = 63, no *P*-value threshold). Specifically, there was a negative correlation when proteins limiting different *P*-value thresholds were included in the analysis (Fig. S2, Supplemental Digital Content, http://links.lww.com/MD/O356). This suggests that there may be partially overlapping SNPs in CSF and plasma that are associated enough to change the results. Further, PPI network analysis through text mining and co-expression suggested a possible relationship between plasmon-based ICAM-1 and CSF-based FCG2B (Fig. 5).

### 3.4. Association of potential drug targets with current SLE drugs

**Figure 6.**
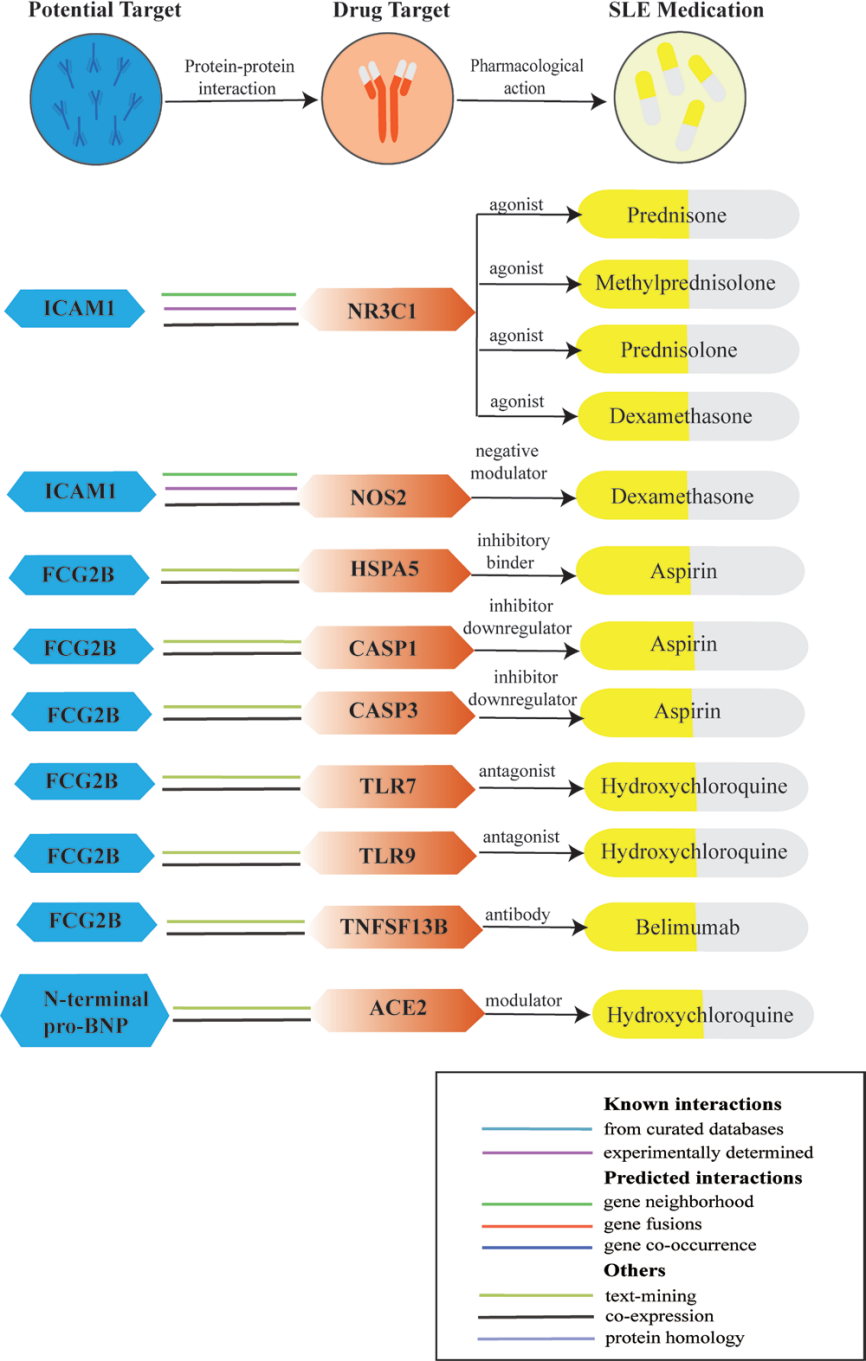
Interaction between identified potential drug targets and current SLE medications targets. FCG2B = Fc-gamma-RIIb, ICAM-1 = intercellular adhesion molecular 1, N-terminal pro-BNP = N-terminal pro-B-type natriuretic peptide, SLE = systemic lupus erythematosus.

The PPI network (Fig. S3, Supplemental Digital Content, http://links.lww.com/MD/O356) revealed interactions between 3 prioritized proteins (FCG2B, ICAM-1, N-terminal pro-BNP) and current SLE drugs targets (Table S5, Supplemental Digital Content, http://links.lww.com/MD/O357, Fig. 6). Using STRING, NR3C1-ICAM-1 (NR3C1, Glucocorticoid Nuclear receptor subfamily 3 group C member 1), NOS2-ICAM-1 (NOS2, Nitric oxide synthase 2), HSPA5-ICAM-1 (HSPA5, Heat Shock Protein A5) were determined to have the most reliable interactions (known interactions). Specifically, ICAM-1 was associated with NR3C1, which are the targets of glucocorticoids such as prednisone, methylprednisolone, prednisolone and dexamethasone. NOS2 is also the target of dexamethasone. ICAM-1 was also associated with HSPA5, which is the target of aspirin. Compare to this, FCG2B was only in text mining and co-expression with CASP3 (the target of aspirin), CASP1 (the target of aspirin), TLR9 (the target of hydroxychloroquine), TNFSF13B (the target of Belimumab) and TLR7 (the target of hydroxychloroquine) (CASP3, Caspase 3; CASP1, Caspase 1; TLR9, Toll-Like Receptor 9; TNFSF13B, TNF superfamily member 13B; TLR7, Toll-Like Receptor 7). N-terminal pro-BNP was in text mining and co-expression with angiotensin-converting enzyme 2, the target of hydroxychloroquine. We also searched Drugbank database for current drugs targeting identified potentially pathogenic proteins. Drugs that may alter the disease have been identified: Hyaluronic acid (Inhibitor binder of ICAM-1), Natalizumab (a ligand of ICAM-1), Etanercept (a ligand of FCG2B) and Alemtuzumab (a binder of FCG2B) (Table S6, Supplemental Digital Content, http://links.lww.com/MD/O357).

### 3.5. External validation of potential drug targets for SLE

We did not seek external validation because the protein in the CSF was the first. Two datasets^[21,22]^ were selected for external validation of plasma proteins. FinnGen dataset and another GWAS dataset^[23]^ were selected for external validation of SLE. CSF FCG2B was successfully replicated in 2 external validations of SLE, and plasma ICAM-1 was successfully replicated in the GWAS dataset^[23]^ (Table S4, Supplemental Digital Content, http://links.lww.com/MD/O357).

## 4. Discussion

To our knowledge, this is the first study combining plasma and CSF proteomics data using “two-sample MR” and “Bayesian co-localization” to explore incidental proteins in SLE. Finally, we identified 3 proteins as potential drug targets for SLE, including CSF FCG2B, CSF N-terminal pro-BNP, and plasma ICAM-1. External validation revealed that the causal relationship between CSF FCG2B and SLE was successfully replicated in the 2 external datasets of SLE, and that plasma ICAM-1 was validated only in the GWAS summary statistics,^[23]^ which further demonstrated the confidence of the potential drug targets identified in this study.

MR analysis uses genetic variation as IVs. In MR research, IVs must meet 3 fundamental assumptions: IVs must be directly associated with exposure factors. IVs are not affected by any potential confounding factors. IVs do not influence the outcomes other than exposure pathways that affect outcomes.^[38]^ Therefore, in search of novel SLE drug targets, we used a comprehensive analysis combining MR And co-localization to evaluate SLE causing proteins as clinical translations of previous GWAS findings.^[39]^ However, it is important to note that the “causation” identified by MR may be reverse causation, horizontal pleiotropy, or genetic confounding of LD.^[39]^ Therefore, bidirectional MR was performed, and the proteins identified by primary MR analysis did not show reverse causality except for the CSF sICAM-1, and steiger filtering further supported this conclusion.^[40]^ About horizontal pleiotropy, only cis-pQTLs were used as tools because they play a direct role in the transcription and/or translation of relevant genes.^[41]^ Bayesian co-localization was also used to rule out bias introduced by LD. Using 0.8 as a critical threshold for posterior probability, the 4 proteins identified by MR (ICAM-1, FCG2B, N-terminal pro-BNP, Layilin) may share the same SLE variant.^[42]^ Two of the 4 identified proteins (ICAM-1 and FCG2B) were found to be associated with other traits by phenotypic scanning, but none of the associations fully explained the relationship between the identified proteins and SLE. For example, we examined plasma ICAM-1 and CSF sICAM-1, which were associated with the lymphote count-associated SNP (rs5498),^[43,44]^ but no significant effect was found. FCG2B was also found to correlate with serum albumin. Only NID2 (rs6572807) was found to be associated with triglyceride measurement,^[45]^ BMI-adjusted waist circumference^[46]^ and triglyceride cholesterol ratio.^[47]^ It is now recognized that SLE has a particular pattern of dyslipoproteinemia characterized by low high-density lipoprotein levels and increased triglycerides, which is aggravated by flares.^[48]^ Hypertriglyceridemia was reported closely associated with SLE.^[49,50]^ Additionally, co-localization analysis didn’t strongly support the NID2 as potential drug target (PPH4 = 0.713 < 0.8) and PPI network failed to demonstrate its interaction with SLE drug targets. Therefore, we did not consider NID2 as potential drug target in this study.

Considering BBB and neuroinflammation as pathophysiologic mechanisms for diffuse manifestations of neuropsychiatric SLE(NPSLE).^[51]^ We investigated the pathogenic proteins of SLE in plasma and CSF and compared their subsequent effects. Notably, when setting no *P*-value threshold, nonsignificant negative correlation was existed between the CSF and plasma MR results (Spearman correlation coefficient = −0.037, number of proteins = 63). However, the negative correlation changed when the number of proteins included in the analysis was restricted with different *P*-value threshold. This suggests that there may be partially overlapping SNPs in CSF and plasma that are associated enough to change the results. About this, we think that the role of the BBB may explain the lack of correlation. BBB was destroyed in the development of SLE, which is the characteristic of NPSLE.^[52]^ It is acknowledged that NPSLE may result from ischemia or penetration of inflammatory mediators and neurotoxic antibodies through the BBB.^[53]^ Hence, the disruption of BBB may explain for the change of correlation between plasma and CSF proteins in MR analysis of SLE. We also examined no bidirectional causality between SLE and the preferred proteins, except for CSF sICAM-1. PPI network analysis suggested that plasmon-based ICAM-1 and CSF-based FCG2B may be related. Nevertheless, these results suggest that CSF and plasma may serve as an important pathway for detecting disease-causing proteins in SLE, and that the identified proteins may be promising drug targets for SLE.

Candidate gene association studies were widely used and found some valuable susceptibility genes such as interleukin-6,^[54]^ Toll-like receptor 2,^[55]^ Vitamin D receptor,^[56]^ cytotoxic T-lymphocyte-associated protein 4,^[57]^ Fc-gamma-RIIa,^[58]^ FCGR2B^[59]^ and Pellino 1.^[60]^ More recently, as a powerful method, GWAS has been able to identify many SLE susceptibility genes and SNPs.^[61–64]^ In view of the unsatisfactory treatment of SLE and the discovery of more and more candidate proteins. It is necessary to discover more protein targets for drugs and promote the development of new drugs. In this study, we first found that plasma ICAM-1 may be a new drug target for treatment of SLE. ICAM-1 mediates cell-cell adhesion and promotes the activation and differentiation of B cells with the help of CD4 T cells.^[65]^ Studies have shown that CD4 T cells with high ICAM-1 expression in patients with moderate to severe SLE could produce less IgG after T-B co-culture.^[65]^ Several systematic evaluations and meta-analyses have shown that elevated ICAM-1 in blood and urine is a biomarker for SLE.^[66,67]^ Serum ICAM-1 levels could identify active lupus nephritis.^[68]^ However, the controversial view holds that serum ICAM-1 level was unable to discriminate between the active and non-active SLE groups.^[69]^ Furthermore, a genetic study in a European population reported that rs3093030 (ICAM-1-ICAM4-ICAM5) has specific association of SLE with Hematologic Disorders.^[70]^ In this study, the causal relationship of ICAM-1 and SLE suggested that elevated plasma ICAM-1 levels may prevent SLE, and external validation supports this conclusion.

The relationship between FCGRs polymorphisms and the risk of SLE has been widely reported. FCGR2B is the only inhibitory Fc receptor and controls many aspects of immune and inflammatory responses.^[71]^ A meta-analysis conducted by Zhu et al evaluated the association between FCGRs polymorphism and the risk of SLE.^[72]^ T They found that the FCGR2B rs1050501 C allele and the FCGR3A rs396991 T allele may be involved in susceptibility and development of SLE. Additionally, in 2004, a study^[73]^ found that rs1050501 was significantly associated with the development of SLE in the Chinese population. Another study also reported that the C allele significantly increased the risk of SLE in Asian populations.^[74]^ Therefore, we believe that FCGR2B rs1050501 is closely associated with SLE, and the C allele is a risk factor for SLE in Asian people. However, contrary to these reported results, CSF FCG2B found in this study may reduce the risk of SLE.

BNP is a biomarker that helps in determining the diagnosis and prognosis of heart failure (HF).^[52]^ A study^[75]^ revealed that SLE patients without cardiac symptoms had higher levels of BNP compared to healthy controls. In addition, BNP levels were higher in patients with active SLE combined with HF.^[75]^ In addition, long-term antimalarial (AM) therapy and elevated creatine phosphokinase could lead to an increased risk of BNP abnormalities, which may be able to predict AM-induced cardiomyopathy.^[76]^ Our analysis suggested that the increased N-terminal pro-BNP was a risk factor for the development of SLE, further supporting these findings.

Additionally, PPI analysis illustrated that FCG2B could interact with ICAM-1. FCG2B was found to interact with current SLE drug targets by PPI analysis and it was the only externally validated biomarker in this study both in the GWAS summary statistics^[23]^ and FinnGen cohort. We hypothesized that it has the strong potential to serve as a drug target for SLE.

However, there are some limitations in our study. First, proteins are derived from different studies, and measurement inconsistencies between studies can lead to bias. Furthermore, the GWAS cycling protein data^[17,19,21,22]^ conducted are all based on aptamers. Second, after rigorous screening conditions, such as genome-wide significant association (*P* < 5 × 10^−8^) and cis-acting pQTL, all prioritized proteins have only 1 cis-acting SNP, which limits the application of other MR methods as well as the lack of heterogeneity tests, and pleiotropy tests. And, our survey of the major found SNPs shows that all SNPs are strong instruments with *F*-statistics >10. Except for PPP3CA; PPP3R1, NID2, Prekallikrein, the proportion of SNP variance explained exceeds 10%. Third, our analysis is based on populations of European descent, limiting the application to other ethnicities. Finally, although we identified some interactions between pathogenic proteins and current drug targets for SLE, the results of the PPI analysis are suggestive rather than conclusive. Further research is needed to confirm these findings.

## 5. Conclusions

In conclusion, our combined analyses suggested that genetically determined plasma PPP3CA; PPP3R1, ICAM-1, cerebrospinal fluid FCG2B, SICAM-1, NID2, N-terminal pro-BNP, Layilin, and Prekallikrein levels are causally associated with SLE risk. Co-localization analyses and PPI network suggest that the identified proteins FCG2B, ICAM-1 and N-terminal pro-BNP may be attractive drug targets for SLE, especially ICAM-1 and FCG2B. Further studies are needed to explore the role of these candidate proteins in SLE.

## Acknowledgments

We express our gratitude to all the genetics consortiums for making the GWAS summary data publicly available. We also express our gratitude to the Core facility and Bioinformatics Laboratory of the Wuhan Union Hospital affiliated with Huazhong University of Science and Technology and the first Hospital of Jilin University for the training and generous sharing of experiences and codes.

## Author contributions

**Conceptualization:** Shiwen Fan, Kaixin Wang, Shuai Wang, Xiangdong Chen.

**Data curation:** Shiwen Fan, Kaixin Wang, Shuai Wang.

**Formal analysis:** Kaixin Wang, Shuai Wang.

**Funding acquisition:** Xiangdong Chen.

**Investigation:** Kaixin Wang.

**Methodology:** Shiwen Fan, Shuai Wang.

**Resources:** Shuai Wang, Xiangdong Chen.

**Software:** Shuai Wang.

**Supervision:** Shuai Wang, Xiangdong Chen.

**Validation:** Shiwen Fan, Kaixin Wang.

**Visualization:** Shiwen Fan, Kaixin Wang.

**Writing – review & editing:** Shiwen Fan, Kaixin Wang, Shuai Wang, Xiangdong Chen.

**Writing – original draft:** Kaixin Wang.

## Supplementary Material


