# Supplementary material for: Potential drug targets for systemic lupus erythematosus identified through Mendelian randomization analysis
Source: Medicine (Baltimore). 2025 Feb 14;104(7):e41439. doi: 10.1097/MD.0000000000041439 (PMC11835111; doi:10.1097/MD.0000000000041439)

Supplementary Fig. 1 Bayesian colocalization analysis of eight potential causal proteins and SLE

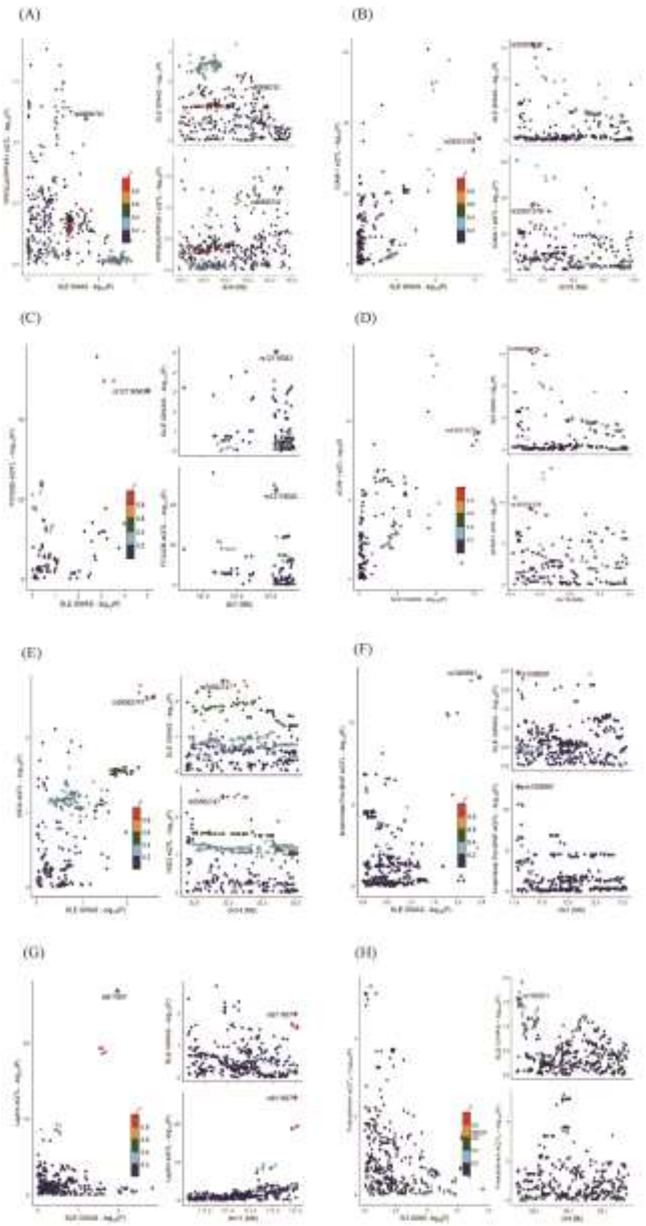

Supplementary Fig. 2 Comparison analysis of MR estimates between plasma proteome and CSF proteome

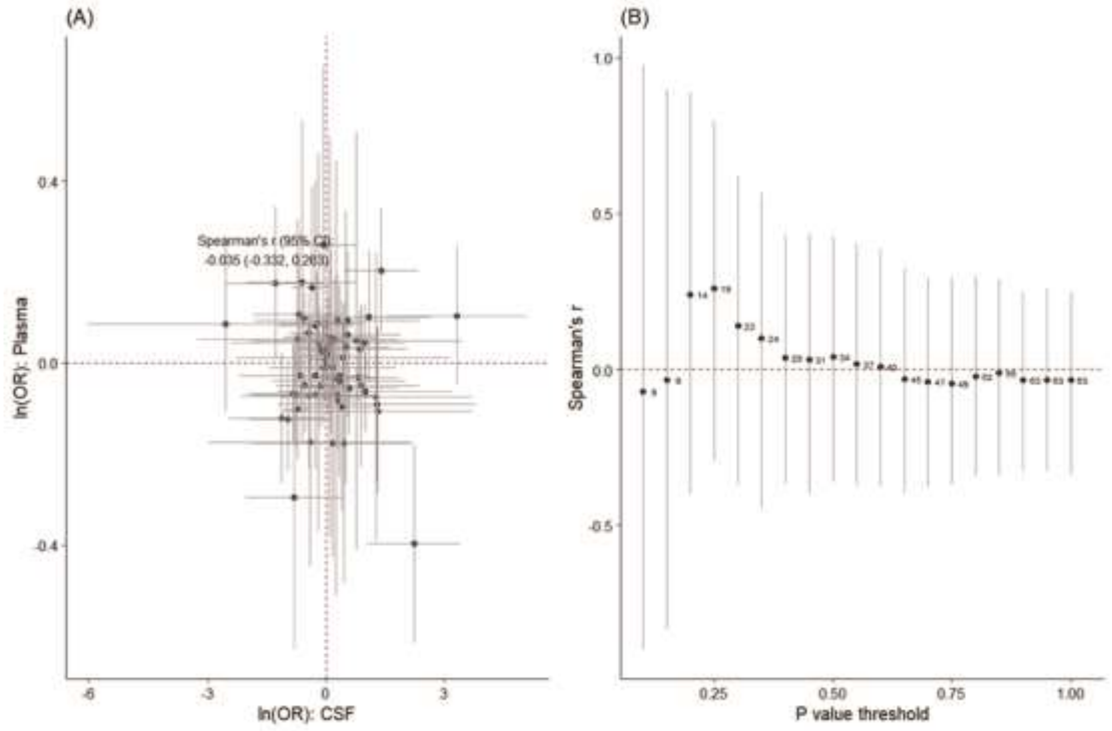

Supplementary Fig.3 Protein-protein interaction network among the candidate causal proteins (PFDR < 0.05) and current SLE medications targets.

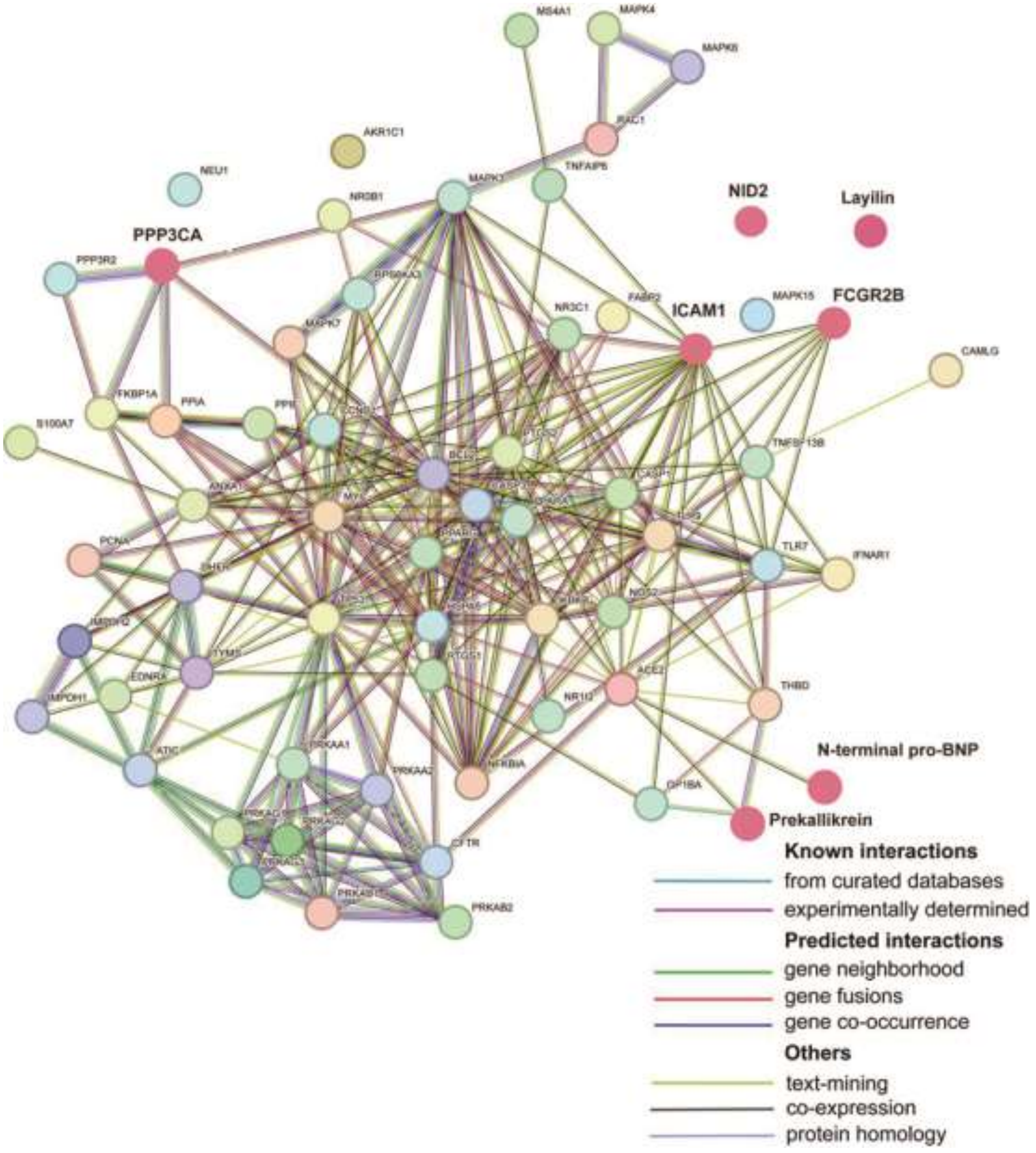

Supplement: Supplementary file 1 [file medi-104-e41439-s001.pdf]
