# Supplementary material for: Potential drug targets for systemic lupus erythematosus identified through Mendelian randomization analysis
Source: Medicine (Baltimore). 2025 Feb 14;104(7):e41439. doi: 10.1097/MD.0000000000041439 (PMC11835111; doi:10.1097/MD.0000000000041439)
Supplement: Supplementary file 2 [file medi-104-e41439-s002.docx]

**Supplementary Table 1 Genetic instruments of plasma and CSF proteins for MR analysis**

| tissue | protein | UniProt | SNP | chr | pos | effect allele | other allele | beta | se | pval | eaf | samplesize | Author | id | F_statistics |
| --- | --- | --- | --- | --- | --- | --- | --- | --- | --- | --- | --- | --- | --- | --- | --- |
| Plasma | ACP1 | A0A140VK37; P24666; Q59EH3 | rs11553746 | 2 | 272203 | T | C | 1.237 | 0.026037 | 1.000E-200 | 0.3247992 | 996 | Suhre | A0A140VK37; P24666; Q59EH3 | 2257.135 |
| Plasma | ACP5 | A0A024R7F8; P13686 | rs79061565 | 19 | 11698659 | G | C | -0.4845 | 0.0364 | 1.600E-40 | 0.122794 | 3301 | Sun | A0A024R7F8; P13686 | 177.168 |
| Plasma | ADA2 | Q9NZK5; B4E3Q4; A0A087X0I3 | rs2231495 | 22 | 17669306 | C | T | -0.8794 | 0.0214 | 1.000E-200 | 0.332516 | 3301 | Sun | Q9NZK5; B4E3Q4; A0A087X0I3 | 1688.672 |
| Plasma | ADAM23 | A0A024R3W8; O75077; E7EWD3 | rs1921673 | 2 | 207324282 | G | A | 0.4321 | 0.0255 | 3.200E-64 | 0.6846 | 3301 | Sun | A0A024R3W8; O75077; E7EWD3 | 287.136 |
| Plasma | ADAMTS13 | Q76LX8 | rs71503194 | 9 | 136298131 | G | T | -0.8719 | 0.0404 | 4.400E-103 | 0.091229 | 3301 | Sun | Q76LX8 | 465.769 |
| Plasma | ADGRE2 | A0JNV7; Q9UHX3 | rs7260110 | 19 | 14501544 | G | A | 0.2969 | 0.04470977 | 5.169E-11 | 0.3599398 | 996 | Suhre | A0JNV7; Q9UHX3 | 44.098 |
| Plasma | ADM | P35318 | rs2923091 | 11 | 10358145 | A | G | -0.204 | 0.025 | 2.280E-16 | 0.662 | 6861 | Yao | P35318 | 66.586 |
| Plasma | AFM | P43652 | rs41265665 | 4 | 74361142 | A | G | -0.8615 | 0.1219415 | 3.033E-12 | 0.03235592 | 989 | Suhre | P43652 | 49.912 |
| Plasma | AGT | B0ZBE2; B2R5S1; P01019 | rs2493151 | 1 | 230878561 | A | G | 0.3349 | 0.04948024 | 2.223E-11 | 0.2349095 | 994 | Suhre | B0ZBE2; B2R5S1; P01019 | 45.811 |
| Plasma | AHSG | P02765 | rs35094235 | 3 | 186328951 | G | T | 0.6252 | 0.0255 | 2.000E-132 | 0.731004 | 3301 | Sun | P02765 | 601.115 |
| Plasma | AKR1A1 | P14550; V9HWI0 | rs72688441 | 1 | 46051053 | A | G | -1.18 | 0.0493 | 1.200E-126 | 0.058959 | 3301 | Sun | P14550; V9HWI0 | 572.889 |
| Plasma | AKR1B1 | A0A024R7A8; P15121 | rs2229542 | 7 | 134135621 | C | T | -0.8187 | 0.1045 | 4.700E-15 | 0.014337 | 3301 | Sun | A0A024R7A8; P15121 | 61.379 |
| Plasma | AKR1C1 | Q04828 | rs145648894 | 10 | 5009739 | G | T | 0.3894 | 0.0393 | 3.800E-23 | 0.111375 | 3301 | Sun | Q04828 | 98.176 |
| Plasma | ALCAM | Q13740; B3KNN9 | rs9830049 | 3 | 105284045 | C | T | -0.306 | 0.0374 | 2.800E-16 | 0.120272 | 3301 | Sun | Q13740; B3KNN9 | 66.942 |
| Plasma | ALDH3A1 | P30838; Q6PKA6; I3L3I9 | rs887241 | 17 | 19645938 | C | A | 0.2421 | 0.0259 | 7.800E-21 | 0.661635 | 3301 | Sun | P30838; Q6PKA6; I3L3I9 | 87.376 |
| Plasma | AMY1A | P04745; Q6NSB3 | rs7538379 | 1 | 104067356 | T | C | -1.0399 | 0.062 | 4.900E-63 | 0.039949 | 3301 | Sun | P04745; Q6NSB3 | 281.319 |
| Plasma | ANG | P03950; W0UV28 | rs17114671 | 14 | 21155270 | C | T | 0.8064 | 0.05956686 | 1.918E-38 | 0.1188566 | 997 | Suhre | P03950; W0UV28 | 183.270 |
| Plasma | ANGPTL1 | A0A024R908; O95841 | rs16853043 | 1 | 178545926 | G | A | 0.4305 | 0.0247 | 5.400E-68 | 0.352388 | 3301 | Sun | A0A024R908; O95841 | 303.775 |
| Plasma | APCS | P02743; V9HWP0 | rs71632673 | 1 | 159536213 | A | C | -0.8369 | 0.082 | 1.900E-24 | 0.022925 | 3301 | Sun | P02743; V9HWP0 | 104.164 |
| Plasma | APMAP | Q9HDC9 | rs8125909 | 20 | 24975835 | C | A | 0.2967 | 0.0363 | 2.800E-16 | 0.132064 | 3301 | Sun | Q9HDC9 | 66.807 |
| Plasma | APOA5 | A0A0B4RUS7; Q6Q788 | rs964184 | 11 | 116648917 | C | G | 0.2625 | 0.0363 | 4.900E-13 | 0.868979 | 3301 | Sun | A0A0B4RUS7; Q6Q788 | 52.293 |
| Plasma | APOB | P04114; Q59HB3; Q7Z7Q0 | rs520354 | 2 | 21259612 | G | A | 0.3033 | 0.04167632 | 6.929E-13 | 0.4733936 | 996 | Suhre | P04114; Q59HB3; Q7Z7Q0 | 52.962 |
| Plasma | APOL1 | O14791; Q2KHQ6; B1AH95 | rs71314970 | 22 | 36638705 | T | C | -0.3659 | 0.0409 | 3.500E-19 | 0.105762 | 3301 | Sun | O14791; Q2KHQ6; B1AH95 | 80.035 |
| Plasma | ARFIP1 | B4E273; P53367; Q8N8M9; B7ZA10 | rs4619875 | 4 | 153701130 | T | C | 0.239 | 0.0248 | 6.600E-22 | 0.403628 | 3301 | Sun | B4E273; P53367; Q8N8M9; B7ZA10 | 92.874 |
| Plasma | ARSB | A0A024RAJ9; P15848; A8K4A0 | rs13159135 | 5 | 78196689 | C | G | -0.214 | 0.0248 | 5.900E-18 | 0.433907 | 3301 | Sun | A0A024RAJ9; P15848; A8K4A0 | 74.460 |
| Plasma | ART3 | Q13508 | rs4859610 | 4 | 77000441 | G | A | -0.3378 | 0.0286 | 3.000E-32 | 0.774293 | 3301 | Sun | Q13508 | 139.504 |
| Plasma | ART4 | Q93070 | rs1001096 | 12 | 14988455 | A | G | 0.74 | 0.0217 | 1.000E-200 | 0.397358 | 3301 | Sun | Q93070 | 1162.904 |
| Plasma | ASAH2; ASAH2B | Q9NR71; P0C7U1 | rs10740617 | 10 | 52027609 | C | A | 0.6665 | 0.0281 | 2.000E-124 | 0.793543 | 3301 | Sun | Q9NR71; P0C7U1 | 562.584 |
| Plasma | ASPH | Q12797; B7ZM96; A0A0A0MSK8; B4DQ07 | rs112760834 | 8 | 62540134 | T | G | -0.5784 | 0.0688 | 4.100E-17 | 0.033209 | 3301 | Sun | Q12797; B7ZM96; A0A0A0MSK8; B4DQ07 | 70.677 |
| Plasma | ASPN | Q9BXN1; Q6P528 | rs2516568 | 9 | 95187380 | T | A | -0.4724 | 0.025 | 2.300E-79 | 0.323246 | 3301 | Sun | Q9BXN1; Q6P528 | 357.059 |
| Plasma | ATP1B2 | P14415 | rs1642762 | 17 | 7554772 | T | C | -0.2881 | 0.0255 | 1.500E-29 | 0.586879 | 3301 | Sun | P14415 | 127.646 |
| Plasma | B3GAT3 | Q5U676; G3V150; O94766 | rs12794886 | 11 | 62383715 | C | G | 0.4422 | 0.0264 | 8.300E-63 | 0.707032 | 3301 | Sun | Q5U676; G3V150; O94766 | 280.563 |
| Plasma | B4GALT1 | P15291; W6MEN3 | rs7019909 | 9 | 33113322 | T | C | 0.4478 | 0.0402 | 7.900E-29 | 0.102573 | 3301 | Sun | P15291; W6MEN3 | 124.084 |
| Plasma | B4GALT2 | O60909 | rs2286241 | 1 | 44440769 | C | G | -0.673 | 0.052 | 3.000E-38 | 0.056172 | 3301 | Sun | O60909 | 167.503 |
| Plasma | BPI | P17213 | rs1780617 | 20 | 36974157 | G | A | -0.6444 | 0.0371 | 1.100E-67 | 0.12241 | 3301 | Sun | P17213 | 301.692 |
| Plasma | BPIFB1 | Q8TDL5 | rs2424961 | 20 | 31694060 | T | C | -0.4366 | 0.0245 | 3.900E-71 | 0.578579 | 3301 | Sun | Q8TDL5 | 317.567 |
| Plasma | BST1 | Q10588 | rs73224660 | 4 | 15714762 | A | G | -1.3967 | 0.0239 | 1.000E-200 | 0.158997 | 3301 | Sun | Q10588 | 3415.155 |
| Plasma | C1QC | A0A024RAA7; P02747 | rs78865058 | 1 | 22944209 | A | G | 1.0754 | 0.0603 | 4.300E-71 | 0.041707 | 3301 | Sun | A0A024RAA7; P02747 | 318.057 |
| Plasma | C1QTNF5 | A0A024R3F8; Q9BXJ0 | rs2248863 | 11 | 119207341 | A | G | 0.3261 | 0.0345 | 3.500E-21 | 0.141788 | 3301 | Sun | A0A024R3F8; Q9BXJ0 | 89.344 |
| Plasma | C7 | P10643; Q05CI3 | rs429017 | 5 | 41263091 | A | G | 0.4333 | 0.04951452 | 9.039E-18 | 0.2527919 | 985 | Suhre | P10643; Q05CI3 | 76.579 |
| Plasma | CA10 | Q9NS85 | rs117399000 | 17 | 50213731 | A | G | -0.5939 | 0.0662 | 3.000E-19 | 0.037436 | 3301 | Sun | Q9NS85 | 80.484 |
| Plasma | CA13 | Q8N1Q1 | rs17741049 | 8 | 86198253 | T | C | -0.7441 | 0.07991591 | 7.915E-20 | 0.06820461 | 997 | Suhre | Q8N1Q1 | 86.695 |
| Plasma | CA3 | P07451; V9HWA3 | rs2072696 | 8 | 86351051 | C | G | -0.2169 | 0.0289 | 6.800E-14 | 0.23803 | 3301 | Sun | P07451; V9HWA3 | 56.328 |
| Plasma | CA6 | B4DUH8; P23280 | rs3765963 | 1 | 9034598 | G | A | 0.6766 | 0.0233 | 4.100E-185 | 0.402359 | 3301 | Sun | B4DUH8; P23280 | 843.242 |
| Plasma | CACNA2D3 | Q8IZS8 | rs34084772 | 3 | 54153517 | A | G | -0.2329 | 0.0323 | 5.100E-13 | 0.184066 | 3301 | Sun | Q8IZS8 | 51.992 |
| Plasma | CAPN1; CAPNS1 | B2RDI5; P07384; B4DWH5; P04632; A0A0C4DGQ5 | rs10895987 | 11 | 64904908 | T | C | -0.4591 | 0.05093103 | 1.007E-18 | 0.2256281 | 995 | Suhre | B2RDI5; P07384; B4DWH5; P04632; A0A0C4DGQ5 | 81.255 |
| Plasma | CASP3 | P42574 | rs870825 | 4 | 185588045 | G | A | -0.3955 | 0.05815609 | 1.800E-11 | 0.1567839 | 995 | Suhre | P42574 | 46.249 |
| Plasma | CBLN1 | P23435 | rs10852587 | 16 | 49006458 | A | T | -1.0805 | 0.0327 | 1.000E-200 | 0.143622 | 3301 | Sun | P23435 | 1091.828 |
| Plasma | CBLN4 | Q9NTU7 | rs74447607 | 20 | 54447947 | T | C | -0.2177 | 0.0312 | 3.000E-12 | 0.191752 | 3301 | Sun | Q9NTU7 | 48.686 |
| Plasma | CBR1 | P16152 | rs16993864 | 21 | 37446599 | A | C | -1.2151 | 0.0827 | 6.600E-49 | 0.021978 | 3301 | Sun | P16152 | 215.880 |
| Plasma | CBR3 | O75828; V9HW40 | rs1028997 | 21 | 37532222 | A | G | -0.764 | 0.0222 | 1.000E-200 | 0.375555 | 3301 | Sun | O75828; V9HW40 | 1184.352 |
| Plasma | CCDC126 | A0A024RA08; Q96EE4 | rs227934 | 7 | 23627287 | T | C | -0.2749 | 0.0246 | 6.900E-29 | 0.47897 | 3301 | Sun | A0A024RA08; Q96EE4 | 124.876 |
| Plasma | CCL14 | Q16627 | rs9903158 | 17 | 34312337 | C | T | -1.0813 | 0.0499 | 3.500E-104 | 0.05615 | 3301 | Sun | Q16627 | 469.560 |
| Plasma | CCL15 | A0A0B4J2E2; Q16663 | rs854624 | 17 | 34327923 | T | G | -1.6912 | 0.039 | 1.000E-200 | 0.930513 | 3301 | Sun | A0A0B4J2E2; Q16663 | 1880.445 |
| Plasma | CCL16 | O15467 | rs112689088 | 17 | 34307457 | C | T | -1.5245 | 0.0336 | 1.000E-200 | 0.097531 | 3301 | Sun | O15467 | 2058.621 |
| Plasma | CCL18 | P55774 | rs9904601 | 17 | 34372006 | A | G | 0.8634 | 0.05955662 | 2.482E-43 | 0.1169679 | 996 | Suhre | P55774 | 210.167 |
| Plasma | CCL23 | P55773 | rs712048 | 17 | 34326215 | C | A | 0.7289 | 0.0353 | 1.600E-94 | 0.873722 | 3301 | Sun | P55773 | 426.370 |
| Plasma | CCL25 | O15444 | rs74959615 | 19 | 8121096 | A | G | -0.9718 | 0.0428 | 5.200E-114 | 0.078294 | 3301 | Sun | O15444 | 515.545 |
| Plasma | CCL3L1 | P16619 | rs2015086 | 17 | 34391617 | G | A | 0.496 | 0.0364 | 3.100E-42 | 0.131452 | 3301 | Sun | P16619 | 185.678 |
| Plasma | CCL4 | P13236 | rs6607368 | 17 | 34819136 | A | C | 0.51 | 0.04410386 | 6.300E-31 | 0.8 | 3394 | Folkersen | P13236 | 133.717 |
| Plasma | CCL5 | D0EI67; P13501 | rs4239252 | 17 | 34163565 | A | G | -0.3458 | 0.05258101 | 7.825E-11 | 0.2113454 | 996 | Suhre | D0EI67; P13501 | 43.251 |
| Plasma | CCL8 | P80075 | rs3138036 | 17 | 32647544 | G | A | -0.4704 | 0.06013228 | 1.326E-14 | 0.1454363 | 997 | Suhre | P80075 | 61.195 |
| Plasma | CCNH | P51946 | rs2230641 | 5 | 86695274 | G | A | -0.2386 | 0.0309 | 1.200E-14 | 0.205375 | 3301 | Sun | P51946 | 59.624 |
| Plasma | CD14 | P08571 | rs3138074 | 5 | 140015932 | A | T | 0.332 | 0.022 | 9.220E-53 | 0.777 | 6861 | Yao | P08571 | 227.736 |
| Plasma | CD177 | A0A087WVM2; Q8N6Q3 | rs73554000 | 19 | 43825494 | C | G | 1.8032 | 0.0787 | 4.400E-116 | 0.023826 | 3301 | Sun | A0A087WVM2; Q8N6Q3 | 524.975 |
| Plasma | CD200R1 | Q8TD46 | rs6791672 | 3 | 112591392 | A | G | -0.1759 | 0.0252 | 2.800E-12 | 0.590611 | 3301 | Sun | Q8TD46 | 48.723 |
| Plasma | CD300C | Q08708 | rs62087214 | 17 | 72467626 | G | A | -0.6787 | 0.0637 | 1.700E-26 | 0.038512 | 3301 | Sun | Q08708 | 113.521 |
| Plasma | CD33 | P20138; Q546G0 | rs12459419 | 19 | 51728477 | T | C | -0.9436 | 0.0209 | 1.000E-200 | 0.329168 | 3301 | Sun | P20138; Q546G0 | 2038.371 |
| Plasma | CD48 | A0A087X1S7; P09326 | rs12124234 | 1 | 160675269 | C | G | 0.2639 | 0.0252 | 1.100E-25 | 0.405154 | 3301 | Sun | A0A087X1S7; P09326 | 109.667 |
| Plasma | CD55 | P08174; B1AP13 | rs11580387 | 1 | 207418408 | G | A | -0.5211 | 0.0279 | 4.700E-78 | 0.233948 | 3301 | Sun | P08174; B1AP13 | 348.846 |
| Plasma | CD59 | P13987; Q6FHM9 | rs2273121 | 11 | 33757770 | A | G | -0.362 | 0.027 | 4.700E-41 | 0.251594 | 3301 | Sun | P13987; Q6FHM9 | 179.759 |
| Plasma | CD5L | O43866 | rs2765501 | 1 | 157804648 | G | A | -0.285 | 0.018 | 4.760E-54 | 0.608 | 6861 | Yao | O43866 | 250.694 |
| Plasma | CDNF | Q49AH0 | rs61738953 | 10 | 14862082 | G | C | -0.6293 | 0.0681 | 2.500E-20 | 0.041783 | 3301 | Sun | Q49AH0 | 85.393 |
| Plasma | CDON | Q4KMG0 | rs3740909 | 11 | 125889526 | T | C | -0.8286 | 0.07791539 | 4.415E-25 | 0.07301108 | 993 | Suhre | Q4KMG0 | 113.095 |
| Plasma | CEL | B4DSX9; O75612; Q86SR3; X6R868 | rs8193016 | 9 | 135917744 | T | C | 0.6854 | 0.0681 | 8.300E-24 | 0.034777 | 3301 | Sun | B4DSX9; O75612; Q86SR3; X6R868 | 101.296 |
| Plasma | CFH | A0A024R962; P08603; A0A0D9SG88 | rs2274700 | 1 | 196682947 | A | G | 0.3665 | 0.0243 | 1.600E-51 | 0.398961 | 3301 | Sun | A0A024R962; P08603; A0A0D9SG88 | 227.476 |
| Plasma | CFHR5 | Q9BXR6 | rs35662416 | 1 | 196967354 | A | G | -1.2002 | 0.0682 | 3.000E-69 | 0.030392 | 3301 | Sun | Q9BXR6 | 309.698 |
| Plasma | CFI | A8K3L0; P05156; B4DRF2; Q8WW88; G3XAM2 | rs7439493 | 4 | 110656730 | A | G | 0.3116 | 0.0246 | 8.900E-37 | 0.415959 | 3301 | Sun | A8K3L0; P05156; B4DRF2; Q8WW88; G3XAM2 | 160.444 |
| Plasma | CHIT1 | Q13231 | rs872583 | 1 | 203184766 | C | T | -1.0982 | 0.0247 | 1.000E-200 | 0.20154 | 3301 | Sun | Q13231 | 1976.828 |
| Plasma | CHL1 | O00533; A0A087X0M8 | rs1015456 | 3 | 107776 | C | T | -0.2082 | 0.0255 | 3.200E-16 | 0.391779 | 3301 | Sun | O00533; A0A087X0M8 | 66.662 |
| Plasma | CHRDL2 | Q6WN34 | rs11607100 | 11 | 74414919 | T | C | -0.6419 | 0.0609 | 5.800E-26 | 0.041669 | 3301 | Sun | Q6WN34 | 111.096 |
| Plasma | CHST9 | Q7L1S5; A0A024RC28 | rs9952639 | 18 | 24709604 | C | G | 0.3331 | 0.0263 | 7.200E-37 | 0.31788 | 3301 | Sun | Q7L1S5; A0A024RC28 | 160.412 |
| Plasma | CKM | B2R892; P06732 | rs11559024 | 19 | 45821183 | C | T | -0.6788 | 0.0844 | 9.100E-16 | 0.021509 | 3301 | Sun | B2R892; P06732 | 64.684 |
| Plasma | CLEC12A | Q5QGZ9 | rs2961544 | 12 | 10136672 | A | G | -1.2541 | 0.0132 | 1.000E-200 | 0.639327 | 3301 | Sun | Q5QGZ9 | 9026.439 |
| Plasma | CLEC3B | E9PHK0; A0A024R2Q7; P05452 | rs2056320 | 3 | 45092415 | G | A | 0.213 | 0.209 | 1.380E-23 | 0.763 | 6861 | Yao | E9PHK0; A0A024R2Q7; P05452 | 1.039 |
| Plasma | CLIC5 | Q53G01; Q9NZA1; Q49AE1 | rs35822882 | 6 | 45916999 | T | G | -0.7339 | 0.0684 | 7.400E-27 | 0.033649 | 3301 | Sun | Q53G01; Q9NZA1; Q49AE1 | 115.123 |
| Plasma | CLMP | B4E3S3; Q9H6B4 | rs35483681 | 11 | 123045730 | T | C | 0.2494 | 0.0246 | 4.500E-24 | 0.553649 | 3301 | Sun | B4E3S3; Q9H6B4 | 102.783 |
| Plasma | CLPS | A0A087WZW1; A0A087X0Q7; P04118 | rs9380534 | 6 | 35751572 | A | G | 0.4476 | 0.0251 | 3.500E-71 | 0.602276 | 3301 | Sun | A0A087WZW1; A0A087X0Q7; P04118 | 318.004 |
| Plasma | CNDP1 | Q96KN2 | rs17817077 | 18 | 72209543 | A | G | 0.35 | 0.04397973 | 4.720E-15 | 0.4055276 | 995 | Suhre | Q96KN2 | 63.333 |
| Plasma | CNTFR | P26992 | rs10972159 | 9 | 34593086 | A | G | -0.6996 | 0.0941 | 1.000E-13 | 0.017903 | 3301 | Sun | P26992 | 55.274 |
| Plasma | CNTN1 | Q12860; A0A024R104 | rs1838343 | 12 | 41196230 | C | T | 0.207 | 0.021 | 6.450E-24 | 0.58 | 6861 | Yao | Q12860; A0A024R104 | 97.163 |
| Plasma | CNTN2 | A0A024R9B4; Q02246; A1L3A3 | rs2071533 | 1 | 205012198 | G | T | -0.7705 | 0.0349 | 6.900E-108 | 0.865176 | 3301 | Sun | A0A024R9B4; Q02246; A1L3A3 | 487.410 |
| Plasma | CNTN4 | A0A024R2E5; Q8IWV2 | rs163352 | 3 | 3098041 | C | G | -0.3249 | 0.0319 | 2.700E-24 | 0.81339 | 3301 | Sun | A0A024R2E5; Q8IWV2 | 103.733 |
| Plasma | CNTN5 | O94779 | rs1461672 | 11 | 99175378 | T | C | 0.734 | 0.08230642 | 2.241E-18 | 0.06130653 | 995 | Suhre | O94779 | 79.529 |
| Plasma | CNTNAP2 | A0A090N7T7; B2RCH4; Q9UHC6 | rs10274393 | 7 | 145378588 | C | G | -0.4332 | 0.0264 | 1.100E-60 | 0.711014 | 3301 | Sun | A0A090N7T7; B2RCH4; Q9UHC6 | 269.258 |
| Plasma | COCH | O43405 | rs34907608 | 14 | 31330413 | A | G | 0.4759 | 0.0366 | 1.000E-38 | 0.118636 | 3301 | Sun | O43405 | 169.071 |
| Plasma | COL15A1 | B3KTP7; P39059 | rs41305481 | 9 | 101767385 | G | A | 0.2437 | 0.027 | 1.900E-19 | 0.301866 | 3301 | Sun | B3KTP7; P39059 | 81.467 |
| Plasma | COL18A1 | D3DSM5; P39060; D3DSM4 | rs2274809 | 21 | 46906711 | G | A | 0.166 | 0.022 | 1.970E-14 | 0.645 | 6861 | Yao | D3DSM5; P39060; D3DSM4 | 56.934 |
| Plasma | COL6A1 | P12109 | rs434206 | 21 | 47366802 | T | G | -0.2117 | 0.0253 | 5.200E-17 | 0.59389 | 3301 | Sun | P12109 | 70.017 |
| Plasma | COLEC12 | Q5KU26 | rs2846667 | 18 | 466810 | G | T | 0.202 | 0.0296 | 9.300E-12 | 0.745476 | 3301 | Sun | Q5KU26 | 46.571 |
| Plasma | CP | A5PL27; P00450 | rs34004251 | 3 | 148929951 | T | A | -0.263 | 0.025 | 4.910E-25 | 0.812 | 6861 | Yao | A5PL27; P00450 | 110.670 |
| Plasma | CPA4 | Q9UI42; A4D1M3 | rs34587586 | 7 | 129938598 | T | G | -1.1658 | 0.0154 | 1.000E-200 | 0.392741 | 3301 | Sun | Q9UI42; A4D1M3 | 5730.687 |
| Plasma | CPB1 | P15086 | rs13318853 | 3 | 148562399 | A | G | 0.2563 | 0.0293 | 2.000E-18 | 0.23152 | 3301 | Sun | P15086 | 76.518 |
| Plasma | CPB2 | A0A087WSY5; Q96IY4 | rs3742264 | 13 | 46648094 | T | C | 0.849 | 0.03982742 | 2.806E-83 | 0.320603 | 995 | Suhre | A0A087WSY5; Q96IY4 | 454.413 |
| Plasma | CPM | P14384 | rs1908671 | 12 | 69433404 | C | G | -0.1859 | 0.0272 | 8.100E-12 | 0.292111 | 3301 | Sun | P14384 | 46.711 |
| Plasma | CPNE1 | B0QZ18; Q99829 | rs12481228 | 20 | 34218673 | C | G | -0.977 | 0.0379 | 1.800E-146 | 0.097658 | 3301 | Sun | B0QZ18; Q99829 | 664.524 |
| Plasma | CPXM1 | Q96SM3 | rs67159741 | 20 | 2780762 | G | GCGCGCGCGTGCACTGTGTGTGCGCGCA | -0.6434 | 0.0402 | 1.000E-57 | 0.100249 | 3301 | Sun | Q96SM3 | 256.159 |
| Plasma | CPZ | Q66K79 | rs2631738 | 4 | 8479754 | G | A | 0.1949 | 0.0243 | 1.000E-15 | 0.519366 | 3301 | Sun | Q66K79 | 64.330 |
| Plasma | CREB3L4 | Q8TEY5 | rs4845586 | 1 | 153942597 | G | T | 0.2556 | 0.0248 | 7.100E-25 | 0.46994 | 3301 | Sun | Q8TEY5 | 106.223 |
| Plasma | CREG1 | O75629 | rs7513428 | 1 | 167515272 | C | T | -0.3111 | 0.0334 | 1.400E-20 | 0.836173 | 3301 | Sun | O75629 | 86.758 |
| Plasma | CRELD1 | Q96HD1; A0A024R2G1 | rs7627326 | 3 | 9981734 | T | G | -1.0936 | 0.0217 | 1.000E-200 | 0.23988 | 3301 | Sun | Q96HD1; A0A024R2G1 | 2539.788 |
| Plasma | CRISP2 | A0A024RD74; P16562 | rs478328 | 6 | 49720877 | G | A | 0.5695 | 0.0227 | 4.200E-139 | 0.454081 | 3301 | Sun | A0A024RD74; P16562 | 629.413 |
| Plasma | CRISPLD2 | A0A140VK80; Q9H0B8 | rs12921670 | 16 | 84838761 | A | G | 0.2615 | 0.0294 | 5.400E-19 | 0.256992 | 3301 | Sun | A0A140VK80; Q9H0B8 | 79.113 |
| Plasma | CRP | P02741 | rs2211320 | 1 | 159693605 | G | A | 0.149 | 0.02 | 4.770E-14 | 0.677 | 6861 | Yao | P02741 | 55.503 |
| Plasma | CSF1 | A0A024R0A1; P09603 | rs17610659 | 1 | 110503296 | T | C | 0.15 | 0.02428048 | 6.500E-10 | 0.48 | 3394 | Folkersen | A0A024R0A1; P09603 | 38.165 |
| Plasma | CSF2RB | P32927; Q6NSJ8 | rs1534881 | 22 | 37329448 | A | G | -0.3158 | 0.0244 | 2.600E-38 | 0.445804 | 3301 | Sun | P32927; Q6NSJ8 | 167.511 |
| Plasma | CSGALNACT2 | A0A0S2Z5K4; Q8N6G5; A0A0S2Z5F9; A0A0S2Z5F5 | rs2435349 | 10 | 43643466 | G | A | -0.1961 | 0.0278 | 1.600E-12 | 0.272811 | 3301 | Sun | A0A0S2Z5K4; Q8N6G5; A0A0S2Z5F9; A0A0S2Z5F5 | 49.758 |
| Plasma | CST1 | P01037 | rs6114248 | 20 | 23711882 | A | G | 0.564 | 0.04255412 | 5.048E-37 | 0.3145729 | 995 | Suhre | P01037 | 175.661 |
| Plasma | CST2 | P09228 | rs6114248 | 20 | 23711882 | A | G | 0.5148 | 0.04288727 | 4.390E-31 | 0.3145729 | 995 | Suhre | P09228 | 144.085 |
| Plasma | CST3 | A0A0K0K1J1; P01034 | rs911119 | 20 | 23612737 | T | C | 0.393 | 0.022 | 6.660E-73 | 0.779 | 6861 | Yao | A0A0K0K1J1; P01034 | 319.110 |
| Plasma | CST5 | P28325 | rs6138152 | 20 | 23850130 | G | A | 0.7176 | 0.05007108 | 1.760E-42 | 0.1955868 | 997 | Suhre | P28325 | 205.396 |
| Plasma | CST6 | Q15828 | rs3825068 | 11 | 65768093 | G | A | 0.5965 | 0.0757 | 3.200E-15 | 0.027795 | 3301 | Sun | Q15828 | 62.091 |
| Plasma | CST7 | O76096 | rs6138458 | 20 | 24973769 | A | G | -0.7626 | 0.04691931 | 7.603E-53 | 0.2271815 | 997 | Suhre | O76096 | 264.174 |
| Plasma | CTRB1 | P17538 | rs8051363 | 16 | 75255217 | G | A | 0.6471 | 0.0242 | 1.600E-157 | 0.704343 | 3301 | Sun | P17538 | 715.010 |
| Plasma | CTSB | Q5HYG5; A0A024R374; P07858; B4DMY4 | rs1692819 | 8 | 11705448 | A | G | 0.4246 | 0.0274 | 5.200E-54 | 0.291825 | 3301 | Sun | Q5HYG5; A0A024R374; P07858; B4DMY4 | 240.137 |
| Plasma | CTSD | P07339; V9HWI3 | rs111693235 | 11 | 1770224 | C | G | 0.35 | 0.03290332 | 2.000E-26 | 0.71 | 3394 | Folkersen | P07339; V9HWI3 | 113.151 |
| Plasma | CTSF | Q9UBX1 | rs1791679 | 11 | 66337874 | A | C | 0.2349 | 0.0269 | 2.500E-18 | 0.289477 | 3301 | Sun | Q9UBX1 | 76.254 |
| Plasma | CTSH | P09668 | rs34593439 | 15 | 79234957 | A | G | -1.147 | 0.0346 | 1.000E-200 | 0.110656 | 3301 | Sun | P09668 | 1098.942 |
| Plasma | CTSS | P25774 | rs41271951 | 1 | 150737220 | G | A | -0.8605 | 0.0419 | 7.100E-94 | 0.08349 | 3301 | Sun | P25774 | 421.768 |
| Plasma | CXCL11 | O14625 | rs10031452 | 4 | 76924933 | C | T | -0.2328 | 0.0245 | 2.400E-21 | 0.528726 | 3301 | Sun | O14625 | 90.289 |
| Plasma | CXCL16 | Q9H2A7 | rs144830084 | 17 | 4618101 | T | A | -0.2245 | 0.0281 | 1.400E-15 | 0.255651 | 3301 | Sun | Q9H2A7 | 63.829 |
| Plasma | CXCL6 | P80162 | rs16850073 | 4 | 74703999 | T | C | 0.8877 | 0.03764047 | 9.660E-98 | 0.3765244 | 984 | Suhre | P80162 | 556.189 |
| Plasma | DCBLD2 | Q96PD2 | rs9864010 | 3 | 98678173 | A | G | -0.6199 | 0.0536 | 6.600E-31 | 0.054612 | 3301 | Sun | Q96PD2 | 133.756 |
| Plasma | DEFB1 | P60022 | rs2738176 | 8 | 6738228 | A | T | -0.3893 | 0.0249 | 5.600E-55 | 0.365039 | 3301 | Sun | P60022 | 244.439 |
| Plasma | DEFB104A | Q8WTQ1 | rs183772362 | 8 | 7243016 | T | C | 0.5043 | 0.0718 | 2.100E-12 | 0.032572 | 3301 | Sun | Q8WTQ1 | 49.332 |
| Plasma | DKK1 | I1W660; O94907 | rs1194673 | 10 | 54141652 | A | G | 0.1824 | 0.0261 | 2.800E-12 | 0.632462 | 3301 | Sun | I1W660; O94907 | 48.839 |
| Plasma | DKK3 | Q9UBP4; F6SYF8 | rs11022114 | 11 | 12038874 | A | G | 0.3364 | 0.0269 | 6.500E-36 | 0.327961 | 3301 | Sun | Q9UBP4; F6SYF8 | 156.389 |
| Plasma | DLK1 | A8K019; P80370; A0A024R6L1 | rs12881760 | 14 | 101176335 | C | G | 0.5356 | 0.0251 | 3.500E-101 | 0.676353 | 3301 | Sun | A8K019; P80370; A0A024R6L1 | 455.338 |
| Plasma | DLL1 | O00548 | rs959025 | 6 | 170588654 | T | C | 0.1911 | 0.0248 | 1.300E-14 | 0.415638 | 3301 | Sun | O00548 | 59.377 |
| Plasma | DNAJC30 | B3KSU4; Q96LL9 | rs73702564 | 7 | 73084816 | T | C | 0.7183 | 0.0622 | 6.900E-31 | 0.038602 | 3301 | Sun | B3KSU4; Q96LL9 | 133.362 |
| Plasma | DPP7 | Q9UHL4 | rs10747049 | 9 | 140008750 | C | G | 0.3641 | 0.0277 | 2.300E-39 | 0.749544 | 3301 | Sun | Q9UHL4 | 172.775 |
| Plasma | DPT | Q07507 | rs1018454 | 1 | 168697761 | C | A | 0.4155 | 0.0241 | 7.800E-67 | 0.585888 | 3301 | Sun | Q07507 | 297.240 |
| Plasma | DSC2 | Q02487 | rs1789063 | 18 | 28673913 | A | T | -0.2159 | 0.0293 | 1.800E-13 | 0.768825 | 3301 | Sun | Q02487 | 54.296 |
| Plasma | DSG2 | Q14126 | rs2704050 | 18 | 29095888 | G | A | -0.1687 | 0.0248 | 9.300E-12 | 0.49311 | 3301 | Sun | Q14126 | 46.273 |
| Plasma | DUSP13 | Q6B8I1; A0A024QZR6; Q9UII6; U3KQ82 | rs6480771 | 10 | 76861680 | C | T | -0.2994 | 0.0247 | 8.300E-34 | 0.415141 | 3301 | Sun | Q6B8I1; A0A024QZR6; Q9UII6; U3KQ82 | 146.930 |
| Plasma | ECM1 | Q16610; A0A140VJI7 | rs13294 | 1 | 150484987 | A | G | -0.8493 | 0.03513585 | 7.727E-102 | 0.3975904 | 996 | Suhre | Q16610; A0A140VJI7 | 584.280 |
| Plasma | EDAR | Q9UNE0 | rs6750059 | 2 | 109611097 | C | T | -0.44 | 0.04900524 | 1.343E-18 | 0.248996 | 996 | Suhre | Q9UNE0 | 80.616 |
| Plasma | EFEMP1 | A0A0S2Z4F1; B2R6M6; Q12805; A0A0S2Z3V1 | rs3791679 | 2 | 56096892 | A | G | -0.226 | 0.021 | 2.490E-26 | 0.761 | 6861 | Yao | A0A0S2Z4F1; B2R6M6; Q12805; A0A0S2Z3V1 | 115.819 |
| Plasma | EGF | P01133 | rs11568972 | 4 | 110889007 | C | A | 0.2654 | 0.0256 | 3.100E-25 | 0.336807 | 3301 | Sun | P01133 | 107.479 |
| Plasma | EMILIN3 | Q9NT22 | rs61739314 | 20 | 39990377 | C | G | -0.6855 | 0.0691 | 3.600E-23 | 0.033292 | 3301 | Sun | Q9NT22 | 98.414 |
| Plasma | ENPP5 | Q9UJA9; B4DHN2 | rs1047153 | 6 | 46128745 | T | C | -0.7891 | 0.0214 | 1.000E-200 | 0.646395 | 3301 | Sun | Q9UJA9; B4DHN2 | 1359.679 |
| Plasma | ENPP7 | Q6UWV6 | rs11871061 | 17 | 77706544 | C | T | 0.9867 | 0.02 | 1.000E-200 | 0.359387 | 3301 | Sun | Q6UWV6 | 2433.942 |
| Plasma | ENTPD1 | P49961 | rs11188501 | 10 | 97600919 | A | G | 0.2147 | 0.0262 | 2.200E-16 | 0.339852 | 3301 | Sun | P49961 | 67.152 |
| Plasma | ENTPD5 | A0A024R6D3; O75356; A0A024R6B4; G3V4I0 | rs57731447 | 14 | 74487521 | A | G | -0.9668 | 0.0489 | 7.800E-87 | 0.05877 | 3301 | Sun | A0A024R6D3; O75356; A0A024R6B4; G3V4I0 | 390.891 |
| Plasma | EPHB2 | B4DSE0; Q4LE53; Q6NVW1; P29323 | rs6687487 | 1 | 23061551 | A | G | -0.6122 | 0.0447 | 1.300E-42 | 0.080316 | 3301 | Sun | B4DSE0; Q4LE53; Q6NVW1; P29323 | 187.574 |
| Plasma | ERAP1 | Q9NZ08 | rs17482078 | 5 | 96118866 | T | C | -0.9615 | 0.0251 | 1.000E-200 | 0.216313 | 3301 | Sun | Q9NZ08 | 1467.409 |
| Plasma | ERAP2 | B2R769; Q6P179 | rs2927608 | 5 | 96252432 | A | G | 1.0523 | 0.0168 | 1.000E-200 | 0.437292 | 3301 | Sun | B2R769; Q6P179 | 3923.382 |
| Plasma | ERLEC1 | Q96DZ1; V9HWD3 | rs58359565 | 2 | 53958919 | A | C | -0.2076 | 0.0295 | 2.100E-12 | 0.218741 | 3301 | Sun | Q96DZ1; V9HWD3 | 49.523 |
| Plasma | ERO1B | Q86YB8 | rs1254194 | 1 | 236399442 | T | G | -0.3822 | 0.0241 | 1.900E-56 | 0.595759 | 3301 | Sun | Q86YB8 | 251.505 |
| Plasma | ESAM | Q96AP7 | rs11219769 | 11 | 124620147 | T | G | -0.2351 | 0.0279 | 3.600E-17 | 0.264058 | 3301 | Sun | Q96AP7 | 71.006 |
| Plasma | ESD | A0A140VJJ2; P10768 | rs8192888 | 13 | 47362384 | C | G | -0.8063 | 0.0404 | 9.800E-89 | 0.093911 | 3301 | Sun | A0A140VJJ2; P10768 | 398.319 |
| Plasma | EVA1C | P58658; B3KWG0 | rs6517101 | 21 | 33868483 | G | T | 0.1792 | 0.026 | 5.900E-12 | 0.331469 | 3301 | Sun | P58658; B3KWG0 | 47.504 |
| Plasma | F10 | P00742; Q5JVE7; Q5JVE8 | rs547138 | 13 | 113792170 | A | T | 0.2581 | 0.0268 | 5.800E-22 | 0.614611 | 3301 | Sun | P00742; Q5JVE7; Q5JVE8 | 92.748 |
| Plasma | F11 | P03951 | rs2289252 | 4 | 187207381 | T | C | 0.4419 | 0.04157342 | 4.600E-25 | 0.4127144 | 991 | Suhre | P03951 | 112.984 |
| Plasma | F7 | P08709; B4DPM2; F5H8B0 | rs776905 | 13 | 113781942 | C | A | -1.104 | 0.06030467 | 9.164E-65 | 0.1128385 | 997 | Suhre | P08709; B4DPM2; F5H8B0 | 335.148 |
| Plasma | FABP1 | P07148; Q05CP7; Q6FGL7 | rs2241883 | 2 | 88424066 | C | T | -0.1905 | 0.0272 | 2.400E-12 | 0.30311 | 3301 | Sun | P07148; Q05CP7; Q6FGL7 | 49.051 |
| Plasma | FAH | P16930 | rs11555096 | 15 | 80472526 | T | C | -1.8824 | 0.0803 | 1.900E-121 | 0.021281 | 3301 | Sun | P16930 | 549.532 |
| Plasma | FAM151A | Q8WW52 | rs11206397 | 1 | 55097068 | T | A | 0.2651 | 0.0261 | 2.600E-24 | 0.326689 | 3301 | Sun | Q8WW52 | 103.166 |
| Plasma | FAM171B | Q6P995 | rs10931256 | 2 | 187685195 | C | T | -0.3336 | 0.0295 | 1.100E-29 | 0.216165 | 3301 | Sun | Q6P995 | 127.882 |
| Plasma | FAM20A | B7Z4Y3; Q8IYA5; Q96MK3; L8B8N7 | rs929477 | 17 | 66655816 | A | G | -0.3717 | 0.0417 | 5.100E-19 | 0.093705 | 3301 | Sun | B7Z4Y3; Q8IYA5; Q96MK3; L8B8N7 | 79.453 |
| Plasma | FAM213A | Q9BRX8 | rs10887868 | 10 | 82194264 | A | G | -0.1879 | 0.025 | 5.500E-14 | 0.412045 | 3301 | Sun | Q9BRX8 | 56.490 |
| Plasma | FAM3B | P58499 | rs73226194 | 21 | 42721869 | T | C | -1.1495 | 0.0614 | 3.900E-78 | 0.039928 | 3301 | Sun | P58499 | 350.495 |
| Plasma | FAM3D | A0A0A8K9B4; Q96BQ1 | rs3749290 | 3 | 58652292 | T | G | -0.481 | 0.0413 | 2.000E-31 | 0.09578 | 3301 | Sun | A0A0A8K9B4; Q96BQ1 | 135.641 |
| Plasma | FCER2 | K3W4U1; P06734 | rs12973524 | 19 | 7758263 | A | G | -0.3062 | 0.0245 | 5.800E-36 | 0.484028 | 3301 | Sun | K3W4U1; P06734 | 156.199 |
| Plasma | FCGR2B | P31994; P31995 | rs6665610 | 1 | 161641384 | A | G | 1.3308 | 0.0224 | 1.000E-200 | 0.204529 | 3301 | Sun | P31994; P31995 | 3529.633 |
| Plasma | FCGR3B | O75015; M9MML6; A0A087WZR4; A0A087WU90 | rs10919543 | 1 | 161508617 | G | A | 0.4353 | 0.0251 | 3.200E-67 | 0.324432 | 3301 | Sun | O75015; M9MML6; A0A087WZR4; A0A087WU90 | 300.767 |
| Plasma | FCN1 | O00602 | rs11103602 | 9 | 137854872 | A | G | 0.5827 | 0.0266 | 1.500E-106 | 0.258023 | 3301 | Sun | O00602 | 479.873 |
| Plasma | FCN2 | Q15485 | rs57136797 | 9 | 137752540 | T | A | -0.6346 | 0.0353 | 3.700E-72 | 0.163996 | 3301 | Sun | Q15485 | 323.185 |
| Plasma | FCRL1 | Q96LA6 | rs4971155 | 1 | 157779182 | A | T | -0.2557 | 0.0243 | 6.300E-26 | 0.506233 | 3301 | Sun | Q96LA6 | 110.726 |
| Plasma | FCRL3 | Q96P31 | rs7528684 | 1 | 157670816 | G | A | 0.5253 | 0.0233 | 1.400E-112 | 0.465109 | 3301 | Sun | Q96P31 | 508.280 |
| Plasma | FCRL4 | Q96PJ5 | rs11582663 | 1 | 157559122 | T | C | -1.1329 | 0.0295 | 1.000E-200 | 0.144281 | 3301 | Sun | Q96PJ5 | 1474.820 |
| Plasma | FCRL6 | Q6DN72 | rs58240276 | 1 | 159783559 | T | C | -0.6094 | 0.0299 | 3.100E-92 | 0.190167 | 3301 | Sun | Q6DN72 | 415.396 |
| Plasma | FETUB | Q9UGM5; E9PG08; Q5J875; B7Z8T3 | rs3733159 | 3 | 186360409 | G | T | 0.2662 | 0.04357813 | 1.433E-09 | 0.3124373 | 997 | Suhre | Q9UGM5; E9PG08; Q5J875; B7Z8T3 | 37.315 |
| Plasma | FGF2 | P09038 | rs308403 | 4 | 123757748 | T | C | -0.5896 | 0.04321437 | 5.796E-39 | 0.3192771 | 996 | Suhre | P09038 | 186.148 |
| Plasma | FLRT2 | O43155 | rs17796777 | 14 | 85806774 | C | A | -0.2353 | 0.0276 | 1.500E-17 | 0.285507 | 3301 | Sun | O43155 | 72.682 |
| Plasma | FLRT3 | Q9NZU0 | rs11908097 | 20 | 14689146 | C | T | 0.4444 | 0.0278 | 2.500E-57 | 0.25127 | 3301 | Sun | Q9NZU0 | 255.540 |
| Plasma | FLT4 | P35916 | rs34221241 | 5 | 180057293 | C | T | -0.363 | 0.0401 | 1.500E-19 | 0.104286 | 3301 | Sun | P35916 | 81.945 |
| Plasma | FN1 | B7ZLE5; P02751; Q6MZM7; Q9UQS6; Q6MZF4; Q6N084 | rs1250258 | 2 | 216300185 | C | T | -0.7113 | 0.04538975 | 1.282E-49 | 0.2545181 | 996 | Suhre | B7ZLE5; P02751; Q6MZM7; Q9UQS6; Q6MZF4; Q6N084 | 245.578 |
| Plasma | FRZB | D9ZGF6; Q92765 | rs288326 | 2 | 183703336 | A | G | 0.5655 | 0.0362 | 3.700E-55 | 0.12625 | 3301 | Sun | D9ZGF6; Q92765 | 244.033 |
| Plasma | FSTL1 | Q12841 | rs1147707 | 3 | 120169248 | T | C | -0.2113 | 0.0255 | 1.100E-16 | 0.389098 | 3301 | Sun | Q12841 | 68.662 |
| Plasma | FUT10 | Q6P4F1 | rs2732317 | 8 | 33330687 | C | A | -0.4501 | 0.0242 | 2.500E-77 | 0.61158 | 3301 | Sun | Q6P4F1 | 345.929 |
| Plasma | FUT3 | A8K737; P21217 | rs708686 | 19 | 5840619 | T | C | -0.853 | 0.0242 | 1.000E-200 | 0.274027 | 3301 | Sun | A8K737; P21217 | 1242.417 |
| Plasma | FUT5 | K7ENC0; Q11128 | rs778809 | 19 | 5830302 | A | G | -0.58 | 0.025 | 1.300E-118 | 0.301394 | 3301 | Sun | K7ENC0; Q11128 | 538.240 |
| Plasma | FUT8 | Q546E0; Q9BYC5; A8K8P8 | rs2127870 | 14 | 65796846 | C | G | -1.049 | 0.0246 | 1.000E-200 | 0.784568 | 3301 | Sun | Q546E0; Q9BYC5; A8K8P8 | 1818.364 |
| Plasma | GALP | Q9UBC7 | rs111265125 | 19 | 56688781 | C | G | 0.6468 | 0.0907 | 1.000E-12 | 0.021819 | 3301 | Sun | Q9UBC7 | 50.854 |
| Plasma | GFRA1 | P56159; B7Z856 | rs10885877 | 10 | 117966090 | G | C | 0.2645 | 0.0272 | 2.000E-22 | 0.295454 | 3301 | Sun | P56159; B7Z856 | 94.561 |
| Plasma | GFRA2 | O00451 | rs15881 | 8 | 21550768 | C | A | 0.2954 | 0.0246 | 4.100E-33 | 0.455607 | 3301 | Sun | O00451 | 144.195 |
| Plasma | GFRAL | Q6UXV0 | rs72975088 | 6 | 55535375 | T | A | 0.2467 | 0.0348 | 1.300E-12 | 0.1485 | 3301 | Sun | Q6UXV0 | 50.255 |
| Plasma | GGH | Q92820 | rs10957266 | 8 | 63923764 | C | T | 0.7985 | 0.0456 | 1.200E-68 | 0.071297 | 3301 | Sun | Q92820 | 306.634 |
| Plasma | GHR | P10912; A0A087X0H5; A0A087X162 | rs150036324 | 5 | 42738222 | C | A | 0.1787 | 0.0263 | 1.000E-11 | 0.594074 | 3301 | Sun | P10912; A0A087X0H5; A0A087X162 | 46.168 |
| Plasma | GLCE | O94923 | rs11854180 | 15 | 69559340 | T | G | 0.8286 | 0.0248 | 1.000E-200 | 0.769146 | 3301 | Sun | O94923 | 1116.314 |
| Plasma | GLRX2 | Q9NS18 | rs148212596 | 1 | 193074511 | G | A | 1.1837 | 0.0881 | 3.500E-41 | 0.019804 | 3301 | Sun | Q9NS18 | 180.523 |
| Plasma | GLTPD2 | A6NH11 | rs34460487 | 17 | 4685228 | A | G | 0.2669 | 0.0259 | 6.800E-25 | 0.346378 | 3301 | Sun | A6NH11 | 106.193 |
| Plasma | GNLY | B4E3H9; P22749 | rs12151621 | 2 | 85934499 | A | C | 0.7736 | 0.0264 | 7.400E-189 | 0.227041 | 3301 | Sun | B4E3H9; P22749 | 858.668 |
| Plasma | GNRH2 | O43555 | rs3787480 | 20 | 3016895 | A | G | -0.2403 | 0.0342 | 2.300E-12 | 0.148627 | 3301 | Sun | O43555 | 49.369 |
| Plasma | GP1BA | L7UYB8; P07359 | rs72835078 | 17 | 4826592 | T | G | 0.3281 | 0.0481 | 9.100E-12 | 0.071969 | 3301 | Sun | L7UYB8; P07359 | 46.529 |
| Plasma | GP5 | P40197 | rs1466733 | 3 | 194120998 | A | G | 0.171 | 0.021 | 8.600E-17 | 0.737 | 6861 | Yao | P40197 | 66.306 |
| Plasma | GP6 | Q9HCN6 | rs1654439 | 19 | 55553647 | T | G | -0.6565 | 0.05487564 | 6.590E-31 | 0.1701807 | 996 | Suhre | Q9HCN6 | 143.123 |
| Plasma | GPC1 | P35052; H7C410 | rs4074478 | 2 | 241446340 | T | C | 0.4237 | 0.0409 | 4.300E-25 | 0.106437 | 3301 | Sun | P35052; H7C410 | 107.317 |
| Plasma | GPC5 | P78333 | rs2147190 | 13 | 92058888 | T | C | 0.3335 | 0.04485649 | 2.267E-13 | 0.3363545 | 993 | Suhre | P78333 | 55.277 |
| Plasma | GPC5 | P78333 | rs342702 | 13 | 92422946 | T | G | -0.8042 | 0.0247 | 1.000E-200 | 0.274008 | 3301 | Sun | P78333 | 1060.069 |
| Plasma | GPNMB | Q14956; A0A024RA55; Q96F58 | rs2268748 | 7 | 23313171 | C | T | 0.6868 | 0.0619 | 1.200E-28 | 0.0406 | 3301 | Sun | Q14956; A0A024RA55; Q96F58 | 123.106 |
| Plasma | GPX7 | Q96SL4 | rs1097234 | 1 | 53063559 | A | C | 0.5633 | 0.0311 | 1.600E-73 | 0.174745 | 3301 | Sun | Q96SL4 | 328.064 |
| Plasma | GRAMD1C | Q8IYS0; B3KUR5 | rs61077924 | 3 | 113625933 | G | C | 0.6198 | 0.0239 | 3.400E-148 | 0.324013 | 3301 | Sun | Q8IYS0; B3KUR5 | 672.523 |
| Plasma | GRN | P28799 | rs5848 | 17 | 42430244 | T | C | -0.2763 | 0.0281 | 8.100E-23 | 0.28735 | 3301 | Sun | P28799 | 96.683 |
| Plasma | GSTA1 | A0A140VJK4; P08263; B7Z1F9 | rs2290758 | 6 | 52662153 | A | G | 0.4157 | 0.0237 | 7.200E-69 | 0.570024 | 3301 | Sun | A0A140VJK4; P08263; B7Z1F9 | 307.655 |
| Plasma | GSTO1 | P78417; V9HWG9 | rs2282326 | 10 | 106020398 | C | A | -0.9108 | 0.0204 | 1.000E-200 | 0.353535 | 3301 | Sun | P78417; V9HWG9 | 1993.360 |
| Plasma | GSTP1 | P09211; V9HWE9 | rs1695 | 11 | 67352689 | G | A | -0.1781 | 0.0254 | 2.300E-12 | 0.34538 | 3301 | Sun | P09211; V9HWE9 | 49.165 |
| Plasma | GZMM | P51124 | rs16989724 | 19 | 531115 | T | C | 0.4303 | 0.0529 | 3.900E-16 | 0.935134 | 3301 | Sun | P51124 | 66.165 |
| Plasma | H6PD | R4GMU1; O95479 | rs34603401 | 1 | 9305445 | C | A | 0.7589 | 0.0317 | 1.000E-126 | 0.150217 | 3301 | Sun | R4GMU1; O95479 | 573.127 |
| Plasma | HAVCR2 | Q8TDQ0 | rs6874178 | 5 | 156530149 | T | A | -0.734 | 0.0286 | 1.000E-145 | 0.817181 | 3301 | Sun | Q8TDQ0 | 658.658 |
| Plasma | HBZ | P02008 | rs2461286 | 16 | 203254 | G | A | -0.8846 | 0.0209 | 1.000E-200 | 0.646861 | 3301 | Sun | P02008 | 1791.436 |
| Plasma | HDHD2 | Q9H0R4; V9HW73 | rs75228657 | 18 | 44741063 | G | A | 0.7021 | 0.0544 | 4.700E-38 | 0.052264 | 3301 | Sun | Q9H0R4; V9HW73 | 166.571 |
| Plasma | HGFAC | D6RAR4; Q04756 | rs1203119 | 4 | 3406952 | A | G | -0.9854 | 0.07592408 | 1.095E-35 | 0.08241206 | 995 | Suhre | D6RAR4; Q04756 | 168.448 |
| Plasma | HP | P00738; Q6PEJ8; A0A0C4DGL8 | rs217184 | 16 | 72105965 | C | T | 0.8688 | 0.0277 | 1.000E-200 | 0.195652 | 3301 | Sun | P00738; Q6PEJ8; A0A0C4DGL8 | 983.739 |
| Plasma | HPGDS | O60760 | rs1965049 | 4 | 95266204 | G | A | 0.4396 | 0.0246 | 1.300E-71 | 0.620956 | 3301 | Sun | O60760 | 319.334 |
| Plasma | HPX | P02790; Q9BS19 | rs7935957 | 11 | 6450200 | A | T | -0.252 | 0.022 | 5.060E-30 | 0.788 | 6861 | Yao | P02790; Q9BS19 | 131.207 |
| Plasma | HS6ST1 | O60243 | rs34827544 | 2 | 129084425 | T | C | -0.3048 | 0.0348 | 1.900E-18 | 0.157293 | 3301 | Sun | O60243 | 76.713 |
| Plasma | HSP90B1 | P14625; V9HWP2 | rs1165693 | 12 | 104340204 | A | G | 1.0974 | 0.0182 | 1.000E-200 | 0.321367 | 3301 | Sun | P14625; V9HWP2 | 3635.692 |
| Plasma | HSPB1 | P04792; V9HW43 | rs13236526 | 7 | 75913642 | A | G | 0.36 | 0.04204151 | 1.100E-17 | 0.7 | 3394 | Folkersen | P04792; V9HW43 | 73.324 |
| Plasma | ICAM1 | P05362 | rs5498 | 19 | 10395683 | G | A | -1.1988 | 0.0136 | 1.000E-200 | 0.430506 | 3301 | Sun | P05362 | 7769.904 |
| Plasma | ICAM5 | Q8N6I2; Q9UMF0 | rs281439 | 19 | 10400110 | C | G | 0.8918 | 0.0258 | 1.000E-200 | 0.7804 | 3301 | Sun | Q8N6I2; Q9UMF0 | 1194.801 |
| Plasma | ICOSLG | O75144; B7Z1W8; A0N0L8 | rs11558819 | 21 | 45656774 | T | C | -0.5762 | 0.0261 | 3.500E-108 | 0.268737 | 3301 | Sun | O75144; B7Z1W8; A0N0L8 | 487.378 |
| Plasma | IDO1 | P14902 | rs7010461 | 8 | 39781444 | T | C | 0.25 | 0.0271 | 2.500E-20 | 0.333882 | 3301 | Sun | P14902 | 85.102 |
| Plasma | IDUA | P35475 | rs3822020 | 4 | 985727 | G | A | 0.608 | 0.0235 | 7.200E-148 | 0.636581 | 3301 | Sun | P35475 | 669.378 |
| Plasma | IFI16 | Q16666 | rs72709516 | 1 | 159004851 | T | C | 0.8518 | 0.0577 | 2.600E-49 | 0.04404 | 3301 | Sun | Q16666 | 217.933 |
| Plasma | IFNAR1 | P17181 | rs2257167 | 21 | 34715699 | C | G | -0.2897 | 0.0363 | 1.400E-15 | 0.13427 | 3301 | Sun | P17181 | 63.692 |
| Plasma | IGF2R | P11717 | rs629849 | 6 | 160494409 | G | A | 0.8992 | 0.0331 | 9.500E-163 | 0.870653 | 3301 | Sun | P11717 | 738.000 |
| Plasma | IGFBP7 | Q16270 | rs1718849 | 4 | 57942323 | C | T | -0.3626 | 0.0281 | 5.200E-38 | 0.766286 | 3301 | Sun | Q16270 | 166.511 |
| Plasma | IGFLR1 | K7EL86; Q9H665 | rs12459634 | 19 | 36230174 | C | T | -0.7007 | 0.033 | 5.800E-100 | 0.144789 | 3301 | Sun | K7EL86; Q9H665 | 450.854 |
| Plasma | IGLL1 | P15814 | rs139571703 | 22 | 23915620 | T | C | -0.7264 | 0.0693 | 9.800E-26 | 0.037383 | 3301 | Sun | P15814 | 109.872 |
| Plasma | IL11RA | Q14626; Q5VZ79 | rs11575578 | 9 | 34656479 | A | G | 0.5065 | 0.0479 | 4.400E-26 | 0.068486 | 3301 | Sun | Q14626; Q5VZ79 | 111.812 |
| Plasma | IL12B | P29460 | rs4921484 | 5 | 158769753 | C | T | 0.3123 | 0.0262 | 7.200E-33 | 0.678009 | 3301 | Sun | P29460 | 142.083 |
| Plasma | IL12RB2 | B4DGA4; Q99665; A0A0A0MTN7; B7ZB60 | rs12566098 | 1 | 67889571 | G | C | 0.2568 | 0.0267 | 6.000E-22 | 0.688055 | 3301 | Sun | B4DGA4; Q99665; A0A0A0MTN7; B7ZB60 | 92.505 |
| Plasma | IL15RA | Q13261; A0A0A0MS77; G8CVM3 | rs8177641 | 10 | 6016892 | G | A | 0.5091 | 0.0251 | 1.700E-91 | 0.317813 | 3301 | Sun | Q13261; A0A0A0MS77; G8CVM3 | 411.395 |
| Plasma | IL16 | Q14005; Q9UME6 | rs4778639 | 15 | 81600451 | G | T | -0.6805 | 0.042 | 4.500E-59 | 0.09142 | 3301 | Sun | Q14005; Q9UME6 | 262.517 |
| Plasma | IL17RA | Q96F46 | rs3827278 | 22 | 17595915 | A | C | 0.975 | 0.04259966 | 1.969E-93 | 0.2316951 | 997 | Suhre | Q96F46 | 523.838 |
| Plasma | IL17RB | Q9NRM6 | rs2232346 | 3 | 53892830 | C | T | 1.1818 | 0.0636 | 3.500E-77 | 0.036587 | 3301 | Sun | Q9NRM6 | 345.282 |
| Plasma | IL17RD | Q8NFM7; B4DXM5 | rs6776722 | 3 | 57142659 | A | G | -0.4623 | 0.0254 | 3.400E-74 | 0.695339 | 3301 | Sun | Q8NFM7; B4DXM5 | 331.269 |
| Plasma | IL18 | A0A024R3E0; Q14116 | rs75649625 | 11 | 112052194 | G | A | 0.29 | 0.03039171 | 1.400E-21 | 0.76 | 3394 | Folkersen | A0A024R3E0; Q14116 | 91.051 |
| Plasma | IL18R1 | Q13478; B7ZKV7 | rs1420106 | 2 | 103035044 | G | A | -0.9085 | 0.0257 | 1.000E-200 | 0.775551 | 3301 | Sun | Q13478; B7ZKV7 | 1249.636 |
| Plasma | IL18RAP | O95256 | rs6543140 | 2 | 103074274 | T | G | 0.4854 | 0.04602363 | 1.013E-24 | 0.3013078 | 994 | Suhre | O95256 | 111.234 |
| Plasma | IL1R2 | P27930 | rs7561460 | 2 | 102617204 | C | T | -0.4025 | 0.0242 | 6.200E-62 | 0.395872 | 3301 | Sun | P27930 | 276.631 |
| Plasma | IL1RAP | A8K6K4; Q9NPH3 | rs6444442 | 3 | 190346060 | G | A | -1.352 | 0.0246 | 1.000E-200 | 0.841013 | 3301 | Sun | A8K6K4; Q9NPH3 | 3020.530 |
| Plasma | IL1RL2 | Q9HB29 | rs2228139 | 2 | 102781649 | G | C | -0.4249 | 0.0484 | 1.600E-18 | 0.069221 | 3301 | Sun | Q9HB29 | 77.070 |
| Plasma | IL1RN | P18510; A0A024R528 | rs6761276 | 2 | 113832312 | C | T | -0.1907 | 0.0248 | 1.500E-14 | 0.577697 | 3301 | Sun | P18510; A0A024R528 | 59.129 |
| Plasma | IL23R | Q5VWK5 | rs11581607 | 1 | 67707690 | A | G | -0.42 | 0.0491 | 1.200E-17 | 0.066948 | 3301 | Sun | Q5VWK5 | 73.170 |
| Plasma | IL27RA | Q6UWB1 | rs35026308 | 19 | 14153293 | C | T | -0.9457 | 0.0286 | 1.000E-200 | 0.176436 | 3301 | Sun | Q6UWB1 | 1093.389 |
| Plasma | IL5RA | A0A024R2C8; Q01344; Q8NHV7 | rs77400868 | 3 | 3150964 | G | A | 0.5096 | 0.0362 | 6.800E-45 | 0.13908 | 3301 | Sun | A0A024R2C8; Q01344; Q8NHV7 | 198.172 |
| Plasma | IL6ST | A0A0A0N0L2; P40189; Q17RA0 | rs11574765 | 5 | 55278967 | G | A | 0.3809 | 0.0388 | 1.000E-22 | 0.115654 | 3301 | Sun | A0A0A0N0L2; P40189; Q17RA0 | 96.374 |
| Plasma | IL7R | P16871 | rs11957503 | 5 | 35883176 | G | T | 0.4248 | 0.04364431 | 1.922E-21 | 0.437249 | 996 | Suhre | P16871 | 94.736 |
| Plasma | ISG15 | P05161 | rs1891906 | 1 | 950243 | C | A | 0.2592 | 0.0253 | 1.400E-24 | 0.385856 | 3301 | Sun | P05161 | 104.961 |
| Plasma | ISLR2 | Q6UXK2 | rs2959011 | 15 | 74611781 | T | A | 0.198 | 0.0261 | 3.200E-14 | 0.34226 | 3301 | Sun | Q6UXK2 | 57.551 |
| Plasma | ITIH5 | G5E9D8; Q86UX2; Q96JW9; C9J2H1; A0A096LP62 | rs7909223 | 10 | 7700709 | G | A | 0.3222 | 0.0286 | 2.000E-29 | 0.747691 | 3301 | Sun | G5E9D8; Q86UX2; Q96JW9; C9J2H1; A0A096LP62 | 126.917 |
| Plasma | KDELC2 | A0A024R3C4; Q7Z4H8 | rs74911261 | 11 | 108357137 | A | G | -1.3136 | 0.0745 | 1.100E-69 | 0.026545 | 3301 | Sun | A0A024R3C4; Q7Z4H8 | 310.895 |
| Plasma | KDR | A0A024RD88; P35968 | rs34231037 | 4 | 55972946 | G | A | -1.1531 | 0.0649 | 1.000E-70 | 0.033769 | 3301 | Sun | A0A024RD88; P35968 | 315.678 |
| Plasma | KIAA1161 | Q6NSJ0 | rs10972076 | 9 | 34356359 | T | C | 0.3082 | 0.0252 | 2.200E-34 | 0.624659 | 3301 | Sun | Q6NSJ0 | 149.577 |
| Plasma | KLK7 | P49862; B4DHX9; A0A024R4H6 | rs2739419 | 19 | 51484562 | G | A | 0.7031 | 0.0408 | 1.100E-66 | 0.908316 | 3301 | Sun | P49862; B4DHX9; A0A024R4H6 | 296.971 |
| Plasma | KLK8 | A0A0A0MQY9; O60259 | rs74705037 | 19 | 51504808 | A | G | 0.9287 | 0.0579 | 6.300E-58 | 0.044509 | 3301 | Sun | A0A0A0MQY9; O60259 | 257.273 |
| Plasma | KNG1 | P01042 | rs2304456 | 3 | 186445052 | G | T | -1.366 | 0.05817837 | 2.888E-97 | 0.1029116 | 996 | Suhre | P01042 | 551.288 |
| Plasma | KYAT3 | B4DW13; Q6YP21 | rs9787133 | 1 | 89382664 | G | C | -0.1716 | 0.0246 | 2.900E-12 | 0.493516 | 3301 | Sun | B4DW13; Q6YP21 | 48.659 |
| Plasma | LAMC2 | Q13753 | rs2276543 | 1 | 183155305 | A | G | 0.6155 | 0.0251 | 4.400E-133 | 0.279009 | 3301 | Sun | Q13753 | 601.324 |
| Plasma | LCT | P09848 | rs4988235 | 2 | 136608646 | A | G | 0.7759 | 0.0244 | 1.000E-200 | 0.711217 | 3301 | Sun | P09848 | 1011.188 |
| Plasma | LEPR | P48357; Q4G138 | rs3790438 | 1 | 66085525 | A | T | -1.3745 | 0.0219 | 1.000E-200 | 0.175255 | 3301 | Sun | P48357; Q4G138 | 3939.139 |
| Plasma | LGALS2 | P05162 | rs5756729 | 22 | 37961353 | T | C | -0.275 | 0.0259 | 2.200E-26 | 0.592511 | 3301 | Sun | P05162 | 112.737 |
| Plasma | LGALS3 | A0A024R693; P17931 | rs9323280 | 14 | 55801687 | A | C | 0.77 | 0.0463489 | 5.600E-62 | 0.87 | 3394 | Folkersen | A0A024R693; P17931 | 275.996 |
| Plasma | LHB | A0A0F7RQE6; P01229 | rs75287599 | 19 | 49517140 | T | C | -0.4342 | 0.0458 | 2.400E-21 | 0.077817 | 3301 | Sun | A0A0F7RQE6; P01229 | 89.877 |
| Plasma | LILRA4 | P59901 | rs2241384 | 19 | 54849942 | A | G | -0.3088 | 0.0327 | 3.400E-21 | 0.170417 | 3301 | Sun | P59901 | 89.178 |
| Plasma | LILRA5 | A6NI73 | rs759819 | 19 | 54815577 | C | T | -0.5429 | 0.0242 | 2.500E-111 | 0.330491 | 3301 | Sun | A6NI73 | 503.279 |
| Plasma | LILRA6 | Q6PI73 | rs35361042 | 19 | 54748737 | G | C | 1.3399 | 0.0376 | 1.000E-200 | 0.091124 | 3301 | Sun | Q6PI73 | 1269.899 |
| Plasma | LILRB1 | A0A087WSV6; Q8NHL6; A0A087WSX8; D9IDM5; A8MVE2; A0A0B4J1W1 | rs2114511 | 19 | 55145093 | C | G | -1.8056 | 0.046 | 1.000E-200 | 0.051234 | 3301 | Sun | A0A087WSV6; Q8NHL6; A0A087WSX8; D9IDM5; A8MVE2; A0A0B4J1W1 | 1540.733 |
| Plasma | LILRB2 | Q8N423 | rs386056 | 19 | 54782919 | T | C | -1.1495 | 0.0233 | 1.000E-200 | 0.201121 | 3301 | Sun | Q8N423 | 2433.919 |
| Plasma | LILRB5 | O75023 | rs12975366 | 19 | 54759361 | C | T | -1.1607 | 0.0152 | 1.000E-200 | 0.405252 | 3301 | Sun | O75023 | 5831.131 |
| Plasma | LIPN | Q5VXI9 | rs10509554 | 10 | 90525792 | T | C | 1.0165 | 0.0213 | 1.000E-200 | 0.306794 | 3301 | Sun | Q5VXI9 | 2277.485 |
| Plasma | LMAN2L | Q9H0V9; B4DI83; B4E308; B4DVH1 | rs2271893 | 2 | 97405440 | A | G | 0.2628 | 0.026 | 4.400E-24 | 0.322912 | 3301 | Sun | Q9H0V9; B4DI83; B4E308; B4DVH1 | 102.165 |
| Plasma | LMNB1 | B4DZT3; P20700 | rs36105360 | 5 | 126161690 | T | C | 0.8039 | 0.0789 | 2.200E-24 | 0.025189 | 3301 | Sun | B4DZT3; P20700 | 103.813 |
| Plasma | LPA | P08519 | rs55730499 | 6 | 161005610 | C | T | -1.255 | 0.046 | 3.770E-167 | 0.939 | 6861 | Yao | P08519 | 744.341 |
| Plasma | LRIG3 | Q6UXM1; C9K080 | rs11172791 | 12 | 59272973 | C | T | -0.4624 | 0.0613 | 4.800E-14 | 0.04525 | 3301 | Sun | Q6UXM1; C9K080 | 56.900 |
| Plasma | LRPAP1 | P30533 | rs78770234 | 4 | 3496683 | A | G | -0.6821 | 0.0688 | 3.600E-23 | 0.032618 | 3301 | Sun | P30533 | 98.292 |
| Plasma | LRRC15 | Q8TF66; B3KWI4 | rs57514363 | 3 | 194087927 | G | T | 0.5927 | 0.0383 | 5.900E-54 | 0.112882 | 3301 | Sun | Q8TF66; B3KWI4 | 239.482 |
| Plasma | LRRN1 | Q6UXK5; A8K6Q2 | rs6801789 | 3 | 3807592 | C | T | 0.246 | 0.0253 | 2.300E-22 | 0.354026 | 3301 | Sun | Q6UXK5; A8K6Q2 | 94.543 |
| Plasma | LY9 | A0A0C4DFU4; Q9HBG7; Q05CA2; Q0VAI0; Q5VYH9 | rs540254 | 1 | 160767737 | C | T | 0.4173 | 0.04480283 | 7.783E-20 | 0.3532663 | 995 | Suhre | A0A0C4DFU4; Q9HBG7; Q05CA2; Q0VAI0; Q5VYH9 | 86.753 |
| Plasma | MAN2B2 | B7Z754; E9PCD7; Q9Y2E5 | rs2301790 | 4 | 6600012 | G | A | 0.3342 | 0.0238 | 1.000E-44 | 0.483846 | 3301 | Sun | B7Z754; E9PCD7; Q9Y2E5 | 197.178 |
| Plasma | MANBA | O00462 | rs227370 | 4 | 103612043 | C | T | -0.5596 | 0.0248 | 7.800E-113 | 0.674618 | 3301 | Sun | O00462 | 509.157 |
| Plasma | MANEA | Q5SRI9 | rs80268500 | 6 | 96009498 | C | T | -1.6814 | 0.0345 | 1.000E-200 | 0.081945 | 3301 | Sun | Q5SRI9 | 2375.220 |
| Plasma | MANSC1 | Q9H8J5 | rs2160588 | 12 | 12487447 | A | G | 0.5024 | 0.0371 | 8.700E-42 | 0.119697 | 3301 | Sun | Q9H8J5 | 183.380 |
| Plasma | MANSC4 | A6NHS7 | rs36138811 | 12 | 27927881 | C | T | 0.6369 | 0.0277 | 7.400E-117 | 0.231771 | 3301 | Sun | A6NHS7 | 528.668 |
| Plasma | MAPK13 | A0A024RD04; O15264 | rs12210904 | 6 | 36098191 | A | C | 0.2538 | 0.0266 | 1.500E-21 | 0.289473 | 3301 | Sun | A0A024RD04; O15264 | 91.037 |
| Plasma | MAPKAPK2 | P49137 | rs6669284 | 1 | 206890435 | A | G | 0.3261 | 0.0443227 | 3.902E-13 | 0.3493976 | 996 | Suhre | P49137 | 54.131 |
| Plasma | MATN4 | A2RRP8; B3KQB2; A5D8U1; O95460; A6NNA4 | rs11697677 | 20 | 43925554 | G | A | -0.2298 | 0.028 | 2.500E-16 | 0.253541 | 3301 | Sun | A2RRP8; B3KQB2; A5D8U1; O95460; A6NNA4 | 67.357 |
| Plasma | MBL2 | P11226 | rs7899547 | 10 | 54536839 | G | T | 0.9477 | 0.0202 | 1.000E-200 | 0.65277 | 3301 | Sun | P11226 | 2201.096 |
| Plasma | MCAM | A0A024R3I5; P43121 | rs11217234 | 11 | 119177938 | A | G | -0.144 | 0.026 | 4.320E-08 | 0.718 | 6861 | Yao | A0A024R3I5; P43121 | 30.675 |
| Plasma | MFAP2 | P55001; A0A024RA94 | rs4920605 | 1 | 17315425 | A | G | 0.2073 | 0.0243 | 1.300E-17 | 0.553544 | 3301 | Sun | P55001; A0A024RA94 | 72.776 |
| Plasma | MFGE8 | Q08431; X6R3G6; B4E396; F5GZN3; B3KTQ2 | rs1961839 | 15 | 89467454 | A | G | -0.2253 | 0.0252 | 3.400E-19 | 0.397423 | 3301 | Sun | Q08431; X6R3G6; B4E396; F5GZN3; B3KTQ2 | 79.932 |
| Plasma | MGAT2 | Q10469 | rs28396798 | 14 | 50075319 | T | C | 0.1949 | 0.0244 | 1.300E-15 | 0.54747 | 3301 | Sun | Q10469 | 63.803 |
| Plasma | MGAT4B | Q9UQ53 | rs73351608 | 5 | 179232064 | T | A | -0.9586 | 0.0852 | 2.200E-29 | 0.021516 | 3301 | Sun | Q9UQ53 | 126.589 |
| Plasma | MGP | A0A024RAX0; P08493 | rs7135211 | 12 | 15052758 | G | A | 0.265 | 0.019 | 3.620E-44 | 0.622 | 6861 | Yao | A0A024RAX0; P08493 | 194.529 |
| Plasma | MIA | A0A024R0P1; Q16674 | rs2604877 | 19 | 41275048 | C | T | 1.4417 | 0.0424 | 1.000E-200 | 0.068031 | 3301 | Sun | A0A024R0P1; Q16674 | 1156.160 |
| Plasma | MMP1 | B4DN15; P03956; Q53G95 | rs471994 | 11 | 102697731 | G | A | 0.32 | 0.02578519 | 2.300E-35 | 0.65 | 3394 | Folkersen | B4DN15; P03956; Q53G95 | 154.014 |
| Plasma | MMP10 | P09238 | rs17860955 | 11 | 102649482 | C | T | -0.8693 | 0.0869 | 1.400E-23 | 0.021509 | 3301 | Sun | P09238 | 100.069 |
| Plasma | MMP12 | P39900 | rs28381684 | 11 | 102737192 | T | A | -0.7789 | 0.0348 | 5.100E-111 | 0.124644 | 3301 | Sun | P39900 | 500.962 |
| Plasma | MMP7 | P09237 | rs11568819 | 11 | 102401633 | A | G | 0.6681 | 0.08764911 | 5.837E-14 | 0.06690141 | 994 | Suhre | P09237 | 58.102 |
| Plasma | MMP8 | B4E0I2; P22894 | rs35231465 | 11 | 102584135 | A | G | -0.583 | 0.061 | 2.260E-21 | 0.023 | 6861 | Yao | B4E0I2; P22894 | 91.343 |
| Plasma | MMP9 | P14780 | rs2250889 | 20 | 44642406 | C | G | -0.6221 | 0.0585 | 2.100E-26 | 0.953378 | 3301 | Sun | P14780 | 113.086 |
| Plasma | MPO | P05164 | rs34097845 | 17 | 56358429 | T | C | -0.5736 | 0.0497 | 8.500E-31 | 0.067304 | 3301 | Sun | P05164 | 133.200 |
| Plasma | MRC2 | Q9UBG0 | rs146385050 | 17 | 60637258 | A | C | -0.2188 | 0.0323 | 1.300E-11 | 0.195049 | 3301 | Sun | Q9UBG0 | 45.887 |
| Plasma | MSMB | P08118 | rs10993994 | 10 | 51549496 | C | T | 0.9821 | 0.0189 | 1.000E-200 | 0.59627 | 3301 | Sun | P08118 | 2700.150 |
| Plasma | MTHFS | P49914 | rs7173566 | 15 | 80211691 | C | T | -0.2126 | 0.0263 | 6.000E-16 | 0.342884 | 3301 | Sun | P49914 | 65.345 |
| Plasma | MTRF1L | Q9UGC7; B4DMX1 | rs503366 | 6 | 153333550 | C | T | 0.1652 | 0.0243 | 1.100E-11 | 0.497366 | 3301 | Sun | Q9UGC7; B4DMX1 | 46.218 |
| Plasma | MXRA7 | P84157; Q6ZR64 | rs9900613 | 17 | 74674857 | T | C | -0.229 | 0.0247 | 1.900E-20 | 0.430519 | 3301 | Sun | P84157; Q6ZR64 | 85.956 |
| Plasma | NAAA | Q02083 | rs9996608 | 4 | 76848231 | T | C | -0.5485 | 0.0257 | 3.900E-101 | 0.300448 | 3301 | Sun | Q02083 | 455.499 |
| Plasma | NAGK | C9JEV6; Q9UJ70 | rs7606102 | 2 | 71276399 | G | A | -0.432 | 0.0331 | 6.000E-39 | 0.170314 | 3301 | Sun | C9JEV6; Q9UJ70 | 170.338 |
| Plasma | NAGPA | Q9UK23 | rs12599777 | 16 | 5079466 | G | A | -0.4178 | 0.0317 | 1.400E-39 | 0.204198 | 3301 | Sun | Q9UK23 | 173.707 |
| Plasma | NCAM1 | P13591; A0A087WWD4 | rs11214489 | 11 | 112975934 | C | T | 0.368 | 0.024 | 3.010E-54 | 0.818 | 6861 | Yao | P13591; A0A087WWD4 | 235.111 |
| Plasma | NELL1 | B3KXR2; J3KNC5; K9UUD5; Q92832; F5H6I3 | rs61652119 | 11 | 20955270 | A | G | 1.1098 | 0.0553 | 1.300E-89 | 0.048674 | 3301 | Sun | B3KXR2; J3KNC5; K9UUD5; Q92832; F5H6I3 | 402.753 |
| Plasma | NEO1 | Q59FP8; Q92859 | rs12903656 | 15 | 73326961 | C | G | 0.2861 | 0.0415 | 5.500E-12 | 0.10075 | 3301 | Sun | Q59FP8; Q92859 | 47.527 |
| Plasma | NFASC | O94856; B4DRH7 | rs6667532 | 1 | 204948659 | G | A | 0.8226 | 0.0387 | 4.100E-100 | 0.10515 | 3301 | Sun | O94856; B4DRH7 | 451.810 |
| Plasma | NID2 | Q14112 | rs1151582 | 14 | 52482768 | T | C | -0.3942 | 0.0233 | 2.600E-64 | 0.456621 | 3301 | Sun | Q14112 | 286.234 |
| Plasma | NMRAL1 | Q9HBL8 | rs11557236 | 16 | 4519439 | A | G | -0.4905 | 0.0443 | 1.500E-28 | 0.082096 | 3301 | Sun | Q9HBL8 | 122.594 |
| Plasma | NOG | Q13253 | rs79084672 | 17 | 54856140 | G | A | 1.2369 | 0.1154 | 8.700E-27 | 0.011867 | 3301 | Sun | Q13253 | 114.883 |
| Plasma | NOV | A0A024R9J4; P48745 | rs58936256 | 8 | 120422799 | C | T | 0.2085 | 0.0301 | 4.200E-12 | 0.215178 | 3301 | Sun | A0A024R9J4; P48745 | 47.982 |
| Plasma | NPPB | P16860 | rs198379 | 1 | 11915467 | T | C | -0.266 | 0.024 | 1.580E-41 | 0.608 | 6861 | Yao | P16860 | 122.840 |
| Plasma | NPTX1 | Q15818 | rs62069681 | 17 | 78624702 | C | T | 0.514 | 0.0417 | 7.600E-35 | 0.09319 | 3301 | Sun | Q15818 | 151.934 |
| Plasma | NPW | Q8N729 | rs35327014 | 16 | 2076202 | G | A | -0.4266 | 0.0288 | 1.600E-49 | 0.231175 | 3301 | Sun | Q8N729 | 219.410 |
| Plasma | NQO1 | P15559; B4DLR8 | rs77944668 | 16 | 69718112 | A | G | -0.7498 | 0.0283 | 1.600E-154 | 0.19326 | 3301 | Sun | P15559; B4DLR8 | 701.969 |
| Plasma | NRP1 | O14786; Q68DN3; A8K9V7; Q59F20; Q6X907; Q6AWA9 | rs2506149 | 10 | 33480713 | T | C | -0.2805 | 0.0256 | 6.900E-28 | 0.358077 | 3301 | Sun | O14786; Q68DN3; A8K9V7; Q59F20; Q6X907; Q6AWA9 | 120.057 |
| Plasma | NRP2 | O60462; Q7LBX6; Q7Z3T9; X5D2Q8; A0A024R412; A0A024R3W6 | rs16837641 | 2 | 206634869 | A | G | 0.2094 | 0.0271 | 1.100E-14 | 0.321865 | 3301 | Sun | O60462; Q7LBX6; Q7Z3T9; X5D2Q8; A0A024R412; A0A024R3W6 | 59.706 |
| Plasma | NT5C | Q8TCD5; V9HWF3 | rs78625720 | 17 | 73140941 | A | G | -0.7529 | 0.0719 | 1.200E-25 | 0.029536 | 3301 | Sun | Q8TCD5; V9HWF3 | 109.652 |
| Plasma | NTN1 | O95631 | rs72809988 | 17 | 8986397 | A | G | -0.5788 | 0.038 | 1.600E-52 | 0.110474 | 3301 | Sun | O95631 | 232.001 |
| Plasma | NTN4 | A8K3H6; B2RE43; Q9HB63 | rs17288108 | 12 | 96131895 | G | A | -0.2779 | 0.0323 | 7.200E-18 | 0.181915 | 3301 | Sun | A8K3H6; B2RE43; Q9HB63 | 74.024 |
| Plasma | NTNG1 | Q5IEC3; Q5IEC8; Q9Y2I2; X5DNW2; B4DKF0 | rs115668827 | 1 | 107678268 | C | G | 1.0036 | 0.0565 | 1.100E-70 | 0.048772 | 3301 | Sun | Q5IEC3; Q5IEC8; Q9Y2I2; X5DNW2; B4DKF0 | 315.518 |
| Plasma | NUDT12 | B4E1W3; E7EM93; Q9BQG2 | rs74692061 | 5 | 102903643 | G | A | -0.3756 | 0.0341 | 2.800E-28 | 0.156975 | 3301 | Sun | B4E1W3; E7EM93; Q9BQG2 | 121.323 |
| Plasma | NUDT9 | Q96KB3; Q9BW91; A0A024RDI6; Q8NG26 | rs28696943 | 4 | 88310135 | G | A | -0.3571 | 0.0323 | 2.300E-28 | 0.166852 | 3301 | Sun | Q96KB3; Q9BW91; A0A024RDI6; Q8NG26 | 122.229 |
| Plasma | OAF | Q86UD1 | rs117554512 | 11 | 120098329 | T | C | 0.6816 | 0.0431 | 2.100E-56 | 0.086262 | 3301 | Sun | Q86UD1 | 250.095 |
| Plasma | OAS1 | P00973; F8VXY3 | rs4767027 | 12 | 113359157 | C | T | -0.2701 | 0.0256 | 6.200E-26 | 0.653837 | 3301 | Sun | P00973; F8VXY3 | 111.319 |
| Plasma | OBP2B | Q9NPH6 | rs4454354 | 9 | 136089529 | C | T | 0.358 | 0.0298 | 2.700E-33 | 0.786126 | 3301 | Sun | Q9NPH6 | 144.322 |
| Plasma | ORM1 | P02763 | rs116994374 | 9 | 117084672 | G | A | -0.409 | 0.054 | 2.550E-14 | 0.94 | 6861 | Yao | P02763 | 57.367 |
| Plasma | OSMR | Q99650 | rs357253 | 5 | 38907422 | T | C | 0.2089 | 0.0292 | 8.300E-13 | 0.231854 | 3301 | Sun | Q99650 | 51.181 |
| Plasma | PAM | P19021; O43832 | rs257309 | 5 | 102418604 | G | A | -0.4664 | 0.0248 | 3.800E-79 | 0.351714 | 3301 | Sun | P19021; O43832 | 353.683 |
| Plasma | PATE4 | P0C8F1 | rs875500 | 11 | 125698654 | A | T | -0.4819 | 0.0262 | 1.000E-75 | 0.719869 | 3301 | Sun | P0C8F1 | 338.307 |
| Plasma | PCOLCE | Q15113 | rs9801017 | 7 | 100236202 | A | G | 0.2113 | 0.0254 | 1.000E-16 | 0.635533 | 3301 | Sun | Q15113 | 69.204 |
| Plasma | PCOLCE2 | Q9UKZ9 | rs34516933 | 3 | 142603861 | A | T | 0.422 | 0.0246 | 5.400E-66 | 0.356379 | 3301 | Sun | Q9UKZ9 | 294.276 |
| Plasma | PCSK1 | P29120 | rs6234 | 5 | 95728974 | C | G | -1.0975 | 0.0209 | 1.000E-200 | 0.260198 | 3301 | Sun | P29120 | 2757.506 |
| Plasma | PDCD1LG2 | Q9BQ51 | rs16923189 | 9 | 5510644 | G | A | 0.3879 | 0.0259 | 7.900E-51 | 0.297639 | 3301 | Sun | Q9BQ51 | 224.306 |
| Plasma | PDCD5 | O14737 | rs4499344 | 19 | 33073431 | A | G | 0.354 | 0.0269 | 1.200E-39 | 0.291128 | 3301 | Sun | O14737 | 173.182 |
| Plasma | PDE5A | O76074; G5E9C5 | rs4834770 | 4 | 120241849 | A | G | -0.314 | 0.04461735 | 3.633E-12 | 0.4341709 | 995 | Suhre | O76074; G5E9C5 | 49.528 |
| Plasma | PDGFD | Q9GZP0 | rs7950273 | 11 | 104031598 | G | C | -0.2749 | 0.0269 | 1.400E-24 | 0.287969 | 3301 | Sun | Q9GZP0 | 104.435 |
| Plasma | PDGFRB | P09619; Q59F04 | rs2304058 | 5 | 149508544 | G | C | 0.9308 | 0.0203 | 1.000E-200 | 0.556673 | 3301 | Sun | P09619; Q59F04 | 2102.426 |
| Plasma | PDIA5 | Q14554 | rs2278668 | 3 | 122835232 | C | T | -0.5216 | 0.0234 | 4.000E-110 | 0.593477 | 3301 | Sun | Q14554 | 496.871 |
| Plasma | PEAR1 | Q5VY43 | rs12137505 | 1 | 156883546 | G | A | -0.2226 | 0.0251 | 8.100E-19 | 0.406496 | 3301 | Sun | Q5VY43 | 78.651 |
| Plasma | PENK | A0A024R7V4; P01210 | rs2670014 | 8 | 57376781 | T | C | -0.4653 | 0.0234 | 1.300E-87 | 0.473963 | 3301 | Sun | A0A024R7V4; P01210 | 395.398 |
| Plasma | PF4V1 | P10720 | rs941758 | 4 | 74718941 | A | C | -0.4937 | 0.0253 | 6.600E-85 | 0.708177 | 3301 | Sun | P10720 | 380.790 |
| Plasma | PGLYRP1 | O75594 | rs8102493 | 19 | 46530389 | C | T | -0.2168 | 0.0259 | 5.100E-17 | 0.333381 | 3301 | Sun | O75594 | 70.068 |
| Plasma | PGM1 | B7Z6C2; P36871; B4DDQ8 | rs1126728 | 1 | 64097432 | T | C | 0.2869 | 0.0289 | 2.800E-23 | 0.227841 | 3301 | Sun | B7Z6C2; P36871; B4DDQ8 | 98.552 |
| Plasma | PI3 | P19957 | rs16989763 | 20 | 43779963 | C | T | 0.3756 | 0.0302 | 1.600E-35 | 0.195049 | 3301 | Sun | P19957 | 154.681 |
| Plasma | PIANP | Q8IYJ0 | rs11064321 | 12 | 6809896 | C | G | 0.2262 | 0.027 | 5.000E-17 | 0.397321 | 3301 | Sun | Q8IYJ0 | 70.187 |
| Plasma | PIGR | P01833 | rs2007272 | 1 | 207113755 | C | G | -0.205 | 0.0248 | 1.400E-16 | 0.425661 | 3301 | Sun | P01833 | 68.329 |
| Plasma | PLA2G2A | A0A024RA96; P14555 | rs11573156 | 1 | 20306146 | C | G | 1.0205 | 0.0243 | 1.000E-200 | 0.231634 | 3301 | Sun | A0A024RA96; P14555 | 1763.654 |
| Plasma | PLA2R1 | Q13018; B7ZML4 | rs3749117 | 2 | 160885442 | C | T | -0.9021 | 0.0188 | 1.000E-200 | 0.501272 | 3301 | Sun | Q13018; B7ZML4 | 2302.468 |
| Plasma | PLAU | P00749; Q59GZ8; B4DNJ4; A0A024QZM9 | rs2227551 | 10 | 75669190 | T | G | -0.2435 | 0.0266 | 5.400E-20 | 0.716116 | 3301 | Sun | P00749; Q59GZ8; B4DNJ4; A0A024QZM9 | 83.798 |
| Plasma | PLEKHA7 | E9PKC0; Q6IQ23 | rs382280 | 11 | 16857799 | T | C | 0.2767 | 0.04 | 4.800E-12 | 0.121245 | 3301 | Sun | E9PKC0; Q6IQ23 | 47.852 |
| Plasma | PLG | P00747; Q5TEH5 | rs783150 | 6 | 161226939 | T | C | -0.5174 | 0.06566893 | 8.700E-15 | 0.1135678 | 995 | Suhre | P00747; Q5TEH5 | 62.077 |
| Plasma | PLXNB2 | O15031 | rs28573806 | 22 | 50727792 | C | T | 0.5315 | 0.0232 | 3.500E-116 | 0.399591 | 3301 | Sun | O15031 | 524.844 |
| Plasma | PLXNC1 | O60486 | rs115651556 | 12 | 94613898 | A | G | -2.1447 | 0.1008 | 2.100E-100 | 0.013723 | 3301 | Sun | O60486 | 452.702 |
| Plasma | PLXNC1 | O60486 | rs7972001 | 12 | 94623502 | T | C | -1.005 | 0.122632 | 7.773E-16 | 0.03373615 | 993 | Suhre | O60486 | 67.162 |
| Plasma | POFUT1 | Q9H488 | rs76143353 | 20 | 30815755 | T | C | -0.8 | 0.0499 | 9.300E-58 | 0.059564 | 3301 | Sun | Q9H488 | 257.027 |
| Plasma | POGLUT1 | Q8NBL1; B4DJ97 | rs75203710 | 3 | 119121679 | C | T | 0.3935 | 0.0483 | 3.800E-16 | 0.070387 | 3301 | Sun | Q8NBL1; B4DJ97 | 66.374 |
| Plasma | POMGNT2 | A0A024R2P4; Q8NAT1 | rs729654 | 3 | 43147652 | T | C | -0.2561 | 0.0239 | 9.300E-27 | 0.473213 | 3301 | Sun | A0A024R2P4; Q8NAT1 | 114.822 |
| Plasma | PPA1 | Q15181; V9HWB5 | rs10823500 | 10 | 72005190 | G | A | 0.4013 | 0.04476654 | 1.525E-18 | 0.3708543 | 995 | Suhre | Q15181; V9HWB5 | 80.358 |
| Plasma | PPID | E5KN55; Q08752 | rs8396 | 4 | 159630817 | C | T | 0.5011 | 0.0459752 | 3.395E-26 | 0.2864372 | 988 | Suhre | E5KN55; Q08752 | 118.796 |
| Plasma | PPIE | Q9UNP9; B3KSZ1 | rs12086750 | 1 | 40210468 | C | G | -0.3832 | 0.025 | 4.800E-53 | 0.370624 | 3301 | Sun | Q9UNP9; B3KSZ1 | 234.948 |
| Plasma | PPIL1 | A0A024RCX8; Q9Y3C6 | rs12194408 | 6 | 36839598 | G | C | -1.106 | 0.0705 | 1.900E-55 | 0.030241 | 3301 | Sun | A0A024RCX8; Q9Y3C6 | 246.112 |
| Plasma | PPP3CA; PPP3R1 | A0A0S2Z4C6; Q08209; A0A0S2Z4B5; P63098 | rs17266357 | 4 | 102721809 | C | T | 0.3681 | 0.04500467 | 8.670E-16 | 0.3099298 | 997 | Suhre | A0A0S2Z4C6; Q08209; A0A0S2Z4B5; P63098 | 66.899 |
| Plasma | PPT1 | P50897 | rs7533094 | 1 | 40559686 | A | G | -0.8621 | 0.0603 | 2.300E-46 | 0.041979 | 3301 | Sun | P50897 | 204.400 |
| Plasma | PRCP | B7Z7Q6; P42785; A0A024R5L0 | rs2229437 | 11 | 82564294 | G | T | 0.291 | 0.0315 | 2.800E-20 | 0.180887 | 3301 | Sun | B7Z7Q6; P42785; A0A024R5L0 | 85.342 |
| Plasma | PRSS22 | Q9GZN4 | rs3810801 | 16 | 2892370 | A | C | 0.3066 | 0.0256 | 3.700E-33 | 0.332823 | 3301 | Sun | Q9GZN4 | 143.438 |
| Plasma | PRTN3 | P24158 | rs10425544 | 19 | 836043 | C | T | 0.5991 | 0.0269 | 8.300E-110 | 0.712816 | 3301 | Sun | P24158 | 496.014 |
| Plasma | PSAPL1 | Q6NUJ1 | rs10023470 | 4 | 7434456 | G | A | 0.5481 | 0.0307 | 2.600E-71 | 0.184308 | 3301 | Sun | Q6NUJ1 | 318.745 |
| Plasma | PSG3 | Q16557 | rs2355433 | 19 | 43679088 | G | A | -0.6523 | 0.0239 | 1.600E-163 | 0.547653 | 3301 | Sun | Q16557 | 744.902 |
| Plasma | PSG4 | B3KQL2; Q96QL5; Q6P520; Q00888 | rs1138888 | 19 | 43696022 | A | T | -0.666 | 0.0245 | 5.400E-163 | 0.624394 | 3301 | Sun | B3KQL2; Q96QL5; Q6P520; Q00888 | 738.952 |
| Plasma | PTGDS | A0A024R8G3; P41222 | rs7019538 | 9 | 139861470 | C | T | 0.1979 | 0.0246 | 7.800E-16 | 0.524107 | 3301 | Sun | A0A024R8G3; P41222 | 64.717 |
| Plasma | PTGFRN | Q9P2B2 | rs4233450 | 1 | 117490261 | T | G | 1.0007 | 0.0283 | 1.000E-200 | 0.829141 | 3301 | Sun | Q9P2B2 | 1250.360 |
| Plasma | PTHLH | P12272; Q53XY9; A0A024RB29 | rs10843115 | 12 | 28307717 | T | C | 0.1862 | 0.0276 | 1.400E-11 | 0.271368 | 3301 | Sun | P12272; Q53XY9; A0A024RB29 | 45.514 |
| Plasma | PTN | A0A024R778; P21246 | rs1431093 | 7 | 137016297 | A | C | 0.2196 | 0.0248 | 7.900E-19 | 0.419185 | 3301 | Sun | A0A024R778; P21246 | 78.408 |
| Plasma | QDPR | A0A140VKA9; P09417 | rs28719835 | 4 | 17520066 | T | C | -0.5668 | 0.0275 | 1.000E-94 | 0.242578 | 3301 | Sun | A0A140VKA9; P09417 | 424.810 |
| Plasma | QPCTL | Q9NXS2 | rs17850756 | 19 | 46206262 | A | G | -0.2881 | 0.026 | 1.600E-28 | 0.327901 | 3301 | Sun | Q9NXS2 | 122.783 |
| Plasma | QSOX1 | A0A140VKE5; O00391; Q13876 | rs12371 | 1 | 180163390 | G | A | 0.6995 | 0.0426 | 1.300E-60 | 0.089018 | 3301 | Sun | A0A140VKE5; O00391; Q13876 | 269.623 |
| Plasma | QSOX2 | Q6ZRP7 | rs10858248 | 9 | 139108324 | G | A | -0.3568 | 0.0239 | 1.500E-50 | 0.502175 | 3301 | Sun | Q6ZRP7 | 222.871 |
| Plasma | RARRES1 | P49788 | rs61696028 | 3 | 158455703 | C | A | 1.3366 | 0.0266 | 1.000E-200 | 0.146047 | 3301 | Sun | P49788 | 2524.874 |
| Plasma | RARRES2 | A0A090N7U9; Q99969 | rs9640161 | 7 | 150045910 | A | C | 0.3473 | 0.04177132 | 3.049E-16 | 0.3708207 | 987 | Suhre | A0A090N7U9; Q99969 | 69.128 |
| Plasma | REG1A | A8K7G6; P05451 | rs11126696 | 2 | 79323888 | G | A | 0.265 | 0.021 | 2.180E-40 | 0.612 | 6861 | Yao | A8K7G6; P05451 | 159.240 |
| Plasma | REG4 | A0A024R0M1; Q9BYZ8 | rs79795228 | 1 | 120359286 | A | C | 0.7938 | 0.0995 | 1.400E-15 | 0.015404 | 3301 | Sun | A0A024R0M1; Q9BYZ8 | 63.647 |
| Plasma | RELT | A0A024R5N3; Q969Z4 | rs7952686 | 11 | 73128503 | T | C | 0.4125 | 0.0292 | 3.100E-45 | 0.204256 | 3301 | Sun | A0A024R5N3; Q969Z4 | 199.564 |
| Plasma | RET | P07949; Q9BTX6; A0A024R7T2 | rs2795507 | 10 | 43352894 | C | T | -0.2768 | 0.0305 | 1.100E-19 | 0.796047 | 3301 | Sun | P07949; Q9BTX6; A0A024R7T2 | 82.363 |
| Plasma | RETN | Q9HD89 | rs34124816 | 19 | 7733676 | C | A | -0.6083 | 0.0645 | 4.400E-21 | 0.037632 | 3301 | Sun | Q9HD89 | 88.944 |
| Plasma | RFESD | Q8TAC1; A0A024RAR3 | rs77881626 | 5 | 95017852 | G | T | -0.5972 | 0.0573 | 2.100E-25 | 0.04763 | 3301 | Sun | Q8TAC1; A0A024RAR3 | 108.625 |
| Plasma | RGMA | A0A0A0MTQ4; Q96B86 | rs3752102 | 15 | 93616014 | A | C | -0.2012 | 0.0245 | 2.500E-16 | 0.473455 | 3301 | Sun | A0A0A0MTQ4; Q96B86 | 67.441 |
| Plasma | RGMB | J3KNF6; Q6NW40 | rs1563317 | 5 | 97768486 | G | A | 0.2161 | 0.0245 | 1.200E-18 | 0.552752 | 3301 | Sun | J3KNF6; Q6NW40 | 77.800 |
| Plasma | RIDA | A0A024R9H2; P52758 | rs1462977 | 8 | 99115359 | G | A | -0.3387 | 0.0258 | 2.200E-39 | 0.322718 | 3301 | Sun | A0A024R9H2; P52758 | 172.342 |
| Plasma | RMDN1 | Q6N086; Q96DB5; E5RH53 | rs11781016 | 8 | 87529297 | C | A | 0.3167 | 0.0243 | 8.300E-39 | 0.489299 | 3301 | Sun | Q6N086; Q96DB5; E5RH53 | 169.857 |
| Plasma | RNASE1 | P07998; W0UV93 | rs17254387 | 14 | 21280678 | A | G | 0.2302 | 0.0273 | 3.000E-17 | 0.687505 | 3301 | Sun | P07998; W0UV93 | 71.103 |
| Plasma | RNASE2 | P10153; W0UV60 | rs56204594 | 14 | 21433367 | C | A | 0.2366 | 0.0244 | 3.200E-22 | 0.464292 | 3301 | Sun | P10153; W0UV60 | 94.026 |
| Plasma | RNASE4 | P34096; Q53XB4 | rs12588573 | 14 | 21146584 | T | C | -0.4494 | 0.0291 | 8.900E-54 | 0.224438 | 3301 | Sun | P34096; Q53XB4 | 238.495 |
| Plasma | RNASE6 | Q6IB39; Q93091 | rs11622942 | 14 | 21250846 | T | G | 0.9971 | 0.0236 | 1.000E-200 | 0.233384 | 3301 | Sun | Q6IB39; Q93091 | 1785.063 |
| Plasma | ROR1 | Q01973 | rs1408416 | 1 | 64614495 | T | G | -0.44 | 0.033 | 1.800E-40 | 0.161988 | 3301 | Sun | Q01973 | 177.778 |
| Plasma | RPN1 | P04843 | rs2712417 | 3 | 128345179 | G | A | -0.3447 | 0.0247 | 3.100E-44 | 0.618031 | 3301 | Sun | P04843 | 194.755 |
| Plasma | RRM2B | Q7LG56 | rs74589258 | 8 | 103215228 | G | A | 0.3254 | 0.0479 | 1.100E-11 | 0.07035 | 3301 | Sun | Q7LG56 | 46.149 |
| Plasma | RSPO3 | Q9BXY4 | rs2489623 | 6 | 127455821 | C | A | 0.27 | 0.0245 | 3.600E-28 | 0.534679 | 3301 | Sun | Q9BXY4 | 121.449 |
| Plasma | RTN4R | Q9BZR6 | rs75766 | 22 | 20174853 | A | C | -0.3498 | 0.0283 | 4.800E-35 | 0.748115 | 3301 | Sun | Q9BZR6 | 152.780 |
| Plasma | S100A4 | P26447 | rs58056804 | 1 | 153524706 | A | G | 0.5046 | 0.0565 | 4.500E-19 | 0.049682 | 3301 | Sun | P26447 | 79.762 |
| Plasma | SCARF1 | A8K6Z5; Q14162 | rs8071756 | 17 | 1574342 | G | A | 0.5897 | 0.04942652 | 9.358E-31 | 0.223671 | 997 | Suhre | A8K6Z5; Q14162 | 142.345 |
| Plasma | SCARF2 | A0A096LNX8; Q96GP6 | rs738086 | 22 | 20775556 | T | G | 0.2997 | 0.0318 | 4.800E-21 | 0.811314 | 3301 | Sun | A0A096LNX8; Q96GP6 | 88.822 |
| Plasma | SCG3 | Q8WXD2 | rs1378892 | 15 | 51964865 | C | T | 0.486 | 0.0277 | 1.000E-68 | 0.763554 | 3301 | Sun | Q8WXD2 | 307.831 |
| Plasma | SECTM1 | Q8WVN6 | rs4789763 | 17 | 80289284 | G | A | 0.2805 | 0.0246 | 3.800E-30 | 0.492828 | 3301 | Sun | Q8WVN6 | 130.016 |
| Plasma | SELL | A0A024R8Z0; P14151 | rs4987358 | 1 | 169665551 | T | G | -0.5165 | 0.0261 | 6.800E-87 | 0.27377 | 3301 | Sun | A0A024R8Z0; P14151 | 391.615 |
| Plasma | SELP | A0A024R8Y9; P16109; Q6NUL9; Q5R341 | rs6136 | 1 | 169563951 | G | T | -0.8073 | 0.0371 | 3.000E-105 | 0.107874 | 3301 | Sun | A0A024R8Y9; P16109; Q6NUL9; Q5R341 | 473.502 |
| Plasma | SEMA3C | B4E2I9; Q99985 | rs1019016 | 7 | 80570562 | T | G | 0.1751 | 0.0248 | 1.600E-12 | 0.581376 | 3301 | Sun | B4E2I9; Q99985 | 49.850 |
| Plasma | SEMA3E | O15041 | rs3757607 | 7 | 83034362 | C | G | -1.1204 | 0.0323 | 1.000E-200 | 0.12244 | 3301 | Sun | O15041 | 1203.209 |
| Plasma | SEMA3G | Q9NS98 | rs2016575 | 3 | 52477080 | C | T | -0.2687 | 0.0316 | 1.900E-17 | 0.815652 | 3301 | Sun | Q9NS98 | 72.304 |
| Plasma | SEMA5A | Q13591; X5DR95 | rs17329170 | 5 | 9547242 | A | G | 1.432 | 0.0324 | 1.000E-200 | 0.100634 | 3301 | Sun | Q13591; X5DR95 | 1953.422 |
| Plasma | SERPINA1 | E9KL23; P01009 | rs2749534 | 14 | 94809760 | G | A | 0.4267 | 0.05376264 | 5.551E-15 | 0.2011033 | 997 | Suhre | E9KL23; P01009 | 62.992 |
| Plasma | SERPINA3 | A0A024R6P0; P01011 | rs6575449 | 14 | 95097303 | T | C | -0.4323 | 0.05351082 | 1.903E-15 | 0.1859296 | 995 | Suhre | A0A024R6P0; P01011 | 65.266 |
| Plasma | SERPINA4 | P29622; A0A024R6I9 | rs10135681 | 14 | 95007744 | C | T | 0.2771 | 0.04221022 | 8.416E-11 | 0.3951856 | 997 | Suhre | P29622; A0A024R6I9 | 43.096 |
| Plasma | SERPINA4 | P29622; A0A024R6I9 | rs10139745 | 14 | 95035374 | A | G | 0.6166 | 0.0277 | 4.900E-110 | 0.221432 | 3301 | Sun | P29622; A0A024R6I9 | 495.504 |
| Plasma | SERPINE2 | A0A024R498; P07093; A0A024R451 | rs68066031 | 2 | 224880498 | C | T | -0.4261 | 0.0297 | 1.100E-46 | 0.258001 | 3301 | Sun | A0A024R498; P07093; A0A024R451 | 205.831 |
| Plasma | SERPINF1 | A0A140VKF3; P36955 | rs62088172 | 17 | 1666253 | T | C | -0.4449 | 0.0251 | 1.800E-70 | 0.344526 | 3301 | Sun | A0A140VKF3; P36955 | 314.179 |
| Plasma | SERPINF2 | P08697 | rs11657394 | 17 | 1636950 | A | C | -0.3459 | 0.0479 | 5.400E-13 | 0.077401 | 3301 | Sun | P08697 | 52.147 |
| Plasma | SIGLEC12 | Q96PQ1 | rs3826667 | 19 | 52004074 | T | C | -1.127 | 0.0275 | 1.000E-200 | 0.835439 | 3301 | Sun | Q96PQ1 | 1679.509 |
| Plasma | SIGLEC14 | Q08ET2 | rs1106476 | 19 | 52130637 | A | T | -1.1918 | 0.0317 | 1.000E-200 | 0.116912 | 3301 | Sun | Q08ET2 | 1413.475 |
| Plasma | SIGLEC6 | O43699; A0A024R4K4 | rs2124910 | 19 | 52025247 | T | C | 0.653 | 0.04336313 | 2.748E-46 | 0.4073515 | 993 | Suhre | O43699; A0A024R4K4 | 226.770 |
| Plasma | SIGLEC7 | Q9Y286 | rs140185670 | 19 | 51646140 | C | G | -0.6089 | 0.0482 | 1.700E-36 | 0.07999 | 3301 | Sun | Q9Y286 | 159.587 |
| Plasma | SIGLEC9 | Q9Y336 | rs2075803 | 19 | 51628529 | G | A | -1.2255 | 0.0123 | 1.000E-200 | 0.55088 | 3301 | Sun | Q9Y336 | 9926.963 |
| Plasma | SIRPA | P78324 | rs6136377 | 20 | 1896288 | G | A | -1.2106 | 0.0153 | 1.000E-200 | 0.368754 | 3301 | Sun | P78324 | 6260.636 |
| Plasma | SIRPB1 | O00241; Q5TFQ8; H9KV29 | rs3848788 | 20 | 1543066 | A | G | 0.7453 | 0.0239 | 1.000E-200 | 0.304983 | 3301 | Sun | O00241; Q5TFQ8; H9KV29 | 972.448 |
| Plasma | SIRPG | Q9P1W8 | rs6043409 | 20 | 1616206 | G | A | 0.2628 | 0.0261 | 8.100E-24 | 0.652477 | 3301 | Sun | Q9P1W8 | 101.384 |
| Plasma | SLAMF7 | Q9NQ25; B4DVL7; B4DW98 | rs11581248 | 1 | 160720074 | T | C | -1.2597 | 0.0295 | 1.000E-200 | 0.142448 | 3301 | Sun | Q9NQ25; B4DVL7; B4DW98 | 1823.435 |
| Plasma | SMOC1 | Q9H4F8; A0A024R6E0 | rs1958078 | 14 | 70354858 | C | A | 0.3765 | 0.0328 | 1.800E-30 | 0.833946 | 3301 | Sun | Q9H4F8; A0A024R6E0 | 131.760 |
| Plasma | SNCA | P37840; H6UYS5 | rs2245801 | 4 | 90757840 | C | T | -0.2507 | 0.0303 | 1.200E-16 | 0.788276 | 3301 | Sun | P37840; H6UYS5 | 68.458 |
| Plasma | SPARCL1 | A0A024RDE1; Q14515; B7ZB68; Q8N4S1 | rs7681694 | 4 | 88462729 | A | G | 0.5315 | 0.0244 | 2.700E-105 | 0.3366 | 3301 | Sun | A0A024RDE1; Q14515; B7ZB68; Q8N4S1 | 474.490 |
| Plasma | SPARCL1 | A0A024RDE1; Q14515; B7ZB68; Q8N4S1 | rs1462372 | 4 | 88478750 | C | T | 0.3214 | 0.04619973 | 6.309E-12 | 0.3274824 | 997 | Suhre | A0A024RDE1; Q14515; B7ZB68; Q8N4S1 | 48.396 |
| Plasma | SPATA20 | Q8TB22 | rs9890200 | 17 | 48624523 | C | A | -0.2532 | 0.0252 | 8.700E-24 | 0.375996 | 3301 | Sun | Q8TB22 | 100.955 |
| Plasma | SPINK2 | D6RI10; D6RC51; P20155; A0A087WTA9; A0A024RD95 | rs11941335 | 4 | 57689460 | T | C | 0.9996 | 0.083 | 2.200E-33 | 0.021062 | 3301 | Sun | D6RI10; D6RC51; P20155; A0A087WTA9; A0A024RD95 | 145.043 |
| Plasma | SPINK6 | Q6UWN8 | rs1432688 | 5 | 147603178 | G | A | 1.0579 | 0.0428 | 7.600E-135 | 0.924477 | 3301 | Sun | Q6UWN8 | 610.944 |
| Plasma | SPINT2 | A0A140VJV6; O43291 | rs71354995 | 19 | 38791841 | G | A | -0.9908 | 0.0227 | 1.000E-200 | 0.242563 | 3301 | Sun | A0A140VJV6; O43291 | 1905.111 |
| Plasma | SPINT3 | P49223 | rs6017591 | 20 | 44141041 | C | T | 0.5431 | 0.0227 | 6.000E-127 | 0.541306 | 3301 | Sun | P49223 | 572.411 |
| Plasma | SPOCK2 | Q92563; A0A024QZQ5 | rs1245540 | 10 | 73849752 | T | C | 0.2179 | 0.0243 | 3.300E-19 | 0.44884 | 3301 | Sun | Q92563; A0A024QZQ5 | 80.408 |
| Plasma | SPOCK3 | Q9BQ16; A0A0A0MTJ2; B4DI52 | rs17599599 | 4 | 167961738 | A | G | 0.8477 | 0.0765 | 1.500E-28 | 0.028272 | 3301 | Sun | Q9BQ16; A0A0A0MTJ2; B4DI52 | 122.790 |
| Plasma | SPON1 | Q9HCB6 | rs10832169 | 11 | 14066486 | A | G | 0.3748 | 0.0239 | 1.300E-55 | 0.490547 | 3301 | Sun | Q9HCB6 | 245.925 |
| Plasma | ST3GAL1 | A0A024R9L6; Q11201 | rs9643300 | 8 | 134503148 | T | C | -0.2571 | 0.0246 | 1.400E-25 | 0.562654 | 3301 | Sun | A0A024R9L6; Q11201 | 109.228 |
| Plasma | ST3GAL6 | A0A087WXB8; Q9Y274 | rs72934623 | 3 | 98509705 | A | G | -1.8784 | 0.0447 | 1.000E-200 | 0.053172 | 3301 | Sun | A0A087WXB8; Q9Y274 | 1765.880 |
| Plasma | SVEP1 | B3KQM1; Q4LDE5; Q5JB40 | rs61751937 | 9 | 113312231 | C | G | 1.1854 | 0.0702 | 5.200E-64 | 0.030029 | 3301 | Sun | B3KQM1; Q4LDE5; Q5JB40 | 285.138 |
| Plasma | SWAP70 | B3KUB9; E7EMB1; Q9UH65 | rs415895 | 11 | 9769562 | G | C | -0.2659 | 0.0256 | 3.500E-25 | 0.649843 | 3301 | Sun | B3KUB9; E7EMB1; Q9UH65 | 107.884 |
| Plasma | TAPBPL | Q9BX59 | rs2532497 | 12 | 6564210 | A | G | 1.2577 | 0.0163 | 1.000E-200 | 0.273692 | 3301 | Sun | Q9BX59 | 5953.590 |
| Plasma | TCN1 | P20061 | rs34528912 | 11 | 59631535 | T | C | -0.7109 | 0.0624 | 4.600E-30 | 0.04157 | 3301 | Sun | P20061 | 129.792 |
| Plasma | TCN2 | P20062 | rs4820885 | 22 | 31012756 | C | T | -0.7247 | 0.0212 | 1.000E-200 | 0.549103 | 3301 | Sun | P20062 | 1168.543 |
| Plasma | TEK | Q02763; Q59HG2 | rs35030851 | 9 | 27197486 | T | G | 0.5573 | 0.057 | 1.400E-22 | 0.046653 | 3301 | Sun | Q02763; Q59HG2 | 95.594 |
| Plasma | TEPSIN | Q96N21; A0A1B0GV70 | rs61745945 | 17 | 79205421 | A | G | -0.8768 | 0.0986 | 5.900E-19 | 0.016358 | 3301 | Sun | Q96N21; A0A1B0GV70 | 79.076 |
| Plasma | TFF1 | P04155 | rs3761376 | 21 | 43787038 | A | G | -0.2204 | 0.0291 | 3.900E-14 | 0.241766 | 3301 | Sun | P04155 | 57.364 |
| Plasma | TGFBI | A0A0S2Z4Q2; Q15582 | rs13159365 | 5 | 135389433 | T | C | -0.4461 | 0.0239 | 5.000E-78 | 0.507136 | 3301 | Sun | A0A0S2Z4Q2; Q15582 | 348.392 |
| Plasma | THBS2 | P35442 | rs73043857 | 6 | 169624900 | G | A | 0.8084 | 0.0383 | 6.900E-99 | 0.102347 | 3301 | Sun | P35442 | 445.508 |
| Plasma | THSD1 | A0A024R064; B3KTY7; Q9NS62 | rs41292808 | 13 | 52971517 | T | C | 0.9059 | 0.0757 | 5.500E-33 | 0.026681 | 3301 | Sun | A0A024R064; B3KTY7; Q9NS62 | 143.208 |
| Plasma | TIE1 | B4DTW8; P35590 | rs2275180 | 1 | 43773033 | G | A | 0.2538 | 0.0251 | 4.800E-24 | 0.623345 | 3301 | Sun | B4DTW8; P35590 | 102.244 |
| Plasma | TIMP3 | P35625 | rs2097326 | 22 | 33165020 | G | A | 0.9307 | 0.03967332 | 4.424E-97 | 0.2787298 | 992 | Suhre | P35625 | 550.329 |
| Plasma | TIMP4 | Q99727 | rs454615 | 3 | 12077010 | C | T | 0.4903 | 0.0318 | 1.700E-53 | 0.830831 | 3301 | Sun | Q99727 | 237.722 |
| Plasma | TIRAP | A0A024R3M4; P58753 | rs111577916 | 11 | 126071349 | T | G | -0.5707 | 0.0774 | 1.600E-13 | 0.024689 | 3301 | Sun | A0A024R3M4; P58753 | 54.367 |
| Plasma | TLR4; LY96 | O00206; Q9Y6Y9 | rs4986790 | 9 | 120475302 | G | A | -0.9019 | 0.0509 | 2.800E-70 | 0.05593 | 3301 | Sun | O00206; Q9Y6Y9 | 313.965 |
| Plasma | TMEM132A | Q24JP5 | rs11230521 | 11 | 60698732 | A | G | 0.6176 | 0.0274 | 2.000E-112 | 0.231444 | 3301 | Sun | Q24JP5 | 508.058 |
| Plasma | TMEM132C | Q8N3T6 | rs11059617 | 12 | 128757909 | T | A | -0.225 | 0.0257 | 2.200E-18 | 0.335066 | 3301 | Sun | Q8N3T6 | 76.648 |
| Plasma | TMEM190 | Q8WZ59 | rs4806666 | 19 | 55888095 | T | C | -0.997 | 0.0177 | 1.000E-200 | 0.438473 | 3301 | Sun | Q8WZ59 | 3172.808 |
| Plasma | TNFAIP6 | P98066 | rs289828 | 2 | 152137181 | T | C | -0.5479 | 0.0237 | 5.500E-118 | 0.62173 | 3301 | Sun | P98066 | 534.449 |
| Plasma | TNFRSF11A | Q9Y6Q6 | rs884205 | 18 | 60054857 | C | A | -0.2235 | 0.0292 | 2.000E-14 | 0.756399 | 3301 | Sun | Q9Y6Q6 | 58.585 |
| Plasma | TNFRSF6B | O95407 | rs62217798 | 20 | 62347189 | T | G | -0.224 | 0.0316 | 1.400E-12 | 0.784171 | 3301 | Sun | O95407 | 50.248 |
| Plasma | TNFSF12; TNFSF12-TNFSF13 | O43508; Q4ACW9; A0A0A6YY99 | rs12941509 | 17 | 7448288 | G | C | 0.3933 | 0.0266 | 2.200E-49 | 0.279301 | 3301 | Sun | O43508; Q4ACW9; A0A0A6YY99 | 218.617 |
| Plasma | TPSAB1; TPSB2 | P20231; Q15661; A0A140VJT7 | rs4984778 | 16 | 1297516 | C | G | 0.5167 | 0.0463218 | 2.678E-27 | 0.2846386 | 996 | Suhre | P20231; Q15661; A0A140VJT7 | 124.425 |
| Plasma | TPST1 | A0A024RDK9; O60507 | rs313829 | 7 | 65552497 | G | A | 0.2313 | 0.0262 | 1.100E-18 | 0.688554 | 3301 | Sun | A0A024RDK9; O60507 | 77.938 |
| Plasma | TPST2 | A0A024R1G9; O60704 | rs2283824 | 22 | 26924456 | A | G | 0.2429 | 0.0246 | 6.000E-23 | 0.416943 | 3301 | Sun | A0A024R1G9; O60704 | 97.496 |
| Plasma | TREML2 | Q5T2D2 | rs61998254 | 6 | 41166151 | G | A | 0.6927 | 0.0352 | 5.000E-86 | 0.126963 | 3301 | Sun | Q5T2D2 | 387.262 |
| Plasma | UCMA | A0A067XJP8; Q8WVF2; A0A067XKV3; A0A067XJX6 | rs2093847 | 10 | 13276534 | T | C | -0.5863 | 0.0382 | 3.100E-53 | 0.106703 | 3301 | Sun | A0A067XJP8; Q8WVF2; A0A067XKV3; A0A067XJX6 | 235.566 |
| Plasma | UNC5C | A8K385; O95185 | rs57091121 | 4 | 96444053 | T | A | 0.2694 | 0.0259 | 1.900E-25 | 0.336919 | 3301 | Sun | A8K385; O95185 | 108.192 |
| Plasma | UROS | A0A0S2Z4T8; P10746; A0A0S2Z5C5; Q5T3L8 | rs10794029 | 10 | 127561568 | G | A | -0.2329 | 0.0311 | 7.600E-14 | 0.794492 | 3301 | Sun | A0A0S2Z4T8; P10746; A0A0S2Z5C5; Q5T3L8 | 56.081 |
| Plasma | VEGFA | P15692; A0A024RD33; A0A024RD37; A0A0Y0IMM4; A2A2V4 | rs6921438 | 6 | 43925607 | A | G | -0.7023 | 0.0215 | 1.000E-200 | 0.496842 | 3301 | Sun | P15692; A0A024RD33; A0A024RD37; A0A0Y0IMM4; A2A2V4 | 1067.010 |
| Plasma | VEGFC | P49767 | rs41278571 | 4 | 177650866 | T | C | 1.7416 | 0.1215 | 1.300E-46 | 0.010126 | 3301 | Sun | P49767 | 205.468 |
| Plasma | VIT | Q6UXI7 | rs10490666 | 2 | 36932493 | T | A | 0.3471 | 0.0264 | 1.900E-39 | 0.304408 | 3301 | Sun | Q6UXI7 | 172.863 |
| Plasma | VSIR | Q9H7M9 | rs10762477 | 10 | 73531069 | G | A | 0.2705 | 0.0352 | 1.700E-14 | 0.14794 | 3301 | Sun | Q9H7M9 | 59.054 |
| Plasma | WFIKKN2 | C9J6G4; Q8TEU8 | rs7225465 | 17 | 48916159 | A | G | -0.6533 | 0.0238 | 9.300E-166 | 0.68203 | 3301 | Sun | C9J6G4; Q8TEU8 | 753.479 |
| Plasma | WISP1 | O95388 | rs35472615 | 8 | 134197537 | A | G | 0.5113 | 0.0263 | 1.700E-84 | 0.274129 | 3301 | Sun | O95388 | 377.955 |
| Plasma | XCL1 | P47992 | rs4656599 | 1 | 168503386 | T | C | 0.4295 | 0.0311 | 2.000E-43 | 0.189892 | 3301 | Sun | P47992 | 190.724 |
| Plasma | ACE | P12821 | rs4344 | 17 | 61566724 | A | G | -0.583 | 0.022 | 8.500E-136 | 0.499 | 3200 | Emilsson | P12821 | 702.250 |
| Plasma | ACHE | P22303 | rs4727469 | 7 | 100509163 | C | T | -0.297 | 0.023 | 7.500E-37 | 0.524 | 3200 | Emilsson | P22303 | 166.747 |
| Plasma | ACP6 | Q9NPH0 | rs2153463 | 1 | 147124310 | G | T | 0.944 | 0.022 | 1.000E-200 | 0.739 | 3200 | Emilsson | Q9NPH0 | 1841.190 |
| Plasma | ADAM12 | O43184 | rs10794057 | 10 | 127729734 | T | C | -0.209 | 0.025 | 1.200E-16 | 0.398 | 3200 | Emilsson | O43184 | 69.890 |
| Plasma | ADAM19 | Q9H013 | rs7728609 | 5 | 156935524 | C | T | 0.246 | 0.026 | 1.200E-21 | 0.675 | 3200 | Emilsson | Q9H013 | 89.521 |
| Plasma | ADAM22 | Q9P0K1 | rs6966166 | 7 | 87455155 | T | C | -0.177 | 0.026 | 9.800E-12 | 0.478 | 3200 | Emilsson | Q9P0K1 | 46.345 |
| Plasma | ADH4 | P08319 | rs1800759 | 4 | 100065509 | G | T | 0.157 | 0.025 | 4.100E-10 | 0.622 | 3200 | Emilsson | P08319 | 39.438 |
| Plasma | ADH5 | P11766 | rs1453873 | 4 | 100022571 | C | T | -0.284 | 0.027 | 2.500E-25 | 0.742 | 3200 | Emilsson | P11766 | 110.639 |
| Plasma | ADH7 | P40394 | rs17529509 | 4 | 100351436 | C | A | 0.401 | 0.048 | 8.000E-17 | 0.927 | 3200 | Emilsson | P40394 | 69.792 |
| Plasma | ADIPOQ | Q15848 | rs143257534 | 3 | 186551888 | C | T | 0.425 | 0.072 | 3.300E-09 | 0.974 | 3200 | Emilsson | Q15848 | 34.843 |
| Plasma | AFP | P02771 | rs6829551 | 4 | 74173715 | T | C | 0.187 | 0.031 | 2.400E-09 | 0.812 | 3200 | Emilsson | P02771 | 36.388 |
| Plasma | AKR7A2 | O43488 | rs144376466 | 1 | 19600538 | C | T | 0.436 | 0.042 | 1.900E-24 | 0.912 | 3200 | Emilsson | O43488 | 107.764 |
| Plasma | ALPG | P10696 | rs10933394 | 2 | 233249080 | T | C | -0.192 | 0.026 | 1.000E-13 | 0.642 | 3200 | Emilsson | P10696 | 54.533 |
| Plasma | ALPP | NA | rs12478529 | 2 | 233286654 | C | T | -0.354 | 0.028 | 2.500E-35 | 0.778 | 3200 | Emilsson | NA | 159.842 |
| Plasma | AMY2B | P19961 | rs17014913 | 1 | 104105635 | A | G | 0.433 | 0.034 | 3.200E-36 | 0.839 | 3200 | Emilsson | P19961 | 162.188 |
| Plasma | AOC1 | P19801 | rs10452848 | 7 | 150523544 | A | G | -0.765 | 0.021 | 1.000E-200 | 0.498 | 3200 | Emilsson | P19801 | 1327.041 |
| Plasma | AP1G2 | O75843 | rs12897422 | 14 | 24033027 | G | A | 0.268 | 0.035 | 1.300E-14 | 0.858 | 3200 | Emilsson | O75843 | 58.632 |
| Plasma | APOA1 | P02647 | rs75507001 | 11 | 116577694 | C | A | 0.55 | 0.061 | 1.700E-19 | 0.957 | 3200 | Emilsson | P02647 | 81.295 |
| Plasma | APOBEC3G | Q9HC16 | rs738469 | 22 | 39510995 | A | G | 0.245 | 0.042 | 4.500E-09 | 0.903 | 3200 | Emilsson | Q9HC16 | 34.028 |
| Plasma | APOF | Q13790 | rs808919 | 12 | 56647911 | C | G | 0.321 | 0.04 | 6.900E-16 | 0.903 | 3200 | Emilsson | Q13790 | 64.401 |
| Plasma | APOH | P02749 | rs1801690 | 17 | 64208285 | C | G | 0.552 | 0.053 | 6.500E-25 | 0.943 | 3200 | Emilsson | P02749 | 108.474 |
| Plasma | ARHGAP1 | Q07960 | rs5899 | 11 | 46747662 | C | T | 0.835 | 0.111 | 8.400E-14 | 0.989 | 3200 | Emilsson | Q07960 | 56.588 |
| Plasma | ASIP | P42127 | rs62212171 | 20 | 32987687 | T | C | -0.814 | 0.037 | 9.600E-98 | 0.89 | 3200 | Emilsson | P42127 | 484.000 |
| Plasma | AZU1 | P20160 | rs351976 | 19 | 806673 | T | C | 0.216 | 0.027 | 1.500E-15 | 0.709 | 3200 | Emilsson | P20160 | 64.000 |
| Plasma | B3GALT6 | Q96L58 | rs3766186 | 1 | 1162435 | C | A | 0.271 | 0.041 | 6.500E-11 | 0.899 | 3200 | Emilsson | Q96L58 | 43.689 |
| Plasma | B3GLCT | Q6Y288 | rs9544399 | 13 | 31885907 | A | G | 0.278 | 0.031 | 1.600E-19 | 0.791 | 3200 | Emilsson | Q6Y288 | 80.420 |
| Plasma | B3GNT8 | Q7Z7M8 | rs284663 | 19 | 41932612 | T | C | 0.744 | 0.021 | 1.000E-200 | 0.56 | 3200 | Emilsson | Q7Z7M8 | 1255.184 |
| Plasma | B4GALT6 | Q9UBX8 | rs113222817 | 18 | 29216314 | T | G | 1.143 | 0.063 | 8.100E-71 | 0.965 | 3200 | Emilsson | Q9UBX8 | 329.163 |
| Plasma | BCAN | Q96GW7 | rs7541549 | 1 | 156588439 | T | C | 0.201 | 0.029 | 5.600E-12 | 0.767 | 3200 | Emilsson | Q96GW7 | 48.039 |
| Plasma | BOC | Q9BWV1 | rs73235147 | 3 | 112973955 | A | T | -0.241 | 0.032 | 1.100E-13 | 0.833 | 3200 | Emilsson | Q9BWV1 | 56.720 |
| Plasma | BPIFA2 | Q96DR5 | rs141715080 | 20 | 31742114 | C | T | 0.429 | 0.076 | 2.000E-08 | 0.973 | 3200 | Emilsson | Q96DR5 | 31.863 |
| Plasma | BTD | P43251 | rs13100619 | 3 | 15549967 | A | C | 1.431 | 0.058 | 4.400E-122 | 0.962 | 3200 | Emilsson | P43251 | 608.728 |
| Plasma | BTNL8 | Q6UX41 | rs7721375 | 5 | 180439650 | C | T | 0.273 | 0.024 | 1.400E-28 | 0.507 | 3200 | Emilsson | Q6UX41 | 129.391 |
| Plasma | C1QL1 | O75973 | rs9915692 | 17 | 43034207 | A | G | -0.279 | 0.023 | 3.400E-32 | 0.463 | 3200 | Emilsson | O75973 | 147.147 |
| Plasma | C1QTNF3 | Q9BXJ4 | rs840390 | 5 | 34018623 | G | A | 0.203 | 0.037 | 2.800E-08 | 0.868 | 3200 | Emilsson | Q9BXJ4 | 30.102 |
| Plasma | C1RL | Q9NZP8 | rs6488561 | 12 | 7246894 | G | A | 0.27 | 0.027 | 5.600E-24 | 0.695 | 3200 | Emilsson | Q9NZP8 | 100.000 |
| Plasma | C3 | P01024 | rs163494 | 19 | 6724340 | C | T | 0.264 | 0.031 | 3.400E-17 | 0.805 | 3200 | Emilsson | P01024 | 72.524 |
| Plasma | C4BPA | P04003 | rs11120218 | 1 | 207278451 | G | A | -0.429 | 0.038 | 1.500E-28 | 0.889 | 3200 | Emilsson | P04003 | 127.452 |
| Plasma | C5 | P01031 | rs1035029 | 9 | 123742818 | A | G | 0.182 | 0.025 | 2.100E-13 | 0.611 | 3200 | Emilsson | P01031 | 52.998 |
| Plasma | C9 | P02748 | rs265721 | 5 | 39354069 | G | A | -0.375 | 0.06 | 5.900E-10 | 0.959 | 3200 | Emilsson | P02748 | 39.063 |
| Plasma | CA1 | P00915 | rs2453868 | 8 | 86302696 | T | C | -0.147 | 0.025 | 2.200E-09 | 0.582 | 3200 | Emilsson | P00915 | 34.574 |
| Plasma | CA8 | P35219 | rs7009482 | 8 | 61195053 | G | A | -0.188 | 0.025 | 5.900E-14 | 0.589 | 3200 | Emilsson | P35219 | 56.550 |
| Plasma | CALCOCO2 | Q13137 | rs550510 | 17 | 46926615 | G | A | 0.19 | 0.033 | 1.000E-08 | 0.843 | 3200 | Emilsson | Q13137 | 33.150 |
| Plasma | CAPG | P40121 | rs143448563 | 2 | 85610023 | A | G | 1.4 | 0.094 | 1.000E-48 | 0.983 | 3200 | Emilsson | P40121 | 221.820 |
| Plasma | CCL27 | Q9Y4X3 | rs2070074 | 9 | 34649442 | A | G | 0.321 | 0.041 | 1.000E-14 | 0.907 | 3200 | Emilsson | Q9Y4X3 | 61.297 |
| Plasma | CCL4L1 | Q8NHW4 | rs2687507 | 17 | 34433964 | C | T | -0.239 | 0.029 | 1.500E-16 | 0.747 | 3200 | Emilsson | Q8NHW4 | 67.920 |
| Plasma | CD274 | Q9NZQ7 | rs1411262 | 9 | 5459419 | C | T | 0.31 | 0.027 | 1.700E-29 | 0.736 | 3200 | Emilsson | Q9NZQ7 | 131.824 |
| Plasma | CD300A | Q9UGN4 | rs2272111 | 17 | 72469966 | G | A | 0.435 | 0.028 | 1.200E-52 | 0.788 | 3200 | Emilsson | Q9UGN4 | 241.358 |
| Plasma | CD300E | Q496F6 | rs8081669 | 17 | 72614611 | G | A | 0.27 | 0.025 | 4.200E-27 | 0.551 | 3200 | Emilsson | Q496F6 | 116.640 |
| Plasma | CD7 | P09564 | rs116473040 | 17 | 80291652 | A | G | 0.962 | 0.057 | 1.300E-61 | 0.955 | 3200 | Emilsson | P09564 | 284.840 |
| Plasma | CD8A | P01732 | rs111976570 | 2 | 87014112 | A | C | -0.567 | 0.031 | 7.300E-73 | 0.81 | 3200 | Emilsson | P01732 | 334.536 |
| Plasma | CDCP1 | Q9H5V8 | rs7621542 | 3 | 45206484 | C | T | 0.18 | 0.031 | 5.900E-09 | 0.8 | 3200 | Emilsson | Q9H5V8 | 33.715 |
| Plasma | CDH11 | P55287 | rs59614634 | 16 | 65029943 | A | T | 0.803 | 0.036 | 3.400E-101 | 0.89 | 3200 | Emilsson | P55287 | 497.538 |
| Plasma | CFHR1 | Q03591 | rs57809726 | 1 | 196841377 | A | G | 1.295 | 0.027 | 1.000E-200 | 0.862 | 3200 | Emilsson | Q03591 | 2300.446 |
| Plasma | CFHR4 | Q92496 | rs4915559 | 1 | 196886770 | T | C | 0.655 | 0.026 | 1.700E-131 | 0.753 | 3200 | Emilsson | Q92496 | 634.652 |
| Plasma | CHST12 | Q9NRB3 | rs2969076 | 7 | 2473747 | A | G | -0.235 | 0.036 | 7.300E-11 | 0.872 | 3200 | Emilsson | Q9NRB3 | 42.612 |
| Plasma | CLEC11A | Q9Y240 | rs13866 | 19 | 51228746 | C | T | 0.154 | 0.026 | 4.000E-09 | 0.691 | 3200 | Emilsson | Q9Y240 | 35.083 |
| Plasma | CLEC1B | Q9P126 | rs544605 | 12 | 10146707 | T | C | -0.504 | 0.028 | 4.800E-70 | 0.781 | 3200 | Emilsson | Q9P126 | 324.000 |
| Plasma | CLEC4C | Q8WTT0 | rs11055602 | 12 | 7904111 | T | G | -0.95 | 0.019 | 1.000E-200 | 0.567 | 3200 | Emilsson | Q8WTT0 | 2500.000 |
| Plasma | CLN5 | O75503 | rs7996555 | 13 | 77562492 | C | T | 0.35 | 0.055 | 1.500E-10 | 0.946 | 3200 | Emilsson | O75503 | 40.496 |
| Plasma | CNP | P09543 | rs12602950 | 17 | 40123829 | A | G | 0.187 | 0.027 | 3.800E-12 | 0.704 | 3200 | Emilsson | P09543 | 47.968 |
| Plasma | CPQ | Q9Y646 | rs145746079 | 8 | 97832518 | T | C | 1.192 | 0.073 | 7.100E-58 | 0.972 | 3200 | Emilsson | Q9Y646 | 266.629 |
| Plasma | CRHBP | P24387 | rs6414971 | 5 | 76170674 | T | A | -0.511 | 0.026 | 2.900E-79 | 0.741 | 3200 | Emilsson | P24387 | 386.274 |
| Plasma | CRLF1 | O75462 | rs2238647 | 19 | 18710535 | G | A | -0.286 | 0.029 | 1.400E-22 | 0.778 | 3200 | Emilsson | O75462 | 97.260 |
| Plasma | CROT | Q9UKG9 | rs77463367 | 7 | 87006034 | C | G | 0.348 | 0.047 | 1.600E-13 | 0.93 | 3200 | Emilsson | Q9UKG9 | 54.823 |
| Plasma | CRTAC1 | Q9NQ79 | rs588061 | 10 | 99642731 | C | T | 0.346 | 0.024 | 8.600E-47 | 0.529 | 3200 | Emilsson | Q9NQ79 | 207.840 |
| Plasma | CRTAM | O95727 | rs2370794 | 11 | 122714782 | A | G | -0.236 | 0.026 | 5.800E-20 | 0.668 | 3200 | Emilsson | O95727 | 82.391 |
| Plasma | CRYZ | Q08257 | rs3819946 | 1 | 75175886 | T | C | -1.175 | 0.029 | 1.000E-200 | 0.861 | 3200 | Emilsson | Q08257 | 1641.647 |
| Plasma | CST4 | P01036 | rs7270028 | 20 | 23681073 | C | A | 0.465 | 0.039 | 1.800E-31 | 0.896 | 3200 | Emilsson | P01036 | 142.160 |
| Plasma | CTGF | P29279 | rs9388953 | 6 | 132297509 | G | A | 0.353 | 0.03 | 8.700E-32 | 0.789 | 3200 | Emilsson | P29279 | 138.454 |
| Plasma | CTSC | P53634 | rs55897509 | 11 | 88066714 | A | C | -0.926 | 0.041 | 2.300E-107 | 0.918 | 3200 | Emilsson | P53634 | 510.099 |
| Plasma | CXCL10 | P02778 | rs4859589 | 4 | 76948299 | G | A | 0.133 | 0.024397803 | 5.000E-08 | 0.505 | 3200 | Emilsson | P02778 | 29.717 |
| Plasma | CYB5D2 | Q8WUJ1 | rs77246175 | 17 | 4072761 | G | C | -0.342 | 0.046 | 2.000E-13 | 0.921 | 3200 | Emilsson | Q8WUJ1 | 55.276 |
| Plasma | CYTL1 | Q9NRR1 | rs62291616 | 4 | 5052063 | T | C | -0.354 | 0.029 | 1.200E-32 | 0.814 | 3200 | Emilsson | Q9NRR1 | 149.008 |
| Plasma | DEFA1 | P59665 | rs4284061 | 8 | 6878257 | A | T | 0.171 | 0.024 | 3.300E-12 | 0.431 | 3200 | Emilsson | P59665 | 50.766 |
| Plasma | DKK2 | Q9UBU2 | rs77571736 | 4 | 107864984 | G | C | 0.945 | 0.047 | 2.200E-83 | 0.954 | 3200 | Emilsson | Q9UBU2 | 404.267 |
| Plasma | DNER | Q8NFT8 | rs35032874 | 2 | 230309360 | T | G | 0.162 | 0.029 | 1.700E-08 | 0.741 | 3200 | Emilsson | Q8NFT8 | 31.206 |
| Plasma | DPEP1 | P16444 | rs423135 | 16 | 89740873 | G | A | -0.313 | 0.024 | 5.500E-39 | 0.429 | 3200 | Emilsson | P16444 | 170.085 |
| Plasma | DUT | P33316 | rs117540572 | 15 | 48670241 | G | A | -0.339 | 0.05 | 2.100E-11 | 0.939 | 3200 | Emilsson | P33316 | 45.968 |
| Plasma | DYNLL2 | Q96FJ2 | rs35729384 | 17 | 56178900 | C | T | 0.161 | 0.025 | 2.900E-10 | 0.698 | 3200 | Emilsson | Q96FJ2 | 41.474 |
| Plasma | EBI3 | Q14213 | rs10409421 | 19 | 4254752 | A | G | 1.049 | 0.019 | 1.000E-200 | 0.716 | 3200 | Emilsson | Q14213 | 3048.202 |
| Plasma | ECH1 | Q13011 | rs4802890 | 19 | 39304402 | G | A | -0.533 | 0.044 | 2.200E-33 | 0.92 | 3200 | Emilsson | Q13011 | 146.740 |
| Plasma | ENDOU | P21128 | rs2072117 | 12 | 48131728 | G | A | -0.163 | 0.026 | 3.800E-10 | 0.711 | 3200 | Emilsson | P21128 | 39.303 |
| Plasma | EPHA2 | P29317 | rs28629977 | 1 | 16497272 | C | G | -0.148 | 0.023 | 2.000E-10 | 0.564 | 3200 | Emilsson | P29317 | 41.406 |
| Plasma | EPHB6 | O15197 | rs7789303 | 7 | 142552547 | A | G | 0.239 | 0.026 | 6.300E-20 | 0.725 | 3200 | Emilsson | O15197 | 84.499 |
| Plasma | F13B | P05160 | rs12116643 | 1 | 196973183 | T | C | 0.547 | 0.033 | 1.200E-60 | 0.863 | 3200 | Emilsson | P05160 | 274.756 |
| Plasma | FBLN1 | P23142 | rs67136035 | 22 | 45813433 | T | D | 0.132 | 0.024214361 | 5.000E-08 | 0.541 | 3200 | Emilsson | P23142 | 29.717 |
| Plasma | FCN3 | O75636 | rs2474283 | 1 | 27716213 | T | C | 0.716 | 0.048 | 1.100E-48 | 0.933 | 3200 | Emilsson | O75636 | 222.507 |
| Plasma | FGFBP3 | Q8TAT2 | rs11186737 | 10 | 93666349 | C | T | 0.302 | 0.027 | 5.900E-28 | 0.746 | 3200 | Emilsson | Q8TAT2 | 125.108 |
| Plasma | FGFR3 | P22607 | rs2403274 | 4 | 1754515 | C | G | 0.177 | 0.029 | 6.700E-10 | 0.752 | 3200 | Emilsson | P22607 | 37.252 |
| Plasma | FGL1 | Q08830 | rs7815429 | 8 | 17737357 | T | C | -0.446 | 0.025 | 3.300E-70 | 0.313 | 3200 | Emilsson | Q08830 | 318.266 |
| Plasma | FKBP7 | Q9Y680 | rs10930831 | 2 | 179303976 | G | C | -0.365 | 0.027 | 2.000E-39 | 0.768 | 3200 | Emilsson | Q9Y680 | 182.750 |
| Plasma | FMOD | Q06828 | rs4971253 | 1 | 203321414 | G | A | 0.25 | 0.045 | 2.700E-08 | 0.921 | 3200 | Emilsson | Q06828 | 30.864 |
| Plasma | FTCD | O95954 | rs149024257 | 21 | 47686213 | G | A | -0.287 | 0.051 | 1.900E-08 | 0.938 | 3200 | Emilsson | O95954 | 31.668 |
| Plasma | GAA | P10253 | rs12450199 | 17 | 78076592 | A | C | 0.369 | 0.025 | 1.400E-48 | 0.647 | 3200 | Emilsson | P10253 | 217.858 |
| Plasma | GALNT16 | Q8N428 | rs12100668 | 14 | 69793475 | G | A | -0.194 | 0.025 | 4.400E-15 | 0.408 | 3200 | Emilsson | Q8N428 | 60.218 |
| Plasma | GDI2 | P50395 | rs2890364 | 10 | 5833748 | G | A | 0.219 | 0.029 | 9.100E-14 | 0.774 | 3200 | Emilsson | P50395 | 57.029 |
| Plasma | GKN2 | Q86XP6 | rs13008230 | 2 | 69154583 | T | G | 0.942 | 0.076 | 2.700E-34 | 0.973 | 3200 | Emilsson | Q86XP6 | 153.630 |
| Plasma | GLO1 | Q04760 | rs12209477 | 6 | 38669799 | C | G | 0.168 | 0.026 | 7.100E-11 | 0.66 | 3200 | Emilsson | Q04760 | 41.751 |
| Plasma | GNMT | Q14749 | rs9471987 | 6 | 42944140 | A | G | 0.4 | 0.024 | 2.400E-61 | 0.402 | 3200 | Emilsson | Q14749 | 277.778 |
| Plasma | GNPTG | Q9UJJ9 | rs4984820 | 16 | 1407809 | C | T | -0.344 | 0.046 | 8.000E-14 | 0.923 | 3200 | Emilsson | Q9UJJ9 | 55.924 |
| Plasma | GSN | P06396 | rs76331566 | 9 | 124009014 | C | T | 0.398 | 0.068 | 5.000E-09 | 0.969 | 3200 | Emilsson | P06396 | 34.257 |
| Plasma | GSTM3 | P21266 | rs115929572 | 1 | 110246053 | G | A | -0.249 | 0.031617952 | 3.400E-15 | 0.935 | 3200 | Emilsson | P21266 | 62.020 |
| Plasma | HDGF | P51858 | rs4399146 | 1 | 156713558 | G | A | 0.269 | 0.027 | 3.200E-23 | 0.717 | 3200 | Emilsson | P51858 | 99.261 |
| Plasma | HIBCH | Q6NVY1 | rs291447 | 2 | 191177005 | A | C | -0.631 | 0.023 | 2.900E-152 | 0.393 | 3200 | Emilsson | Q6NVY1 | 752.667 |
| Plasma | HPSE | Q9Y251 | rs11732810 | 4 | 84230878 | G | T | 0.541 | 0.028 | 7.700E-78 | 0.773 | 3200 | Emilsson | Q9Y251 | 373.318 |
| Plasma | HSD17B14 | Q9BPX1 | rs473464 | 19 | 49334248 | T | C | 0.145 | 0.026 | 2.700E-08 | 0.498 | 3200 | Emilsson | Q9BPX1 | 31.102 |
| Plasma | IFNLR1 | Q8IU57 | rs12046369 | 1 | 24526135 | G | T | 0.244 | 0.035 | 3.300E-12 | 0.853 | 3200 | Emilsson | Q8IU57 | 48.601 |
| Plasma | IGDCC4 | Q8TDY8 | rs8034057 | 15 | 65789430 | G | A | 0.344 | 0.042 | 3.500E-16 | 0.905 | 3200 | Emilsson | Q8TDY8 | 67.084 |
| Plasma | IGFBP5 | P24593 | rs139739387 | 2 | 217402489 | A | G | 0.608 | 0.098 | 7.000E-10 | 0.984 | 3200 | Emilsson | P24593 | 38.491 |
| Plasma | IL10RB | Q08334 | rs2834167 | 21 | 34640788 | A | G | 0.16 | 0.028 | 1.100E-08 | 0.732 | 3200 | Emilsson | Q08334 | 32.653 |
| Plasma | IL1R1 | P14778 | rs7588201 | 2 | 102746276 | A | C | 0.163 | 0.026 | 2.600E-10 | 0.722 | 3200 | Emilsson | P14778 | 39.303 |
| Plasma | IL7 | P13232 | rs72666886 | 8 | 79728782 | C | T | 0.34 | 0.042 | 5.300E-16 | 0.903 | 3200 | Emilsson | P13232 | 65.533 |
| Plasma | IMPAD1 | Q9NX62 | rs112433249 | 8 | 57876576 | T | C | 1.685 | 0.136 | 1.700E-34 | 0.992 | 3200 | Emilsson | Q9NX62 | 153.505 |
| Plasma | INPP5B | P32019 | rs61778082 | 1 | 38306356 | T | C | 0.547 | 0.034 | 2.100E-55 | 0.865 | 3200 | Emilsson | P32019 | 258.831 |
| Plasma | ITIH2 | P19823 | rs73621225 | 10 | 7740905 | T | A | -1.181 | 0.044 | 1.400E-142 | 0.933 | 3200 | Emilsson | P19823 | 720.434 |
| Plasma | JAM3 | Q9BX67 | rs655627 | 11 | 134021859 | A | G | 0.184 | 0.024 | 4.100E-14 | 0.566 | 3200 | Emilsson | Q9BX67 | 58.778 |
| Plasma | KIAA1549L | Q6ZVL6 | rs12792396 | 11 | 33417110 | G | A | 0.439 | 0.025 | 7.100E-68 | 0.381 | 3200 | Emilsson | Q6ZVL6 | 308.354 |
| Plasma | KIAA2013 | Q8IYS2 | rs11588551 | 1 | 11941936 | T | C | 0.178 | 0.025 | 2.000E-12 | 0.418 | 3200 | Emilsson | Q8IYS2 | 50.694 |
| Plasma | KLK10 | O43240 | rs2569454 | 19 | 51523203 | C | T | 0.405 | 0.026 | 4.500E-54 | 0.641 | 3200 | Emilsson | O43240 | 242.641 |
| Plasma | KLK13 | Q9UKR3 | rs3760739 | 19 | 51538561 | G | T | 0.388 | 0.025 | 3.900E-54 | 0.659 | 3200 | Emilsson | Q9UKR3 | 240.870 |
| Plasma | KLRB1 | Q12918 | rs3933456 | 12 | 9753788 | C | A | 0.213 | 0.026 | 2.100E-16 | 0.625 | 3200 | Emilsson | Q12918 | 67.114 |
| Plasma | LAG3 | P18627 | rs3782735 | 12 | 6885076 | A | G | 0.154 | 0.025 | 4.300E-10 | 0.607 | 3200 | Emilsson | P18627 | 37.946 |
| Plasma | LANCL1 | O43813 | rs187097936 | 2 | 211362949 | C | G | 0.781 | 0.103 | 3.700E-14 | 0.985 | 3200 | Emilsson | O43813 | 57.495 |
| Plasma | LBP | P18428 | rs73112473 | 20 | 37006729 | C | T | 1.009 | 0.043 | 2.400E-112 | 0.922 | 3200 | Emilsson | P18428 | 550.612 |
| Plasma | LEAP2 | Q969E1 | rs57880964 | 5 | 132210674 | G | C | -0.3 | 0.033 | 4.100E-19 | 0.838 | 3200 | Emilsson | Q969E1 | 82.645 |
| Plasma | LECT2 | O14960 | rs248160 | 5 | 135293512 | T | C | -0.312 | 0.026 | 3.700E-33 | 0.67 | 3200 | Emilsson | O14960 | 144.000 |
| Plasma | LGALS3BP | Q08380 | rs4789847 | 17 | 77004644 | A | G | -0.196 | 0.033 | 3.900E-09 | 0.829 | 3200 | Emilsson | Q08380 | 35.276 |
| Plasma | LGALS4 | P56470 | rs55945853 | 19 | 39230046 | G | A | 0.181 | 0.024 | 7.700E-14 | 0.51 | 3200 | Emilsson | P56470 | 56.877 |
| Plasma | LGALS9 | O00182 | rs62055780 | 17 | 25971795 | T | C | 0.203 | 0.03 | 7.900E-12 | 0.753 | 3200 | Emilsson | O00182 | 45.788 |
| Plasma | LGMN | Q99538 | rs7140705 | 14 | 93224207 | T | G | -0.268 | 0.045 | 3.600E-09 | 0.913 | 3200 | Emilsson | Q99538 | 35.469 |
| Plasma | LILRA3 | Q8N6C8 | rs398217 | 19 | 54793038 | A | G | 1.243 | 0.02 | 1.000E-200 | 0.773 | 3200 | Emilsson | Q8N6C8 | 3862.623 |
| Plasma | LRP11 | Q86VZ4 | rs9371533 | 6 | 150210681 | A | G | 0.825 | 0.021 | 1.000E-200 | 0.376 | 3200 | Emilsson | Q86VZ4 | 1543.367 |
| Plasma | LRP12 | Q9Y561 | rs72679151 | 8 | 105627117 | C | T | -0.211 | 0.03 | 2.600E-12 | 0.796 | 3200 | Emilsson | Q9Y561 | 49.468 |
| Plasma | LRP8 | Q14114 | rs10218811 | 1 | 53805882 | A | G | 0.166 | 0.027 | 9.800E-10 | 0.725 | 3200 | Emilsson | Q14114 | 37.800 |
| Plasma | LRRC4C | Q9HCJ2 | rs998447 | 11 | 40310789 | A | C | -0.137 | 0.024 | 2.200E-08 | 0.538 | 3200 | Emilsson | Q9HCJ2 | 32.585 |
| Plasma | LSAMP | Q13449 | rs17646258 | 3 | 116038249 | C | T | 0.222 | 0.031 | 1.500E-12 | 0.829 | 3200 | Emilsson | Q13449 | 51.284 |
| Plasma | LYVE1 | Q9Y5Y7 | rs114527818 | 11 | 10619041 | T | A | -0.369 | 0.055 | 3.300E-11 | 0.951 | 3200 | Emilsson | Q9Y5Y7 | 45.012 |
| Plasma | MAN1A2 | O60476 | rs1289863 | 1 | 117854689 | C | T | 0.21 | 0.026 | 1.500E-15 | 0.692 | 3200 | Emilsson | O60476 | 65.237 |
| Plasma | MAN1C1 | Q9NR34 | rs3767879 | 1 | 26070909 | C | T | 0.172 | 0.023 | 9.300E-14 | 0.465 | 3200 | Emilsson | Q9NR34 | 55.924 |
| Plasma | MANF | P55145 | rs1552074 | 3 | 51408051 | T | C | 0.391 | 0.035 | 2.600E-28 | 0.859 | 3200 | Emilsson | P55145 | 124.801 |
| Plasma | MENT | Q9BUN1 | rs12759273 | 1 | 151045024 | C | A | 0.669 | 0.116 | 8.600E-09 | 0.988 | 3200 | Emilsson | Q9BUN1 | 33.261 |
| Plasma | METTL24 | Q5JXM2 | rs12189608 | 6 | 110573292 | A | T | 0.378 | 0.061 | 7.400E-10 | 0.956 | 3200 | Emilsson | Q5JXM2 | 38.399 |
| Plasma | NCAM2 | O15394 | rs2826851 | 21 | 22835946 | A | G | -0.37 | 0.027 | 1.700E-42 | 0.733 | 3200 | Emilsson | O15394 | 187.791 |
| Plasma | NCR1 | O76036 | rs143981324 | 19 | 55419632 | T | C | 0.362 | 0.042 | 1.300E-17 | 0.904 | 3200 | Emilsson | O76036 | 74.288 |
| Plasma | NEGR1 | Q7Z3B1 | rs2220253 | 1 | 72565460 | T | C | 0.148 | 0.024 | 1.200E-09 | 0.545 | 3200 | Emilsson | Q7Z3B1 | 38.028 |
| Plasma | NLGN2 | Q8NFZ4 | rs150452493 | 17 | 7303808 | C | T | 0.252 | 0.04 | 4.900E-10 | 0.9 | 3200 | Emilsson | Q8NFZ4 | 39.690 |
| Plasma | NMB | P08949 | rs12912342 | 15 | 85248216 | T | C | 0.164 | 0.025 | 7.600E-11 | 0.661 | 3200 | Emilsson | P08949 | 43.034 |
| Plasma | NPNT | Q6UXI9 | rs78213340 | 4 | 106819613 | T | C | 0.314 | 0.049 | 1.700E-10 | 0.93 | 3200 | Emilsson | Q6UXI9 | 41.065 |
| Plasma | NPTXR | O95502 | rs12628473 | 22 | 39240717 | A | G | -1.013 | 0.107 | 4.600E-21 | 0.987 | 3200 | Emilsson | O95502 | 89.630 |
| Plasma | NQO2 | P16083 | rs138616686 | 6 | 3003970 | A | G | 0.435 | 0.025 | 3.800E-64 | 0.626 | 3200 | Emilsson | P16083 | 302.760 |
| Plasma | NTM | Q9P121 | rs2511504 | 11 | 131196396 | T | C | -0.381 | 0.023 | 1.700E-58 | 0.588 | 3200 | Emilsson | Q9P121 | 274.406 |
| Plasma | NTRK3 | Q16288 | rs9944243 | 15 | 88514162 | T | G | -0.221 | 0.035 | 5.200E-10 | 0.879 | 3200 | Emilsson | Q16288 | 39.870 |
| Plasma | OLFM1 | Q99784 | rs11103667 | 9 | 137978360 | C | T | -0.193 | 0.032 | 2.600E-09 | 0.815 | 3200 | Emilsson | Q99784 | 36.376 |
| Plasma | OLFM2 | O95897 | rs56243392 | 19 | 10061960 | A | T | -0.673 | 0.024 | 6.000E-157 | 0.651 | 3200 | Emilsson | O95897 | 786.335 |
| Plasma | OSCAR | Q8IYS5 | rs4442925 | 19 | 54554950 | T | C | 0.138 | 0.024 | 1.300E-08 | 0.455 | 3200 | Emilsson | Q8IYS5 | 33.063 |
| Plasma | OXT | P01178 | rs877172 | 20 | 3049890 | T | G | -0.669 | 0.024 | 5.600E-156 | 0.66 | 3200 | Emilsson | P01178 | 777.016 |
| Plasma | PCBD1 | P61457 | rs72818110 | 10 | 72614896 | A | C | 0.374 | 0.057 | 7.600E-11 | 0.951 | 3200 | Emilsson | P61457 | 43.052 |
| Plasma | PCDH9 | Q9HC56 | rs1927820 | 13 | 67774646 | G | C | -0.266 | 0.033 | 2.200E-15 | 0.846 | 3200 | Emilsson | Q9HC56 | 64.973 |
| Plasma | PCSK9 | Q8NBP7 | rs191448950 | 1 | 55584844 | G | A | 1.069 | 0.113 | 8.400E-21 | 0.989 | 3200 | Emilsson | Q8NBP7 | 89.495 |
| Plasma | PCYOX1 | Q9UHG3 | rs2706762 | 2 | 70488470 | C | T | 0.79 | 0.032 | 1.400E-121 | 0.86 | 3200 | Emilsson | Q9UHG3 | 609.473 |
| Plasma | PDIA3 | P30101 | rs3110081 | 15 | 43995786 | C | T | 0.194 | 0.032 | 1.500E-09 | 0.821 | 3200 | Emilsson | P30101 | 36.754 |
| Plasma | PDLIM4 | P50479 | rs6864922 | 5 | 131616290 | C | T | 0.676 | 0.048 | 1.500E-44 | 0.937 | 3200 | Emilsson | P50479 | 198.340 |
| Plasma | PEBP1 | P30086 | rs76597567 | 12 | 118584885 | A | G | 1.971 | 0.085 | 4.000E-111 | 0.982 | 3200 | Emilsson | P30086 | 537.694 |
| Plasma | PGLYRP2 | Q96PD5 | rs55866012 | 19 | 15578008 | T | G | 0.486 | 0.033 | 1.800E-47 | 0.842 | 3200 | Emilsson | Q96PD5 | 216.893 |
| Plasma | PIP | P12273 | rs4726600 | 7 | 142881540 | G | A | -0.188 | 0.027 | 7.800E-12 | 0.741 | 3200 | Emilsson | P12273 | 48.483 |
| Plasma | PKDCC | Q504Y2 | rs893812 | 2 | 42303114 | C | T | -0.236 | 0.027 | 5.600E-18 | 0.731 | 3200 | Emilsson | Q504Y2 | 76.401 |
| Plasma | PLAT | P00750 | rs77346091 | 8 | 42020158 | T | C | 0.549 | 0.077 | 1.200E-12 | 0.973 | 3200 | Emilsson | P00750 | 50.835 |
| Plasma | PLAUR | Q03405 | rs2302524 | 19 | 44156472 | T | C | 0.22 | 0.031 | 7.900E-13 | 0.82 | 3200 | Emilsson | Q03405 | 50.364 |
| Plasma | PLXNA1 | Q9UIW2 | rs891762 | 3 | 126739012 | G | T | -0.182 | 0.027 | 9.600E-12 | 0.318 | 3200 | Emilsson | Q9UIW2 | 45.438 |
| Plasma | PNLIPRP2 | P54317 | rs7910135 | 10 | 118398046 | C | A | -0.969 | 0.018 | 1.000E-200 | 0.493 | 3200 | Emilsson | P54317 | 2898.028 |
| Plasma | POSTN | Q15063 | rs962462 | 13 | 38089171 | A | G | 0.244 | 0.025 | 8.600E-23 | 0.714 | 3200 | Emilsson | Q15063 | 95.258 |
| Plasma | PRDX6 | P30041 | rs6671141 | 1 | 173446934 | T | G | -0.413 | 0.034 | 8.800E-33 | 0.85 | 3200 | Emilsson | P30041 | 147.551 |
| Plasma | PREP | P48147 | rs1051484 | 6 | 105726036 | C | T | -0.453 | 0.032 | 7.000E-44 | 0.835 | 3200 | Emilsson | P48147 | 200.399 |
| Plasma | PROK2 | Q9HC23 | rs7644362 | 3 | 71829242 | A | G | -1.001 | 0.025 | 1.000E-200 | 0.798 | 3200 | Emilsson | Q9HC23 | 1603.202 |
| Plasma | PRSS57 | Q6UWY2 | rs9304936 | 19 | 689590 | G | C | 0.599 | 0.026 | 7.500E-107 | 0.715 | 3200 | Emilsson | Q6UWY2 | 530.771 |
| Plasma | PSAP | P07602 | rs7086891 | 10 | 73655350 | G | A | 0.619 | 0.025 | 3.600E-122 | 0.709 | 3200 | Emilsson | P07602 | 613.058 |
| Plasma | PSMB1 | P20618 | rs756519 | 6 | 170850862 | G | A | 0.412 | 0.023 | 8.100E-67 | 0.531 | 3200 | Emilsson | P20618 | 320.877 |
| Plasma | PTGR1 | Q14914 | rs112140014 | 9 | 114325849 | G | C | -0.726 | 0.055 | 2.100E-38 | 0.952 | 3200 | Emilsson | Q14914 | 174.240 |
| Plasma | PTPRU | Q92729 | rs2179795 | 1 | 29642318 | G | T | 0.154 | 0.028 | 3.800E-08 | 0.725 | 3200 | Emilsson | Q92729 | 30.250 |
| Plasma | PXDN | Q92626 | rs34008669 | 2 | 1709779 | G | A | 0.158 | 0.023 | 1.800E-11 | 0.502 | 3200 | Emilsson | Q92626 | 47.191 |
| Plasma | PYY | P10082 | rs8074783 | 17 | 42028989 | C | A | 0.157 | 0.026 | 1.200E-09 | 0.641 | 3200 | Emilsson | P10082 | 36.463 |
| Plasma | PZP | P20742 | rs7311982 | 12 | 9314857 | C | T | 0.5 | 0.019 | 2.700E-135 | 0.698 | 3200 | Emilsson | P20742 | 692.521 |
| Plasma | QPCT | Q16769 | rs13027919 | 2 | 37567636 | C | T | 0.271 | 0.024 | 3.500E-28 | 0.538 | 3200 | Emilsson | Q16769 | 127.502 |
| Plasma | RAB6B | Q9NRW1 | rs9813363 | 3 | 133604743 | G | A | 0.278 | 0.032 | 1.200E-17 | 0.825 | 3200 | Emilsson | Q9NRW1 | 75.473 |
| Plasma | RBP4 | P02753 | rs36014035 | 10 | 95360027 | A | C | 0.147 | 0.025 | 7.200E-09 | 0.643 | 3200 | Emilsson | P02753 | 34.574 |
| Plasma | RECQL | P46063 | rs144436375 | 12 | 21515500 | A | C | -0.935 | 0.086 | 9.000E-27 | 0.98 | 3200 | Emilsson | P46063 | 118.202 |
| Plasma | REG3G | Q6UW15 | rs429694 | 2 | 79244586 | A | C | -0.487 | 0.028 | 9.500E-63 | 0.794 | 3200 | Emilsson | Q6UW15 | 302.511 |
| Plasma | RNASE3 | P12724 | rs2771316 | 14 | 21430474 | C | T | 0.214 | 0.028 | 1.200E-14 | 0.723 | 3200 | Emilsson | P12724 | 58.413 |
| Plasma | RNPEP | Q9H4A4 | rs59698324 | 1 | 201965855 | C | T | 0.371 | 0.027 | 3.800E-42 | 0.725 | 3200 | Emilsson | Q9H4A4 | 188.808 |
| Plasma | RSPO4 | Q2I0M5 | rs6056847 | 20 | 1028346 | G | A | -0.172 | 0.023 | 9.000E-14 | 0.575 | 3200 | Emilsson | Q2I0M5 | 55.924 |
| Plasma | S100A7 | P31151 | rs3014860 | 1 | 153314782 | A | G | -1.208 | 0.051 | 1.800E-114 | 0.948 | 3200 | Emilsson | P31151 | 561.040 |
| Plasma | SCUBE1 | Q8IWY4 | rs2744874 | 22 | 43715862 | T | C | -0.27 | 0.036 | 3.800E-14 | 0.866 | 3200 | Emilsson | Q8IWY4 | 56.250 |
| Plasma | SELPLG | Q14242 | rs73191242 | 12 | 109013956 | G | A | 0.209 | 0.03 | 2.100E-12 | 0.795 | 3200 | Emilsson | Q14242 | 48.534 |
| Plasma | SEMA4C | Q9C0C4 | rs112826173 | 2 | 97489654 | A | G | 0.713 | 0.09 | 4.000E-15 | 0.981 | 3200 | Emilsson | Q9C0C4 | 62.762 |
| Plasma | SEMA4D | Q92854 | rs45464494 | 9 | 91994433 | C | T | 1.278 | 0.041 | 1.500E-187 | 0.929 | 3200 | Emilsson | Q92854 | 971.615 |
| Plasma | SERPINA11 | Q86U17 | rs17090881 | 14 | 94913209 | C | A | 0.288 | 0.031 | 1.800E-20 | 0.835 | 3200 | Emilsson | Q86U17 | 86.310 |
| Plasma | SFRP4 | Q6FHJ7 | rs75474297 | 7 | 37973124 | T | A | -0.302 | 0.034 | 2.100E-18 | 0.855 | 3200 | Emilsson | Q6FHJ7 | 78.896 |
| Plasma | SFTPB | P07988 | rs1130866 | 2 | 85893741 | A | G | -0.686 | 0.022 | 3.500E-189 | 0.466 | 3200 | Emilsson | P07988 | 972.306 |
| Plasma | SHANK3 | Q9BYB0 | rs6010042 | 22 | 51098764 | G | A | -0.327 | 0.027 | 6.600E-33 | 0.748 | 3200 | Emilsson | Q9BYB0 | 146.679 |
| Plasma | SHBG | P04278 | rs858519 | 17 | 7531965 | T | C | -0.214 | 0.024 | 2.300E-18 | 0.474 | 3200 | Emilsson | P04278 | 79.507 |
| Plasma | SHMT1 | P34896 | rs8067462 | 17 | 18263571 | C | A | -0.3 | 0.025 | 1.000E-31 | 0.607 | 3200 | Emilsson | P34896 | 144.000 |
| Plasma | SLITRK3 | O94933 | rs62282371 | 3 | 164911694 | T | G | 0.345 | 0.038 | 3.700E-19 | 0.887 | 3200 | Emilsson | O94933 | 82.427 |
| Plasma | SMPD1 | P17405 | rs1050239 | 11 | 6415463 | G | A | 0.31 | 0.026 | 1.800E-31 | 0.715 | 3200 | Emilsson | P17405 | 142.160 |
| Plasma | SOD3 | P08294 | rs2695234 | 4 | 24804238 | G | A | -0.736 | 0.042 | 1.100E-64 | 0.919 | 3200 | Emilsson | P08294 | 307.084 |
| Plasma | SPINK1 | P00995 | rs4705205 | 5 | 147218813 | T | C | -0.212 | 0.025 | 1.500E-17 | 0.573 | 3200 | Emilsson | P00995 | 71.910 |
| Plasma | SPINK5 | Q9NQ38 | rs2052536 | 5 | 147504557 | T | G | -0.152 | 0.026 | 3.300E-09 | 0.349 | 3200 | Emilsson | Q9NQ38 | 34.178 |
| Plasma | SPON2 | Q9BUD6 | rs878323 | 4 | 1169813 | T | G | -0.213 | 0.026 | 1.800E-16 | 0.687 | 3200 | Emilsson | Q9BUD6 | 67.114 |
| Plasma | SRL | Q86TD4 | rs8046884 | 16 | 4269558 | C | G | 0.15 | 0.024 | 6.300E-10 | 0.495 | 3200 | Emilsson | Q86TD4 | 39.063 |
| Plasma | ST6GALNAC6 | Q969X2 | rs183995738 | 9 | 130542640 | C | A | -0.715 | 0.107 | 3.100E-11 | 0.987 | 3200 | Emilsson | Q969X2 | 44.652 |
| Plasma | SULT2A1 | Q06520 | rs296369 | 19 | 48371853 | C | T | 0.36 | 0.033 | 1.700E-27 | 0.843 | 3200 | Emilsson | Q06520 | 119.008 |
| Plasma | TF | P02787 | rs4854760 | 3 | 133498741 | A | G | -0.227 | 0.028 | 6.100E-16 | 0.735 | 3200 | Emilsson | P02787 | 65.726 |
| Plasma | TGFB1 | P01137 | rs1800470 | 19 | 41858921 | A | G | 0.259 | 0.024 | 4.500E-26 | 0.621 | 3200 | Emilsson | P01137 | 116.460 |
| Plasma | THBS4 | P35443 | rs35351529 | 5 | 79390222 | T | C | -0.298 | 0.044 | 2.300E-11 | 0.923 | 3200 | Emilsson | P35443 | 45.870 |
| Plasma | TIMP2 | P16035 | rs2376999 | 17 | 76890864 | A | G | 0.418 | 0.037 | 3.300E-29 | 0.884 | 3200 | Emilsson | P16035 | 127.629 |
| Plasma | TMEM106B | Q9NUM4 | rs10950398 | 7 | 12264871 | A | G | -0.272 | 0.025 | 6.100E-27 | 0.393 | 3200 | Emilsson | Q9NUM4 | 118.374 |
| Plasma | TMEM132D | Q14C87 | rs61943549 | 12 | 130092229 | T | C | -0.486 | 0.055 | 1.000E-18 | 0.948 | 3200 | Emilsson | Q14C87 | 78.081 |
| Plasma | TNFRSF10B | O14763 | rs4871844 | 8 | 22879734 | T | C | -0.143 | 0.026 | 4.300E-08 | 0.658 | 3200 | Emilsson | O14763 | 30.250 |
| Plasma | TNFRSF19 | Q9NS68 | rs3814787 | 13 | 24152370 | G | C | -0.166 | 0.026 | 2.100E-10 | 0.714 | 3200 | Emilsson | Q9NS68 | 40.763 |
| Plasma | TREM1 | Q9NP99 | rs3789204 | 6 | 41254741 | G | T | -0.374 | 0.024 | 1.400E-54 | 0.693 | 3200 | Emilsson | Q9NP99 | 242.840 |
| Plasma | TREM2 | Q9NZC2 | rs114812713 | 6 | 41034000 | G | C | 0.36 | 0.057 | 3.100E-10 | 0.955 | 3200 | Emilsson | Q9NZC2 | 39.889 |
| Plasma | TREML1 | Q86YW5 | rs62396317 | 6 | 41095817 | A | G | 0.211 | 0.038 | 2.900E-08 | 0.878 | 3200 | Emilsson | Q86YW5 | 30.832 |
| Plasma | TXNDC15 | Q96J42 | rs78165052 | 5 | 134250596 | C | T | -0.307 | 0.036 | 3.600E-17 | 0.875 | 3200 | Emilsson | Q96J42 | 72.723 |
| Plasma | TYMP | P19971 | rs131798 | 22 | 50971509 | G | T | 0.204 | 0.03 | 1.800E-11 | 0.773 | 3200 | Emilsson | P19971 | 46.240 |
| Plasma | TYRO3 | Q06418 | rs2289743 | 15 | 41860698 | C | G | -0.161 | 0.027 | 2.100E-09 | 0.691 | 3200 | Emilsson | Q06418 | 35.557 |
| Plasma | UBASH3B | Q8TF42 | rs10502249 | 11 | 122504251 | G | T | 0.187 | 0.027 | 1.100E-11 | 0.712 | 3200 | Emilsson | Q8TF42 | 47.968 |
| Plasma | UGT1A6 | P19224 | rs111741722 | 2 | 234665983 | A | G | 0.265 | 0.027 | 1.800E-22 | 0.681 | 3200 | Emilsson | P19224 | 96.331 |
| Plasma | ULBP3 | Q9BZM4 | rs17054300 | 6 | 150370552 | G | A | 0.171 | 0.028 | 1.700E-09 | 0.762 | 3200 | Emilsson | Q9BZM4 | 37.297 |
| Plasma | UNC5D | Q6UXZ4 | rs6468316 | 8 | 35237788 | T | C | 0.141 | 0.024 | 9.500E-09 | 0.417 | 3200 | Emilsson | Q6UXZ4 | 34.516 |
| Plasma | UST | Q9Y2C2 | rs11155591 | 6 | 149064986 | C | T | 0.149 | 0.026 | 1.200E-08 | 0.685 | 3200 | Emilsson | Q9Y2C2 | 32.842 |
| Plasma | UXS1 | Q8NBZ7 | rs12617748 | 2 | 106836042 | G | A | -0.219 | 0.036 | 1.700E-09 | 0.87 | 3200 | Emilsson | Q8NBZ7 | 37.007 |
| Plasma | VAV1 | P15498 | rs56100731 | 19 | 6857245 | C | T | 0.363 | 0.048 | 5.600E-14 | 0.93 | 3200 | Emilsson | P15498 | 57.191 |
| Plasma | VWA2 | Q5GFL6 | rs11595697 | 10 | 115905775 | C | T | -0.431 | 0.042 | 7.800E-25 | 0.903 | 3200 | Emilsson | Q5GFL6 | 105.307 |
| Plasma | VWC2 | Q2TAL6 | rs79259707 | 7 | 49812564 | C | A | 0.34 | 0.035 | 1.300E-22 | 0.868 | 3200 | Emilsson | Q2TAL6 | 94.367 |
| Plasma | WARS | P23381 | rs941923 | 14 | 100813077 | C | T | 0.255 | 0.029 | 6.600E-18 | 0.766 | 3200 | Emilsson | P23381 | 77.319 |
| Plasma | WFDC1 | Q9HC57 | rs400345 | 16 | 84328494 | C | T | 0.451 | 0.029 | 5.500E-54 | 0.791 | 3200 | Emilsson | Q9HC57 | 241.856 |
| Plasma | WFDC5 | Q8TCV5 | rs35017113 | 20 | 43692684 | C | T | 0.229 | 0.033 | 4.700E-12 | 0.843 | 3200 | Emilsson | Q8TCV5 | 48.155 |
| Plasma | WFIKKN1 | Q96NZ8 | rs11248941 | 16 | 658271 | G | T | -0.203 | 0.028 | 2.300E-13 | 0.732 | 3200 | Emilsson | Q96NZ8 | 52.563 |
| Plasma | WISP2 | O76076 | rs1061098 | 20 | 43356156 | C | T | 0.213 | 0.022 | 4.800E-21 | 0.663 | 3200 | Emilsson | O76076 | 93.738 |
| Plasma | XXYLT1 | Q8NBI6 | rs55947051 | 3 | 194783033 | T | C | -0.248 | 0.033 | 8.600E-14 | 0.841 | 3200 | Emilsson | Q8NBI6 | 56.478 |
| Plasma | ZG16B | Q96DA0 | rs2190809 | 16 | 2880614 | C | T | 0.156 | 0.027 | 1.500E-08 | 0.725 | 3200 | Emilsson | Q96DA0 | 33.383 |
| CSF | 6-Phosphogluconate dehydrogenase | P52209 | rs141107515 | 1 | 10461933 | G | A | 0.9770153 | -0.184683 | 2.032E-02 | 9.82E-20 | 835 | Yang | P52209 | 82.644 |
| CSF | AK1A1 | P14550 | rs2229540 | 1 | 46032311 | G | A | 0.046123 | -0.191302 | 1.203E-02 | 6.12E-57 | 835 | Yang | P14550 | 252.879 |
| CSF | Apo E2 | P02649 | rs71352238 | 19 | 45394336 | G | A | 0.191255 | 0.0225666 | 3.892E-03 | 6.67E-09 | 835 | Yang | P02649 | 33.628 |
| CSF | Apo L1 | O14791 | rs10854688 | 22 | 36653854 | T | C | 0.698962 | -0.0590411 | 1.052E-02 | 2.03E-08 | 835 | Yang | O14791 | 31.468 |
| CSF | ARMEL | Q49AH0 | rs72772417 | 10 | 14873794 | T | C | 0.0440781 | 0.0876585 | 1.032E-02 | 2.06E-17 | 835 | Yang | Q49AH0 | 72.085 |
| CSF | ART | O00253 | rs9928014 | 16 | 67991427 | T | C | 0.9388369 | 0.0836056 | 1.352E-02 | 6.18E-10 | 835 | Yang | O00253 | 38.263 |
| CSF | ARTS1 | Q9NZ08 | rs17482078 | 5 | 96118866 | T | C | 0.188919 | -0.245997 | 1.191E-02 | 8.64E-95 | 835 | Yang | Q9NZ08 | 426.665 |
| CSF | Arylsulfatase A | P15289 | rs6151419 | 22 | 51064915 | G | A | 0.840273 | -0.0670549 | 7.297E-03 | 3.94E-20 | 835 | Yang | P15289 | 84.452 |
| CSF | ASAH2 | Q9NR71 | rs2813302 | 10 | 52012044 | G | A | 0.267403 | -0.0600622 | 5.946E-03 | 5.47E-24 | 835 | Yang | Q9NR71 | 102.030 |
| CSF | ASAHL | Q02083 | rs66498356 | 4 | 76857064 | G | C | 0.718645 | -0.134239 | 7.460E-03 | 2.17E-72 | 835 | Yang | Q02083 | 323.781 |
| CSF | ATS13 | Q76LX8 | rs34265876 | 9 | 136287207 | G | A | 0.0800971 | -0.0810075 | 1.227E-02 | 3.98E-11 | 835 | Yang | Q76LX8 | 43.623 |
| CSF | B7-H1 | Q9NZQ7 | rs7048841 | 9 | 5460801 | T | C | 0.472129 | 0.047533 | 4.464E-03 | 1.78E-26 | 835 | Yang | Q9NZQ7 | 113.386 |
| CSF | B7-H2 | O75144 | rs56124762 | 21 | 45658474 | G | A | 0.238213 | -0.103022 | 6.333E-03 | 1.67E-59 | 835 | Yang | O75144 | 264.639 |
| CSF | BSP | P21815 | rs6855426 | 4 | 88646335 | G | A | 0.719692 | 0.135501 | 2.378E-02 | 1.22E-08 | 835 | Yang | P21815 | 32.461 |
| CSF | BST1 | Q10588 | rs7683000 | 4 | 15647074 | G | A | 0.162342 | -0.179222 | 9.391E-03 | 3.34E-81 | 835 | Yang | Q10588 | 364.254 |
| CSF | C1-Esterase Inhibitor | P05155 | rs11603020 | 11 | 57374332 | G | A | 0.258839 | -0.054944 | 5.765E-03 | 1.56E-21 | 835 | Yang | P05155 | 90.839 |
| CSF | CAPG | P40121 | rs3770102 | 2 | 85637837 | A | C | 0.39397 | 0.0333235 | 5.147E-03 | 9.54E-11 | 835 | Yang | P40121 | 41.913 |
| CSF | Carbonic Anhydrase IV | P22748 | rs62083731 | 17 | 58262406 | T | C | 0.9608785 | -0.10296 | 1.417E-02 | 3.75E-13 | 835 | Yang | P22748 | 52.769 |
| CSF | CATF | Q9UBX1 | rs572846 | 11 | 66331458 | G | A | 0.564462 | -0.0246714 | 4.517E-03 | 4.71E-08 | 835 | Yang | Q9UBX1 | 29.832 |
| CSF | Cathepsin B | P07858 | rs1736065 | 8 | 11685453 | G | A | 0.251177 | 0.0545875 | 5.145E-03 | 2.66E-26 | 835 | Yang | P07858 | 112.584 |
| CSF | Cathepsin H | P09668 | rs2869887 | 15 | 79155033 | A | C | 0.235913 | -0.0744228 | 8.469E-03 | 1.53E-18 | 835 | Yang | P09668 | 77.215 |
| CSF | Cathepsin S | P25774 | rs41271951 | 1 | 150737220 | G | A | 0.0708024 | -0.124607 | 8.779E-03 | 1.00E-45 | 835 | Yang | P25774 | 201.463 |
| CSF | CBPE | P16870 | rs11736871 | 4 | 166336382 | T | C | 0.689751 | 0.0284618 | 4.531E-03 | 3.37E-10 | 835 | Yang | P16870 | 39.450 |
| CSF | CD30 Ligand | P32971 | rs3181370 | 9 | 117665752 | G | A | 0.3998 | -0.0296433 | 4.269E-03 | 3.82E-12 | 835 | Yang | P32971 | 48.217 |
| CSF | CD5L | O43866 | rs6427401 | 1 | 157748564 | G | A | 0.496309 | 0.0593939 | 1.055E-02 | 1.79E-08 | 835 | Yang | O43866 | 31.709 |
| CSF | CDON | Q4KMG0 | rs76617496 | 11 | 125870397 | G | A | 0.0367647 | 0.136466 | 1.243E-02 | 4.63E-28 | 835 | Yang | Q4KMG0 | 120.619 |
| CSF | Chitotriosidase-1 | Q13231 | rs2244385 | 1 | 203191262 | G | C | 0.176985 | -0.28168 | 2.308E-02 | 2.92E-34 | 835 | Yang | Q13231 | 148.965 |
| CSF | CLC7A | Q9BXN2 | rs16909966 | 12 | 10224430 | C | A | 0.0739191 | -0.118182 | 9.037E-03 | 4.46E-39 | 835 | Yang | Q9BXN2 | 171.005 |
| CSF | CNTN2 | Q02246 | rs2242001 | 1 | 205031744 | G | A | 0.842194 | -0.0877676 | 6.055E-03 | 1.30E-47 | 835 | Yang | Q02246 | 210.113 |
| CSF | Coagulation Factor V | P12259 | rs966751 | 1 | 169486141 | G | C | 0.0500668 | -0.106112 | 1.205E-02 | 1.29E-18 | 835 | Yang | P12259 | 77.559 |
| CSF | Coagulation Factor VII | P08709 | rs488703 | 13 | 113770876 | G | A | 0.877248 | -0.165473 | 1.081E-02 | 7.34E-53 | 835 | Yang | P08709 | 234.171 |
| CSF | Collectin Kidney 1 | Q9BWP8 | rs10206003 | 2 | 3672670 | G | A | 0.121677 | 0.204862 | 1.403E-02 | 2.85E-48 | 835 | Yang | Q9BWP8 | 213.127 |
| CSF | complement factor H-related 5 | Q9BXR6 | rs35662416 | 1 | 196967354 | G | A | 0.9780371 | -0.163473 | 2.286E-02 | 8.71E-13 | 835 | Yang | Q9BXR6 | 51.115 |
| CSF | CREL1 | Q96HD1 | rs58020561 | 3 | 10054682 | G | A | 0.28318 | -0.0583374 | 6.826E-03 | 1.27E-17 | 835 | Yang | Q96HD1 | 73.043 |
| CSF | Cripto | P13385 | rs11713041 | 3 | 46770034 | T | C | 0.9412378 | 0.175753 | 2.165E-02 | 4.75E-16 | 835 | Yang | P13385 | 65.897 |
| CSF | Cyclophilin F | P30405 | rs11002931 | 10 | 81094251 | C | A | 0.265292 | -0.0338668 | 3.952E-03 | 1.03E-17 | 835 | Yang | P30405 | 73.445 |
| CSF | Cystatin M | Q15828 | rs610497 | 11 | 65765551 | G | A | 0.229694 | 0.131858 | 7.137E-03 | 3.31E-76 | 835 | Yang | Q15828 | 341.307 |
| CSF | DC-SIGN | Q9NNX6 | rs1010047 | 19 | 7793502 | G | A | 0.541583 | -0.0341856 | 5.379E-03 | 2.08E-10 | 835 | Yang | Q9NNX6 | 40.394 |
| CSF | DERM | Q07507 | rs545833 | 1 | 168689940 | T | C | 0.270126 | 0.0653177 | 6.357E-03 | 9.14E-25 | 835 | Yang | Q07507 | 105.574 |
| CSF | DLL1 | O00548 | rs9348260 | 6 | 170516123 | G | A | 0.315037 | 0.0282914 | 5.094E-03 | 2.79E-08 | 835 | Yang | O00548 | 30.845 |
| CSF | DPP2 | Q9UHL4 | rs6420280 | 9 | 140002501 | T | C | 0.219286 | -0.0574942 | 3.934E-03 | 2.27E-48 | 835 | Yang | Q9UHL4 | 213.580 |
| CSF | ECM1 | Q16610 | rs7002 | 1 | 150445819 | G | A | 0.368704 | -0.0231793 | 3.500E-03 | 3.53E-11 | 835 | Yang | Q16610 | 43.858 |
| CSF | EGF | P01133 | rs10488881 | 4 | 110877416 | T | A | 0.32953 | 0.0353458 | 6.247E-03 | 1.53E-08 | 835 | Yang | P01133 | 32.009 |
| CSF | EMR2 | Q9UHX3 | rs11880837 | 19 | 14808453 | G | A | 0.704746 | 0.0536702 | 5.141E-03 | 1.63E-25 | 835 | Yang | Q9UHX3 | 108.995 |
| CSF | Endocan | Q9NQ30 | rs4865911 | 5 | 54324920 | G | A | 0.38676 | 0.0351592 | 5.706E-03 | 7.17E-10 | 835 | Yang | Q9NQ30 | 37.973 |
| CSF | EphA1 | P21709 | rs7792781 | 7 | 143107026 | T | C | 0.0720993 | -0.126315 | 9.248E-03 | 1.79E-42 | 835 | Yang | P21709 | 186.560 |
| CSF | ERBB1 | P00533 | rs6957408 | 7 | 54923277 | T | C | 0.160252 | -0.0400536 | 6.693E-03 | 2.18E-09 | 835 | Yang | P00533 | 35.808 |
| CSF | Esterase D | P10768 | rs8192887 | 13 | 47362478 | T | C | 0.0965909 | -0.203704 | 1.217E-02 | 7.58E-63 | 835 | Yang | P10768 | 279.980 |
| CSF | FAM3B | P58499 | rs2838014 | 21 | 42697714 | G | C | 0.192361 | 0.246444 | 9.152E-03 | 1.02E-159 | 835 | Yang | P58499 | 725.146 |
| CSF | FAM3D | Q96BQ1 | rs4020782 | 3 | 58648031 | T | C | 0.120173 | -0.0760098 | 1.090E-02 | 3.08E-12 | 835 | Yang | Q96BQ1 | 48.637 |
| CSF | Fas, soluble | P25445 | rs4406737 | 10 | 90759724 | G | A | 0.537259 | 0.0599798 | 4.780E-03 | 4.11E-36 | 835 | Yang | P25445 | 157.437 |
| CSF | FCG2B | P31994 | rs4657041 | 1 | 161478859 | T | C | 0.496423 | 0.32685 | 1.187E-02 | 8.39E-167 | 835 | Yang | P31994 | 757.726 |
| CSF | FGF-19 | O95750 | rs4980680 | 11 | 69523592 | G | A | 0.849931 | -0.0848136 | 8.778E-03 | 4.38E-22 | 835 | Yang | O95750 | 93.352 |
| CSF | FGF-19 | O95750 | rs7480286 | 11 | 69664318 | T | A | 0.643635 | 0.0374192 | 6.763E-03 | 3.15E-08 | 835 | Yang | O95750 | 30.611 |
| CSF | FSTL3 | O95633 | rs2301742 | 19 | 675513 | C | A | 0.446416 | -0.0262986 | 3.936E-03 | 2.36E-11 | 835 | Yang | O95633 | 44.645 |
| CSF | FUT5 | Q11128 | rs78114888 | 19 | 5947688 | G | A | 0.844535 | 0.127027 | 8.159E-03 | 1.20E-54 | 835 | Yang | Q11128 | 242.363 |
| CSF | Galectin-3 | P17931 | rs76426991 | 14 | 55600939 | G | A | 0.9189551 | -0.0878037 | 6.523E-03 | 2.68E-41 | 835 | Yang | P17931 | 181.181 |
| CSF | Galectin-8 | O00214 | rs16833818 | 1 | 236701459 | G | C | 0.672263 | -0.0329971 | 3.678E-03 | 2.94E-19 | 835 | Yang | O00214 | 80.479 |
| CSF | Gelsolin | P06396 | rs76098787 | 9 | 124047836 | T | C | 0.9607843 | 0.0570599 | 8.180E-03 | 3.05E-12 | 835 | Yang | P06396 | 48.653 |
| CSF | Glutathione S-transferase Pi | P09211 | rs4891 | 11 | 67353970 | G | A | 0.352075 | -0.100814 | 5.782E-03 | 4.44E-68 | 835 | Yang | P09211 | 303.994 |
| CSF | GPC5 | P78333 | rs9523318 | 13 | 92035073 | G | A | 0.599515 | 0.0363543 | 5.473E-03 | 3.09E-11 | 835 | Yang | P78333 | 44.119 |
| CSF | GPC5 | P78333 | rs4331227 | 13 | 92485166 | T | C | 0.642857 | 0.0310598 | 5.520E-03 | 1.83E-08 | 835 | Yang | P78333 | 31.665 |
| CSF | GPNMB | Q14956 | rs858274 | 7 | 23294668 | T | C | 0.544177 | -0.114033 | 5.957E-03 | 1.11E-81 | 835 | Yang | Q14956 | 366.455 |
| CSF | GPVI | Q9HCN6 | rs1613662 | 19 | 55536595 | G | A | 0.172987 | -0.096034 | 9.095E-03 | 4.59E-26 | 835 | Yang | Q9HCN6 | 111.504 |
| CSF | Granulysin | P22749 | rs12151621 | 2 | 85934499 | C | A | 0.760187 | 0.211822 | 1.447E-02 | 1.59E-48 | 835 | Yang | P22749 | 214.288 |
| CSF | Growth hormone receptor | P10912 | rs58934290 | 5 | 42640886 | G | A | 0.269282 | 0.0448098 | 7.052E-03 | 2.09E-10 | 835 | Yang | P10912 | 40.379 |
| CSF | Haptoglobin, Mixed Type | P00738 | rs77303550 | 16 | 72079657 | T | C | 0.193266 | 0.711755 | 6.678E-02 | 1.60E-26 | 835 | Yang | P00738 | 113.595 |
| CSF | HCC-4 | O15467 | rs74842203 | 17 | 34304680 | T | C | 0.0216718 | -0.216248 | 3.287E-02 | 4.76E-11 | 835 | Yang | O15467 | 43.275 |
| CSF | Heparin cofactor II | P05546 | rs165818 | 22 | 21122412 | T | C | 0.414943 | 0.0352528 | 5.111E-03 | 5.31E-12 | 835 | Yang | P05546 | 47.570 |
| CSF | HGF | P14210 | rs10248271 | 7 | 81417306 | T | G | 0.77038 | -0.0432409 | 5.888E-03 | 2.08E-13 | 835 | Yang | P14210 | 53.932 |
| CSF | HGFA | Q04756 | rs1203107 | 4 | 3459849 | T | C | 0.105299 | -0.231892 | 1.656E-02 | 1.58E-44 | 835 | Yang | Q04756 | 195.979 |
| CSF | HPLN1 | P10915 | rs4274957 | 5 | 83207463 | G | A | 0.323864 | 0.193551 | 8.263E-03 | 2.52E-121 | 835 | Yang | P10915 | 548.617 |
| CSF | IDUA | P35475 | rs35220088 | 4 | 1010251 | G | C | 0.635088 | 0.0711615 | 5.294E-03 | 3.41E-41 | 835 | Yang | P35475 | 180.699 |
| CSF | IFN-a/b R1 | P17181 | rs12483293 | 21 | 34716789 | C | A | 0.272485 | 0.0237969 | 4.287E-03 | 2.85E-08 | 835 | Yang | P17181 | 30.807 |
| CSF | IGF-II receptor | P11717 | rs628031 | 6 | 160560845 | G | A | 0.625083 | -0.0348801 | 4.232E-03 | 1.68E-16 | 835 | Yang | P11717 | 67.945 |
| CSF | IL-1 R AcP | Q9NPH3 | rs9813227 | 3 | 190340177 | C | A | 0.847638 | 0.161939 | 8.814E-03 | 2.20E-75 | 835 | Yang | Q9NPH3 | 337.532 |
| CSF | IL-1 R4 | Q01638 | rs10178436 | 2 | 102926511 | G | A | 0.463667 | 0.310681 | 1.055E-02 | 1.71E-190 | 835 | Yang | Q01638 | 866.696 |
| CSF | IL-1 sRI | P14778 | rs6722640 | 2 | 102661613 | T | A | 0.420888 | 0.0362003 | 4.040E-03 | 3.22E-19 | 835 | Yang | P14778 | 80.298 |
| CSF | IL-1 sRII | P27930 | rs7561460 | 2 | 102617204 | G | A | 0.395264 | -0.0374856 | 6.581E-03 | 1.23E-08 | 835 | Yang | P27930 | 32.444 |
| CSF | IL-16 | Q14005 | rs17875509 | 15 | 81590775 | G | C | 0.9034091 | -0.0699534 | 6.417E-03 | 1.15E-27 | 835 | Yang | Q14005 | 118.820 |
| CSF | IL-17 sR | Q96F46 | rs2286951 | 22 | 17612744 | G | A | 0.794396 | 0.0628858 | 7.619E-03 | 1.53E-16 | 835 | Yang | Q96F46 | 68.125 |
| CSF | IL-18 Ra | Q13478 | rs1362350 | 2 | 102951798 | G | C | 0.509699 | 0.143705 | 5.240E-03 | 1.33E-165 | 835 | Yang | Q13478 | 752.213 |
| CSF | IL-22 | Q9GZX6 | rs11177138 | 12 | 68652776 | G | A | 0.806462 | -0.0709875 | 4.929E-03 | 4.94E-47 | 835 | Yang | Q9GZX6 | 207.451 |
| CSF | IL-34 | Q6ZMJ4 | rs36097154 | 16 | 70662816 | G | A | 0.662099 | -0.0481883 | 3.711E-03 | 1.51E-38 | 835 | Yang | Q6ZMJ4 | 168.575 |
| CSF | IL-6 sRa | P08887 | rs4129267 | 1 | 154426264 | T | C | 0.409606 | 0.11181 | 5.121E-03 | 1.14E-105 | 835 | Yang | P08887 | 476.661 |
| CSF | IL-9 | P15248 | rs2069885 | 5 | 135228165 | G | A | 0.876923 | -0.11493 | 9.816E-03 | 1.16E-31 | 835 | Yang | P15248 | 137.084 |
| CSF | IL-9 | P15248 | rs7711775 | 5 | 135270062 | A | C | 0.0339041 | 0.140559 | 2.484E-02 | 1.53E-08 | 835 | Yang | P15248 | 32.018 |
| CSF | ILT-2 | Q8NHL6 | rs2114511 | 19 | 55145093 | G | C | 0.9331984 | -0.229846 | 1.516E-02 | 6.71E-52 | 835 | Yang | Q8NHL6 | 229.766 |
| CSF | Kallikrein 11 | Q9UBX7 | rs2691258 | 19 | 51517286 | G | A | 0.417184 | 0.0826901 | 6.671E-03 | 2.77E-35 | 835 | Yang | Q9UBX7 | 153.644 |
| CSF | kallikrein 8 | O60259 | rs2659091 | 19 | 51446530 | G | A | 0.624659 | -0.0465902 | 5.237E-03 | 5.73E-19 | 835 | Yang | O60259 | 79.158 |
| CSF | Layilin | Q6UX15 | rs674230 | 11 | 111437887 | G | A | 0.648199 | -0.077456 | 6.595E-03 | 7.59E-32 | 835 | Yang | Q6UX15 | 137.921 |
| CSF | LEG9 | O00182 | rs3794195 | 17 | 25959355 | G | A | 0.216892 | -0.0261146 | 3.876E-03 | 1.61E-11 | 835 | Yang | O00182 | 45.395 |
| CSF | LRP8 | Q14114 | rs12031155 | 1 | 53714139 | T | C | 0.389222 | 0.0508676 | 5.865E-03 | 4.20E-18 | 835 | Yang | Q14114 | 75.227 |
| CSF | Luteinizing hormone | P01215, P01229 | rs75287599 | 19 | 49517140 | T | C | 0.0732695 | -0.189818 | 1.950E-02 | 2.11E-22 | 835 | Yang | P01215, P01229 | 94.795 |
| CSF | LY86 | O95711 | rs7757934 | 6 | 6578927 | G | A | 0.272029 | -0.0374461 | 4.089E-03 | 5.33E-20 | 835 | Yang | O95711 | 83.852 |
| CSF | Lysozyme | P61626 | rs57954211 | 12 | 69748397 | C | A | 0.567831 | -0.0811215 | 4.882E-03 | 5.39E-62 | 835 | Yang | P61626 | 276.070 |
| CSF | MIA | Q16674 | rs13108 | 19 | 41271104 | G | A | 0.0841683 | 0.0799671 | 1.278E-02 | 3.96E-10 | 835 | Yang | Q16674 | 39.131 |
| CSF | MIC-1 | Q99988 | rs7251610 | 19 | 18489734 | T | C | 0.316818 | -0.0465713 | 6.415E-03 | 3.87E-13 | 835 | Yang | Q99988 | 52.709 |
| CSF | MIP-5 | Q16663 | rs7208990 | 17 | 34329475 | G | C | 0.0627178 | 0.145186 | 1.065E-02 | 2.48E-42 | 835 | Yang | Q16663 | 185.912 |
| CSF | MMEL1 | Q495T6 | rs10909839 | 1 | 2708430 | G | A | 0.672032 | 0.0914117 | 4.918E-03 | 3.98E-77 | 835 | Yang | Q495T6 | 345.531 |
| CSF | MMP-2 | P08253 | rs1347653 | 16 | 55505040 | A | C | 0.180508 | -0.0317887 | 4.160E-03 | 2.15E-14 | 835 | Yang | P08253 | 58.389 |
| CSF | MMP-8 | P22894 | rs1939020 | 11 | 102590717 | T | G | 0.9176667 | 0.171833 | 1.087E-02 | 2.69E-56 | 835 | Yang | P22894 | 249.933 |
| CSF | MPIF-1 | P55773 | rs76480185 | 17 | 34350933 | T | G | 0.0241287 | -0.0876615 | 1.307E-02 | 2.00E-11 | 835 | Yang | P55773 | 44.968 |
| CSF | MSP | P26927 | rs2172252 | 3 | 49678307 | T | A | 0.30672 | -0.267813 | 1.320E-02 | 1.57E-91 | 835 | Yang | P26927 | 411.688 |
| CSF | N-terminal pro-BNP | P16860 | rs12406383 | 1 | 11921993 | C | A | 0.727338 | 0.0522893 | 5.866E-03 | 4.92E-19 | 835 | Yang | P16860 | 79.458 |
| CSF | NADPH-P450 Oxidoreductase | P16435 | rs1057868 | 7 | 75615006 | T | C | 0.259506 | -0.031595 | 4.470E-03 | 1.56E-12 | 835 | Yang | P16435 | 49.967 |
| CSF | NAGK | Q9UJ70 | rs9636398 | 2 | 71288817 | G | A | 0.824019 | -0.030724 | 4.937E-03 | 4.86E-10 | 835 | Yang | Q9UJ70 | 38.735 |
| CSF | NET4 | Q9HB63 | rs17287608 | 12 | 96119016 | C | A | 0.159532 | -0.062989 | 6.099E-03 | 5.32E-25 | 835 | Yang | Q9HB63 | 106.645 |
| CSF | NID2 | Q14112 | rs6572807 | 14 | 52480621 | G | A | 0.273453 | -0.0351089 | 3.724E-03 | 4.18E-21 | 835 | Yang | Q14112 | 88.887 |
| CSF | Nidogen | P14543 | rs2031487 | 1 | 236175339 | G | A | 0.533793 | 0.0214523 | 3.543E-03 | 1.40E-09 | 835 | Yang | P14543 | 36.664 |
| CSF | NRP1 | O14786 | rs10827216 | 10 | 33509513 | G | A | 0.528466 | -0.0224791 | 3.888E-03 | 7.39E-09 | 835 | Yang | O14786 | 33.430 |
| CSF | OLR1 | P78380 | rs11611438 | 12 | 10318409 | G | C | 0.9215097 | -0.0724723 | 1.029E-02 | 1.88E-12 | 835 | Yang | P78380 | 49.611 |
| CSF | Osteocalcin | P02818 | rs9943105 | 1 | 156245245 | T | C | 0.502329 | -0.0246995 | 4.518E-03 | 4.59E-08 | 835 | Yang | P02818 | 29.881 |
| CSF | Osteopontin | P10451 | rs36018630 | 4 | 88774669 | T | C | 0.203083 | 0.0389893 | 6.554E-03 | 2.70E-09 | 835 | Yang | P10451 | 35.389 |
| CSF | P-Selectin | P16109 | rs6678795 | 1 | 169533266 | G | A | 0.503672 | 0.0730929 | 7.791E-03 | 6.53E-21 | 835 | Yang | P16109 | 88.006 |
| CSF | PAFAH | Q13093 | rs6907892 | 6 | 46625886 | T | C | 0.361759 | 0.100342 | 6.013E-03 | 1.61E-62 | 835 | Yang | Q13093 | 278.486 |
| CSF | PCSK7 | Q16549 | rs8521 | 11 | 117067699 | G | A | 0.267465 | -0.0886385 | 4.897E-03 | 3.13E-73 | 835 | Yang | Q16549 | 327.646 |
| CSF | PCSK9 | Q8NBP7 | rs499718 | 1 | 55512549 | T | C | 0.21409 | -0.0901128 | 7.519E-03 | 4.27E-33 | 835 | Yang | Q8NBP7 | 143.636 |
| CSF | PDGF Rb | P09619 | rs3776081 | 5 | 149532107 | G | A | 0.299245 | -0.16661 | 8.466E-03 | 3.27E-86 | 835 | Yang | P09619 | 387.258 |
| CSF | Periostin | Q15063 | rs9547908 | 13 | 38069809 | T | C | 0.674465 | -0.0758963 | 7.700E-03 | 6.38E-23 | 835 | Yang | Q15063 | 97.165 |
| CSF | PIGR | P01833 | rs2275529 | 1 | 207119853 | C | A | 0.705745 | 0.108083 | 1.433E-02 | 4.70E-14 | 835 | Yang | P01833 | 56.851 |
| CSF | PLXC1 | O60486 | rs2361355 | 12 | 94630029 | G | A | 0.437084 | -0.0355831 | 5.958E-03 | 2.34E-09 | 835 | Yang | O60486 | 35.671 |
| CSF | PPAC | P24666 | rs57542652 | 2 | 228088 | G | A | 0.677473 | 0.236577 | 8.271E-03 | 6.02E-180 | 835 | Yang | P24666 | 818.180 |
| CSF | Prekallikrein | P03952 | rs2304595 | 4 | 187172280 | G | A | 0.607477 | 0.0392912 | 6.571E-03 | 2.23E-09 | 835 | Yang | P03952 | 35.759 |
| CSF | PSMA | Q04609 | rs138800869 | 11 | 49038966 | G | A | 0.9587525 | 0.122141 | 1.352E-02 | 1.67E-19 | 835 | Yang | Q04609 | 81.590 |
| CSF | REG4 | Q9BYZ8 | rs2994816 | 1 | 120371217 | G | A | 0.245825 | 0.0494164 | 7.404E-03 | 2.49E-11 | 835 | Yang | Q9BYZ8 | 44.540 |
| CSF | Semaphorin 3E | O15041 | rs2535371 | 7 | 83147251 | C | A | 0.819612 | -0.176884 | 1.430E-02 | 3.77E-35 | 835 | Yang | O15041 | 153.031 |
| CSF | sFRP-3 | Q92765 | rs78177114 | 2 | 183730429 | G | A | 0.0713807 | 0.0732512 | 9.747E-03 | 5.67E-14 | 835 | Yang | Q92765 | 56.482 |
| CSF | sICAM-1 | P05362 | rs5498 | 19 | 10395683 | G | A | 0.424817 | -0.170336 | 5.079E-03 | 1.00E-200 | 835 | Yang | P05362 | 1124.840 |
| CSF | SIG14 | Q08ET2 | rs7250849 | 19 | 52158316 | A | C | 0.118839 | -0.213572 | 1.418E-02 | 3.01E-51 | 835 | Yang | Q08ET2 | 226.778 |
| CSF | Siglec-3 | P20138 | rs3865444 | 19 | 51727962 | C | A | 0.701597 | -0.160134 | 7.158E-03 | 7.37E-111 | 835 | Yang | P20138 | 500.507 |
| CSF | Siglec-7 | Q9Y286 | rs2075803 | 19 | 51628529 | G | A | 0.561585 | 0.041326 | 6.635E-03 | 4.72E-10 | 835 | Yang | Q9Y286 | 38.790 |
| CSF | Siglec-9 | Q9Y336 | rs2673908 | 19 | 51627766 | A | C | 0.395381 | 1.12657 | 3.155E-02 | 1.00E-200 | 835 | Yang | Q9Y336 | 1275.172 |
| CSF | sL-Selectin | P14151 | rs72712053 | 1 | 169640642 | T | G | 0.759763 | -0.0589202 | 4.982E-03 | 2.86E-32 | 835 | Yang | P14151 | 139.855 |
| CSF | SLAMF7 | Q9NQ25 | rs3766374 | 1 | 160720554 | G | A | 0.717096 | -0.0967191 | 7.610E-03 | 5.28E-37 | 835 | Yang | Q9NQ25 | 161.514 |
| CSF | sLeptin R | P48357 | rs6665672 | 1 | 66069020 | G | A | 0.814652 | -0.2352 | 7.491E-03 | 1.00E-200 | 835 | Yang | P48357 | 985.843 |
| CSF | Spondin-1 | Q9HCB6 | rs7935294 | 11 | 13963974 | C | A | 0.430194 | 0.0274456 | 4.330E-03 | 2.31E-10 | 835 | Yang | Q9HCB6 | 40.184 |
| CSF | SREC-I | Q14162 | rs62088045 | 17 | 1545333 | T | C | 0.0338245 | 0.154818 | 1.439E-02 | 5.32E-27 | 835 | Yang | Q14162 | 115.775 |
| CSF | sTie-1 | P35590 | rs3768046 | 1 | 43766426 | G | A | 0.635389 | -0.0291955 | 5.211E-03 | 2.11E-08 | 835 | Yang | P35590 | 31.394 |
| CSF | Tenascin | P24821 | rs72758637 | 9 | 117805201 | G | C | 0.157664 | 0.21094 | 9.067E-03 | 9.98E-120 | 835 | Yang | P24821 | 541.270 |

| CSF | Testican-2 | Q92563 | rs1245546 | 10 | 73846826 | T | C | 0.484828 | -0.050451 | 4.975E-03 | 3.63E-24 | 835 | Yang | Q92563 | 102.844 |
| --- | --- | --- | --- | --- | --- | --- | --- | --- | --- | --- | --- | --- | --- | --- | --- |
| CSF | TFPI | P10646 | rs13035938 | 2 | 188390112 | G | A | 0.262887 | 0.0293635 | 5.241E-03 | 2.11E-08 | 835 | Yang | P10646 | 31.387 |
| CSF | TIG2 | Q99969 | rs10282458 | 7 | 150045302 | G | A | 0.728883 | 0.0192769 | 3.384E-03 | 1.23E-08 | 835 | Yang | Q99969 | 32.445 |
| CSF | TIMD3 | Q8TDQ0 | rs6874178 | 5 | 156530149 | T | A | 0.816566 | 0.0909462 | 7.012E-03 | 1.79E-38 | 835 | Yang | Q8TDQ0 | 168.241 |
| CSF | TNFSF15 | O95150 | rs6478109 | 9 | 117568766 | G | A | 0.714524 | 0.0581364 | 5.208E-03 | 6.19E-29 | 835 | Yang | O95150 | 124.612 |
| CSF | TPSB2 | P20231 | rs1054648 | 16 | 1271471 | T | C | 0.104447 | 0.457599 | 2.300E-02 | 4.71E-88 | 835 | Yang | P20231 | 395.718 |
| CSF | TrATPase | P13686 | rs8112083 | 19 | 11707266 | G | A | 0.284775 | 0.045859 | 5.193E-03 | 1.05E-18 | 835 | Yang | P13686 | 77.971 |
| CSF | TSG-6 | P98066 | rs6433375 | 2 | 152208657 | G | A | 0.522004 | 0.0521653 | 7.445E-03 | 2.44E-12 | 835 | Yang | P98066 | 49.093 |
| CSF | TSP4 | P35443 | rs2438638 | 5 | 79390200 | G | A | 0.650216 | 0.0395023 | 5.044E-03 | 4.80E-15 | 835 | Yang | P35443 | 61.343 |
| CSF | ULBP-3 | Q9BZM4 | rs12661513 | 6 | 150373839 | C | A | 0.865886 | -0.0677042 | 5.424E-03 | 9.36E-36 | 835 | Yang | Q9BZM4 | 155.801 |
| CSF | Vitronectin | P04004 | rs708100 | 17 | 26688663 | G | A | 0.525384 | -0.41848 | 1.392E-02 | 1.51E-198 | 835 | Yang | P04004 | 903.737 |
| CSF | vWF | P04275 | rs1558325 | 12 | 6289108 | G | A | 0.558882 | 0.0374385 | 6.434E-03 | 5.93E-09 | 835 | Yang | P04275 | 33.859 |
| CSF | WFKN2 | Q8TEU8 | rs11868519 | 17 | 48920101 | T | C | 0.654996 | 0.130114 | 7.548E-03 | 1.36E-66 | 835 | Yang | Q8TEU8 | 297.171 |
| CSF | YKL-40 | P36222 | rs880633 | 1 | 203152801 | G | A | 0.500681 | 0.195833 | 6.741E-03 | 1.41E-185 | 835 | Yang | P36222 | 844.072 |

**Supplementary Table 2 Genetic instruments of SLE for bidirectional MR**

| Other allele | Effect allele | beta | se | SNP | pval | F |
| --- | --- | --- | --- | --- | --- | --- |
| T | G | 0.274437 | 0.0423755 | rs4661543 | 9.40E-11 | 41.94262961 |
| C | A | 0.336472 | 0.0464854 | rs6679677 | 4.55E-13 | 52.39196829 |
| G | A | 0.198851 | 0.0289651 | rs6671847 | 6.64E-12 | 47.13087409 |
| A | G | -0.24686 | 0.0309918 | rs10912578 | 1.65E-15 | 63.44652606 |
| C | T | 0.223144 | 0.0339693 | rs4916215 | 5.07E-11 | 43.15163271 |
| C | T | 0.81093 | 0.0498642 | rs17849501 | 1.81E-59 | 264.4776777 |
| T | C | -0.328504 | 0.0578595 | rs12094036 | 1.37E-08 | 32.23531894 |
| T | C | -0.616186 | 0.104778 | rs34703115 | 4.08E-09 | 34.58465476 |
| C | T | 0.18633 | 0.0323703 | rs268124 | 8.60E-09 | 33.13386659 |
| G | T | -0.562119 | 0.0290336 | rs13019891 | 1.65E-83 | 374.8476147 |
| C | T | 0.261365 | 0.045245 | rs2459611 | 7.62E-09 | 33.36980543 |
| C | T | -0.559616 | 0.0326791 | rs4274624 | 9.73E-66 | 293.2513845 |
| G | T | -0.231112 | 0.0412056 | rs10048743 | 2.04E-08 | 31.45810134 |
| C | T | -0.248461 | 0.0424835 | rs10200680 | 4.96E-09 | 34.20391984 |
| A | C | 0.587787 | 0.0429292 | rs2573219 | 1.13E-42 | 187.4711151 |
| A | G | 0.620577 | 0.0492727 | rs9852014 | 2.26E-36 | 158.6275546 |
| G | T | -0.328504 | 0.0401497 | rs1464446 | 2.79E-16 | 66.94477944 |
| C | T | -0.174353 | 0.027787 | rs13136219 | 3.50E-10 | 39.37091669 |
| C | T | 0.378436 | 0.0603977 | rs4388254 | 3.71E-10 | 39.25943866 |
| C | A | -0.71335 | 0.0781665 | rs1078324 | 7.11E-20 | 83.28446466 |
| T | C | 0.277632 | 0.03174 | rs6889239 | 2.19E-18 | 76.51123259 |
| T | C | -0.223144 | 0.0292964 | rs2431697 | 2.60E-14 | 58.01521611 |
| C | T | 0.928219 | 0.0689573 | rs150180633 | 2.66E-41 | 181.1925808 |
| G | T | -0.673345 | 0.120793 | rs12524498 | 2.48E-08 | 31.0736124 |
| A | G | 0.928219 | 0.0432319 | rs389884 | 2.92E-102 | 460.9908042 |
| C | T | 0.457425 | 0.0351961 | rs9274357 | 1.28E-38 | 168.9081898 |
| G | A | 0.277632 | 0.0509208 | rs57844307 | 4.97E-08 | 29.72683052 |
| C | T | -0.207014 | 0.0296891 | rs7768653 | 3.11E-12 | 48.6189269 |
| C | T | 0.65752 | 0.0755941 | rs58721818 | 3.38E-18 | 75.6557835 |
| C | T | 0.587787 | 0.041539 | rs35000415 | 1.86E-45 | 200.2294151 |
| G | C | 0.277632 | 0.0320694 | rs2736332 | 4.83E-18 | 74.94753854 |
| G | T | -0.350657 | 0.0286208 | rs7823055 | 1.64E-34 | 150.1071876 |
| G | A | -0.18633 | 0.0287118 | rs7097397 | 8.60E-11 | 42.11576829 |
| C | T | 0.182322 | 0.0332532 | rs7899626 | 4.19E-08 | 30.06154268 |
| A | G | -0.223144 | 0.0335647 | rs58688157 | 2.97E-11 | 44.19823106 |
| A | G | 0.18633 | 0.0280198 | rs353608 | 2.93E-11 | 44.22170731 |
| C | T | -0.71335 | 0.124134 | rs73050535 | 9.11E-09 | 33.02355559 |
| A | G | -0.162519 | 0.0294736 | rs597808 | 3.51E-08 | 30.40478329 |

| G | A | 0.582216 | 0.0399866 | rs1143679 | 5.03E-48 | 212.0016865 |
| --- | --- | --- | --- | --- | --- | --- |
| A | G | -0.314711 | 0.0375683 | rs13332649 | 5.43E-17 | 70.17473145 |
| G | A | 0.470004 | 0.0840342 | rs143123127 | 2.23E-08 | 31.28174476 |
| G | A | -0.235722 | 0.0324266 | rs35251378 | 3.61E-13 | 52.84420952 |
| G | A | -0.314711 | 0.0574903 | rs73068668 | 4.40E-08 | 29.96640854 |
| G | A | 0.262364 | 0.0345055 | rs3747093 | 2.88E-14 | 57.8138428 |

**Supplementary Table 3 Heterogeneity analysis on proteins with two or more instruments**

| Tissue | UniProt ID | protein | nSNP | method | Q | Q_pval |
| --- | --- | --- | --- | --- | --- | --- |
| Plasma | A0A024RDE1; Q14515; B7ZB68; Q8N4S1 | SPARCL1 | 2 | IVW | 0.018053568 | 0.893115092 |
| Plasma | P29622; A0A024R6I9 | SERPINA4 | 2 | IVW | 0.006313134 | 0.93667054 |
| Plasma | P78333 | GPC5 | 2 | IVW | 1.571885288 | 0.209933435 |
| CSF | P15248 | IL-9 | 2 | IVW | 0.221339609 | 0.638021093 |
| CSF | P78333 | GPC5 | 2 | IVW | 2.155805023 | 0.142031985 |

**Supplementary Table 4 MR results of proteins and SLE for external validatio**

| Tissue | Exposure | Sources of exposure | nSNP | Method | Beta | SE | P | Sources of outcome |
| --- | --- | --- | --- | --- | --- | --- | --- | --- |
| Plasma | ICAM1 | deCODE (PMID:34857953) | 43 | IVW | -0.05751619 | 0.051557039 | 0.264600387 | Finngen_R10 |
|  |  |  | 45 |  | -0.123520116 | 0.052238207 | 0.01805174 | GCST90018917(PMID:34594039) |
|  |  | GCST90162054(PMID:36168886) | 2 |  | -0.17293497 | 0.286482664 | 0.546077037 | Finngen_R10 |
|  |  |  | 4 |  | -0.063186347 | 0.046036574 | 0.169900072 | GCST90018917(PMID:34594039) |
|  | PPP3CA; PPP3R1 | Same | 2 | IVW | 0.055577929 | 0.044001609 | 0.2065574 | GCST90018917(PMID:34594039) |
|  |  |  |  |  | 0.03648064 | 0.040797216 | 0.3712179 | Finngen_R10 |
| CSF | FCG2B | Same | 1 | Wald ratio | -0.31857427 | 0.132157565 | 0.015927876 | Finngen_R10 |
|  |  |  |  |  | -0.523175769 | 0.150221814 | 0.000496406 | GCST90018917(PMID:34594039) |
|  | sICAM-1 |  |  |  | -0.381051569 | 0.255775056 | 0.136279001 | Finngen_R10 |
|  |  |  |  |  | -0.477879016 | 0.27122863 | 0.07808539 | GCST90018917(PMID:34594039) |
|  | NID2 |  |  |  | 0.010458488 | 1.38754276 | 0.993986069 | Finngen_R10 |
|  |  |  |  |  | -0.487055989 | 1.498195614 | 0.74510913 | GCST90018917(PMID:34594039) |
|  | N-terminal pro-BNP |  |  |  | 0.687857745 | 0.960739578 | 0.474011782 | Finngen_R10 |
|  |  |  |  |  | 0.233317333 | 1.076702117 | 0.828445055 | GCST90018917(PMID:34594039) |
|  | Layilin |  |  |  | 0.772878796 | 0.655689682 | 0.238507105 | Finngen_R10 |
|  |  |  |  |  | 0.151053501 | 0.994112787 | 0.879227908 | GCST90018917(PMID:34594039) |
|  | Prekallikrein |  |  |  | -0.741290671 | 1.105387466 | 0.502465101 | Finngen_R10 |
|  |  |  |  |  | -0.860243515 | 1.188561306 | 0.469207726 | GCST90018917(PMID:34594039) |

**Supplementary Table 5 Eighteen medications for SLE and their corresponding drug targets**

| **Medication** | **Target** | **Gene Name** | **UniProt ID** | **Pharmacological Action** | **Action** |
| --- | --- | --- | --- | --- | --- |
| **hydroxychloroquine** | Toll-like receptor 7 | TLR7 | Q9NYK1 | Yes | Antagonist |
|  | Toll-like receptor 9 | TLR9 | Q9NR96 | Yes | Antagonist |
|  | DNA | / | / | Yes | cross-linking/alkylation |
|  | Angiotensin-converting enzyme 2 | ACE2 | Q9BYF1 | Unknown | modulator |
| **Glucocorticoid** | | | | | |
| **Prednisone** | glucocorticoid receptor | NR3C1 | P04150 | Yes | agonist |
| **Methylprednisolone** | Glucocorticoid receptor | NR3C1 | P04150 | Yes | agonist |
|  | Annexin A1 | ANXA1 | P04083 | Unknown | agonist |
| **Prednisolone** | Glucocorticoid receptor | NR3C1 | P04150 | Yes | agonist |
| **Dexamethasone** | Glucocorticoid receptor | NR3C1 | P04150 | Yes | agonist |
|  | Nuclear receptor subfamily 0 group B member 1 | NR0B1 | P51843 | Unknown | stimulator |
|  | Annexin A1 | ANXA1 | P04083 | Unknown | agonist |
|  | Nitric oxide synthase, inducible | NOS2 | P35228 | Unknown | negative modulator |
|  | Nuclear receptor subfamily 1 group I member 2 | NR1I2 | O75469 | Unknown | agonist |
| **NSAIDS** | | | | | |
| **naproxen** | Prostaglandin G/H synthase 1 | PTGS1 | P23219 | Yes | Inhibitor |
|  | Prostaglandin G/H synthase 2 | PTGS2 | P35354 | Yes | Inhibitor |
|  | Peptostreptococcal albumin-binding protein | pab | Q51911 | Unknown | Inhibitor |
| **ibuprofen** | Prostaglandin G/H synthase 2 | PTGS2 | P35354 | Yes | Inhibitor |
|  | Prostaglandin G/H synthase 1 | PTGS1 | P23219 | Yes | Inhibitor |
|  | Apoptosis regulator Bcl-2 | BCL2 | P10415 | Unknown | modulator |
|  | Thrombomodulin | THBD | P07204 | Unknown | inducer |
|  | Fatty acid-binding protein, intestinal | FABP2 | P12104 | Unknown | binder |
|  | Peroxisome proliferator-activated receptor gamma | PPARG | P37231 | Unknown | activator |
|  | Cystic fibrosis transmembrane conductance regulator | CFTR | P13569 | Unknown | inhibitor |
|  | Peroxisome proliferator-activated receptor alpha | PPARA | Q07869 | Unknown | activator |
|  | Platelet glycoprotein Ib alpha chain | GP1BA | P07359 | Unknown | inducer |
|  | Protein S100-A7 | S100A7 | P31151 | Unknown | inducer |
| **aspirin** | Prostaglandin G/H synthase 1 | PTGS1 | P23219 | Yes | inhibitor |
|  | Prostaglandin G/H synthase 2 | PTGS2 | P35354 | Yes | inhibitor |
|  | Aldo-keto reductase family 1 member C1 | AKR1C1 | Q04828 | Unknown | inhibitor |
|  | 5'-AMP-activated protein kinase | / | Q13131; P54646; Q9Y478; O43741; P54619; Q9UGJ0; Q9UGI9 | Unknown | activator |
|  | Endothelin-1 receptor | EDNRA | P25101 | Unknown | inhibitor |
|  | Cellular tumor antigen p53 | TP53 | P04637 | Unknown | inducer |
|  | 78 kDa glucose-regulated protein | HSPA5 | P11021 | Unknown | inhibitor binder |
|  | Ribosomal protein S6 kinase alpha-3 | RPS6KA3 | P51812 | Unknown | inhibitor |
|  | NF-kappa-B inhibitor alpha | NFKBIA | P25963 | Unknown | inhibitor |
|  | Tumor necrosis factor-inducible gene 6 protein | TNFAIP6 | P98066 | Unknown | inhibitor down regulator |
|  | Caspase-1 | CASP1 | P29466 | Unknown | inhibitor down regulator |
|  | Caspase-3 | CASP3 | P42574 | Unknown | inhibitor down regulator |
|  | Inhibitor of nuclear factor kappa-B kinase subunit beta | IKBKB | O14920 | Unknown | Not Available |
|  | Extracellular signal-regulated kinase (ERK) | / | Q8TD08; P27361; P31152; Q16659; Q13164 | Unknown | Not Available |
|  | G1/S-specific cyclin-D1 | CCND1 | P24385 | Unknown | down regulator |
|  | Myc proto-oncogene protein | MYC | P01106 | Unknown | down regulator |
|  | Proliferating cell nuclear antigen | PCNA | P12004 | Unknown | down regulator |
|  | Cyclin A | / | / | Unknown | down regulator |
|  | Sialidase-1 | NEU1 | Q99519 | Unknown | inhibitor |
| **Cellular toxic drugs** | | | | | |
| **Azathioprine** | Ras-related C3 botulinum toxin substrate 1 | RAC1 | P63000 | Unknown | modulator |
| **Cyclophosphamide** | DNA | / | / | Yes | cross-linking/alkylation |
|  | Nuclear receptor subfamily 1 group I member 2 | NR1I2 | O75469 | Unknown | Not Available |
| **cyclosporine** | Calcium signal-modulating cyclophilin ligand | CAMLG | P49069 | Yes | binder |
|  | Calcineurin subunit B type 2 | PPP3R2 | Q96LZ3 | Yes | inhibitor |
|  | Peptidyl-prolyl cis-trans isomerase A | PPIA | P62937 | Yes | inhibitor binder |
|  | Peptidyl-prolyl cis-trans isomerase F, mitochondrial | PPIF | P30405 | Yes | binder |
| **methotrexate** | Thymidylate synthase | TYMS | P04818 | Yes | inhibitor |
|  | Bifunctional purine biosynthesis protein PURH | ATIC | P31939 | Yes | inhibitor |
|  | Dihydrofolate reductase | DHFR | P00374 | Yes | inhibitor |
| **mycophenolate** | Inosine-5'-monophosphate dehydrogenase 1 | IMPDH2 | P12268 | Yes | inhibitor |
|  | Inosine-5'-monophosphate dehydrogenase 2 | IMPDH1 | P20839 | Yes | inhibitor |
| **mycophenolate** | B-lymphocyte antigen CD20 | MS4A1 | P11836 | Yes | antibody modulator |
| **Belimumab** | Tumor necrosis factor ligand superfamily member 13B | TNFSF13B | Q9Y275 | Yes | antibody |
| **Tacrolimus** | Peptidyl-prolyl cis-trans isomerase FKBP1A | FKBP1A | P62942 | Yes | inhibitor |
| **Anifrolumab** | Interferon alpha/beta receptor 1 | IFNAR1 | P17181 | Yes | inhibitor |
| **Voclosporin** | Calcium signal-modulating cyclophilin ligand | CAMLG | P49069 | Yes | Binder |
|  | Calcineurin subunit B type 1 | PPP3R1 | P63098 | Yes | inhibitor |
|  | Calcineurin subunit B type 2 | PPP3R2 | Q96LZ3 | Yes | inhibitor |

**Supplementary Table 6 Current medications targeting eight potential causal proteins**

| Target | Uniprot ID | Drugbank ID | Medication | Pharmacological action | Indication | Actions |
| --- | --- | --- | --- | --- | --- | --- |
| PPP3CA; PPP3R1 | P63098 | DB11693 | Voclosporin | unknown | Voclosporin is used in combination with a background immunosuppressive regimen for the treatment of lupus nephritis. Safety has not been established in combination with cyclophosphamide. | NA |
|  |  | DB08231 | Myristic acid | yes | NA | Inhibitor |
| ICAM1/sICAM-1 | P05362 | DB00108 | Natalizumab | unknown | Natalizumab is indicated as monotherapy for the treatment of relapsing forms of multiple sclerosis, including clinically isolated syndrome, relapsing-remitting disease, and active secondary progressive disease in adults. It is also indicated for inducing and maintaining clinical response and remission in adult patients with moderately to severely active Crohn’s disease with evidence of inflammation who have had an inadequate response to or are unable to tolerate, conventional therapies and inhibitors of TNF-α. It is not to be used in combination with immunosuppressants or inhibitors of TNF-α. | ligand |
|  |  | DB08818 | Hyaluronic acid | yes | The intra-articular preparations of hyaluronic acid are indicated for knee pain associated with osteoarthritis. Hyaluronic acid is used in cosmetic applications to prevent and reduce the appearance of wrinkles on the face, and as a dermal filler to correct facial imperfections or other imperfections on other parts of the body. It is frequently an ingredient in topical applications for wound healing and symptomatic treatment of skin irritation from various causes. Hyaluronic acid may also be indicated in ophthalmological preparations or oral capsules to treat discomfort caused by dry eyes or conjunctivitis and for its protective qualities during and before eye surgery. Finally, hyaluronic acid can be used off-label to coat the bladder for relief of interstitial cystitis symptoms. | inhibitor binder |
|  |  | DB12598 | Nafamostat | unknown | Used as an anticoagulant in patients with disseminative blood vessel coagulation, hemorrhagic lesions, and hemorrhagic tendencies. It prevents blood clot formation during extracorporeal circulation in patients undergoing continuous renal replacement therapy and extra corporeal membrane oxygenation. | Inhibitor |
| FCG2B | P31994 | DB00054 | Abciximab | unknown | Abciximab is indicated as an adjunct to percutaneous coronary intervention for the prevention of cardiac ischemic complications in patients undergoing percutaneous coronary intervention and in patients with unstable angina not responding to conventional medical therapy when percutaneous coronary intervention is planned within 24 hours. Abciximab is intended for use with aspirin and heparin and has been studied only in that setting. | NA |
|  |  | DB00005 | Etanercept | unknown | Etanercept is indicated for the treatment of moderately to severely active rheumatoid arthritis in adults and in chronic moderate to severe plaque psoriasis in patients 4 years of age and older. It is also used to manage signs and symptoms of polyarticular idiopathic arthritis and Juvenile Psoriatic Arthritis in those aged 2 years and older. Etanercept is also used to manage the symptoms of psoriatic arthritis and ankylosing spondylitis | ligand |
|  |  | DB00087 | Alemtuzumab | unknown | LEMTRADA is indicated for the treatment of relapsing forms of multiple sclerosis (MS), including relapsing-remitting disease and active secondary progressive disease, in adults. Because of its safety profile, the use of LEMTRADA should generally be reserved for patients who have had an inadequate response to two or more drugs indicated for the treatment of MS. LEMTRADA contains the same active ingredient (alemtuzumab) found in CAMPATH, and CAMPATH is approved for the treatment of B-cell chronic lymphocytic leukemia (B-CLL), although generally administered at higher and more frequent doses (e.g., 30 mg) than recommended in the treatment of MS. | binder |
|  |  | DB00081 | Tositumomab | unknown | For treatment of non-Hodgkin's lymphoma (CD20 positive, follicular) | NA |
| NID2 | Q14112 | NA | NA | NA | NA | NA |
| N-terminal pro-BNP | P16860 | DB00325 | Nitroprusside | yes | For immediate reduction of blood pressure of patients in hypertensive crises, reduce bleeding during surgery, and for the treatment of acute congestive heart failure | agonist |
|  |  | DB00727 | Nitroglycerin | unknown | Sublingual nitroglycerin is indicated for the acute relief of an attack or acute prophylaxis of angina pectoris due to coronary artery disease. Transdermal nitroglycerin is indicated for the prevention of angina pectoris due to coronary artery disease. Intravenous nitroglycerin is indicated for the treatment of peri-operative hypertension; for control of congestive heart failure in the setting of acute myocardial infarction; for treatment of angina pectoris in patients who have not responded to sublingual nitroglycerin and beta (β)-blockers; and for induction of intraoperative hypotension. Topical nitroglycerin ointment is used to treat moderate to severe pain associated with chronic anal fissure. | agonist |
|  |  | DB01613 | Erythrityl tetranitrate | yes | For the prevention of angina. | agonist |
| Layilin | Q6UX15 | DB08818 | Hyaluronic acid | unknown | The intra-articular preparations of hyaluronic acid are indicated for knee pain associated with osteoarthritis. Hyaluronic acid is used in cosmetic applications to prevent and reduce the appearance of wrinkles on the face, and as a dermal filler to correct facial imperfections or other imperfections on other parts of the body. It is frequently an ingredient in topical applications for wound healing and symptomatic treatment of skin irritation from various causes. Hyaluronic acid may also be indicated i ophthalmological preparations or oral capsules to treat discomfort caused by dry eyes or conjunctivitis and for its protective qualities during and before eye surgery. Finally, hyaluronic acid can be used off-label to coat the bladder for relief of interstitial cystitis symptoms. | binder |
| Prekallikrein | P03952 | DB05311 | Ecallantide | yes | Indicated for the symptomatic treatment of acute attacks of hereditary angioedema (HAE) in patients 12 years of age and older | Inhibitor |
|  |  | DB06404 | Human C1-esterase inhibitor | yes | Intravenous and subcutaneous formulations of the human C1-esterase inhibitor are indicated for routine prophylaxis against acute attacks of hereditary angioedema in patients six years of age and older. It is also used to treat these in adult and adolescent patients with hereditary angioedema. | Inhibitor |
|  |  | DB09228 | Conestat alfa | yes | For the treatment of acute attacks of hereditary angioedema (HAE) due to C1 esterase inhibitor deficiency in adults. | Inhibitor |
|  |  | DB01593 | Zinc | unknown | Zinc can be used for the treatment and prevention of zinc deficiency/its consequences, including stunted growth and acute diarrhea in children, and slowed wound healing. It is also utilized for boosting the immune system, treating the common cold and recurrent ear infections, as well as preventing lower respiratory tract infections. | NA |
